# Proficient pyrimidine-based heterocyclic chemistry in medicine: advances in synthesis, biological, and SAR studies: a review

**DOI:** 10.1039/d6ra04538c

**Published:** 2026-07-07

**Authors:** Basant Farag, Mohamed R. Fouad, Sampath Chinnam, Aamer Saeed, Ghada G. El-Bana, Sobhi M. Gomha

**Affiliations:** a Department of Chemistry, Faculty of Science, Zagazig University Zagazig 44519 Egypt basantfarag@zu.edu.eg; b Department of Pesticide Chemistry and Technology, Faculty of Agriculture, Alexandria University Aflaton St., El-Shatby Alexandria 21545 Egypt mohammed.riad@alexu.edu.eg; c Department of Chemistry, M.S. Ramaiah Institute of Technology (Autonoumous Institution, Affiliated to Visvesvaraya Technological University, Belgaum) Bengaluru Karnataka 560054 India sampathchinnam@gmail.com; d Department of Chemistry, Quaid-i-Azam University Islamabad 45320 Pakistan asaeed@qau.edu.pk; e Department of Chemistry, Faculty of Science, Mansoura University El-Gomhoria Street Mansoura 35516 Egypt ghadaelbana@mans.edu.eg; f Mansoura University Student's Hospital, Mansoura University El-Gomhoria Street Mansoura ET-35516 Egypt; g Department of Chemistry, Faculty of Science, Islamic University of Madinah Madinah 42351 Saudi Arabia smgomha@iu.edu.sa

## Abstract

As the most varied class of organic compounds, N-heterocycles—especially pyrimidine derivatives, which are found in many remarkable synthetic substances—have important chemical, biological, and industrial uses. This review sheds light on a number of techniques for building diverse fused pyrimidine scaffolds using N-heterocycle moieties as adaptable precursors. Recent studies demonstrate the promise of pyrimidine-based heterocyclic hybrids, particularly those with bicyclic (pyrido[2,3-*d*]pyrimidine and pyrano[2,3-*d*]pyrimidine) and tricyclic (pyrido[2,3-*d*:5,6-*d*′]dipyrimidine) moieties that exhibit enhanced cytotoxicity and target selectivity. Because they developed N-heterocyclic-based pyrimidine analogous frameworks in a single reaction step, multicomponent reactions are crucial in the advanced, proficient synthesis of medications. Green along with ecofriendly protocols for syntheses of such organic moieties are essential for sustainable growth of the pharmaceutical and agrochemical fields. This review highlights the structure–activity relationships guiding the advancement for next-generation pyrimidine pharmaceutical chemotypes and embraces various synthetic strategies and summarizes recent research developments, as well as reveals the many important biological characteristics (anticancer, *in vitro* α-amylase inhibitory, antimicrobial, antiviral, anti-inflammatory, antioxidant, antituberculosis, antidiabetic, anticholinesterase, antiplatelet, and *in vitro* antimalarial agents).

## Abstract 2

As the most varied class of organic compounds, N-heterocycles—especially pyrimidine derivatives, which are found in many remarkable synthetic substances—have important chemical, biological, and industrial uses.

## Graphical abstract

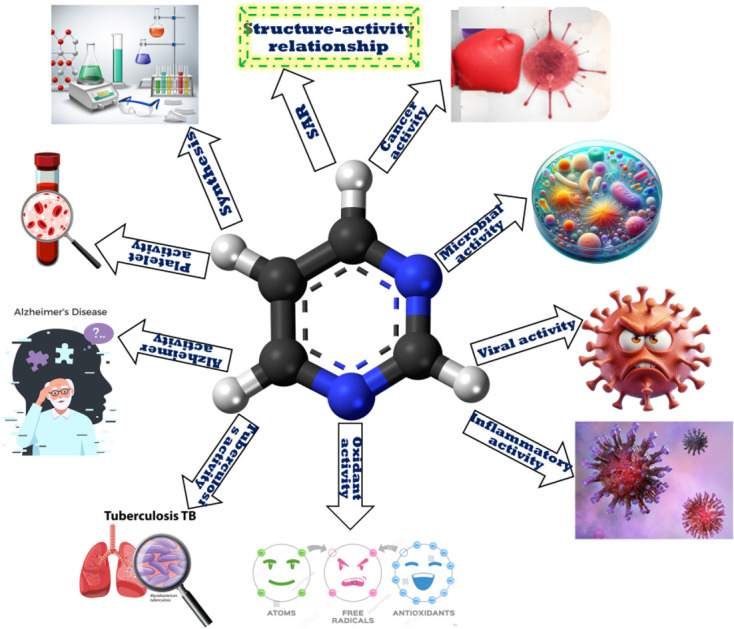

## 1 Introduction

N-Heterocycles, which are the majority of biologically active chemicals, have garnered significant interest from researchers due to their intriguing biological characteristics.^1–3^ Green chemistry, microwave irradiation, and other eco-friendly techniques were used to achieve these molecules, which researchers in synthetic chemistry are very interested.^4,5^ One-pot multicomponent reaction (MCR) techniques are useful and efficient laboratory instruments used by organic and pharmaceutical chemists for a quick and environmentally friendly way to produce molecular variety and complexity from simple and easily accessible substrates. For advanced, proficient synthesis of these target scaffolds, environmentally safe procedures have been investigated to increase reaction yields and energy consumption.^6,7^ Furthermore, MCRs usually refer to time-saving and selective.^8,9^

Pinner's discovery, which initially referred to the unsubstituted parent unit as pyrimidine, marked the beginning of the systematic study of the ring system.^10,11^ The nitrogen atoms in the first and third positions of the six-membered pyrimidine or *m*-diazine ring function as a flexible structural framework that permits different substitutions to increase its pharmacological activity.^12^ The two nitrogen atoms' ability to attract electrons greatly influences the characteristics of pyrimidines; each of these enhances the electronic effect of the other in the 2-, 4-, and 6-positions. The 5-position is a target for electrophilic attack because it is only impacted by the inductive effect of the two nitrogen atoms.

Uracil and its derivatives have numerous uses, including anticancer,^13–15^ antibacterial,^16,17^ antiviral,^18^ and anti-inflammatory^19,20^ along with antioxidant^13,21^ characteristics. 6-Aminouracil and its derivatives are frequent subunits in many cyclic compounds and attractive scaffolds in organic science. Many scientists are interested in this molecule since it can function as both an electrophile and a nucleophile. Fused annulated compounds with polycyclic compounds, such as bicyclic (pyrido[2,3-*d*]pyrimidine and pyrano[2,3-*d*]pyrimidine) and tricyclic (pyrido[2,3-*d*:5,6-*d*′]dipyrimidine) moieties, can be effectively synthesized with these scaffolds.^22–24^

The manufacturing of fused pyrimidines is consequently very important for the fusion of organic N-heterosystems. A remarkably wide range of pharmacological and biological activities have been demonstrated by these derivatives,^25^ such as antimicrobial,^26–29^*in vitro* α-amylase inhibitory,^30^ anticancer,^17,31–33^ antiviral,^34–37^ anti-inflammatory,^38,39^ antioxidant,^40,41^ antituberculosis,^42^ and antiplatelet.^43^ Among these were pyrido[2,3-*d*]pyrimidines, pyrano[2,3-*d*]pyrimidine, as well as pyrido[2,3-*d*:5,6-*d*′]dipyrimidine.

The ortho-fusion of the pyridine and pyrimidine rings yields pyridopyrimidines, which are known to exist in a number of classes of potential isomeric forms, including derivatives of pyridopyrimidines. The numerous pharmacological uses for analogues of pyrido[2,3-*d*]pyrimidines involve chemotherapy drugs,^44,45^ antimicrobial agents,^46–49^ antiviral agents,^50,51^ antioxidant agents,^21^ antituberculosis agents,^52^ antidiabetic agents,^53^ and anticholinesterase agents,^54^ which have drawn significant attention from medicinal chemists.

Numerous synthesis techniques and therapeutic uses for N-heterocyclic-based pyrimidine derivatives are revealed in this review. Therefore, it is very important to review these scaffolds in pharmaceutical drug discovery since it unites the most recent information on structural variety and biological activity. By identifying important structure–activity relationships, potential pharmacophores, and appropriate molecular targets, these analyses aid in the logical advancement of substances that are more potent and selective. To the best of our knowledge, this is helpful to researchers studying pharmacology, medicinal chemistry, and synthetic organic chemistry.

## 2 Materials and methods

Using specified keywords associated with N-heterocycle-based pyrimidines and their pharmacological characteristics with their SAR, a comprehensive investigation was conducted in the following databases: Science Direct, WOS, PubMed, Google Scholar, and Scopus. Included were articles published between 1951 and 2026. Following careful title, abstract, and full-text screening, important information was retrieved from the studies that were chosen using scheduled inclusion-exclusion criteria, as presented in Table 1.

**Table 1:** Inclusion-exclusion criteria

Inclusion criteria	Exclusion criteria
Publication type: peer-reviewed journal articles, research indexed in Scopus/WOS.	Non-indexed sources: grey literature, theses, or non-peer-reviewed research
Publication year: within the defined publication years (1951–2026)	Incomplete data: articles lacking quantitative results or methodological details
Study design: experimental studies reporting quantitative outcomes (IC_50_, MIC, % inhibition, SAR)	Irrelevant scope: studies outside chemistry/biomedicine (*e.g.*, corrosion studies not linked to specification of interest)
Relevance: direct focus on the nucleus class, assay, or structure–activity relationship	Duplicate publications: same dataset reported in multiple venues
Language: English	Language: publications are not available in English if translation resources are limited

## 3 Discussion

The preparation of N-heterocyclic rings formed from pyrimidine, uracil and its analogous, barbituric acid, pyrido[2,3-*d*]pyrimidine, and pyrano[2,3-*d*]pyrimidine, as well as pyrido[2,3-*d*:5,6-*d*′]dipyrimidine, along with their biological evaluations and their SAR, are the subject of the work represented in this review. The discussion is subsequently given in the following two items and their subcategories.

### 3.1 Chemistry of pyrimidine and fused pyrimidine derivatives

#### 3.1.1 Synthesis and reactions of 1,3-dinitrogen atoms' heterocyclic derivatives

##### 3.1.1.1 Synthesis of pyrimidine

**Scheme 1:**
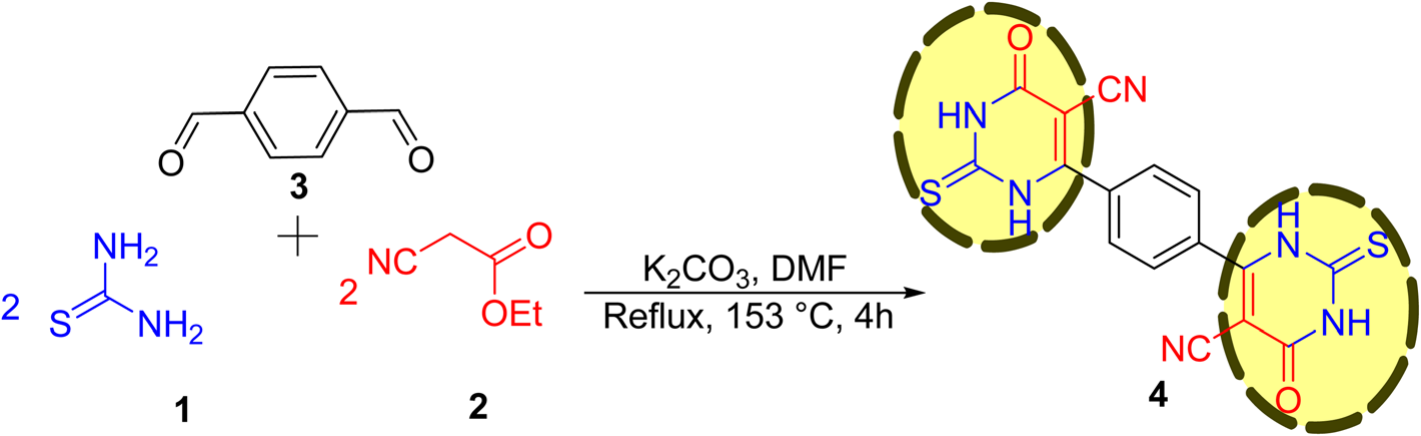
Bis-pyrimidine 4 synthesis.

As seen in Scheme 1, one mole of terephthalaldehyde (3), two moles of ethyl cyanoacetate (2), and then two moles of thiourea (1) were combined with DMF (30 mL) and heated under reflux at 153 °C for four hours in the presence of potassium carbonate to produce a bis-pyrimidine 4.^55^

**Scheme 2:**
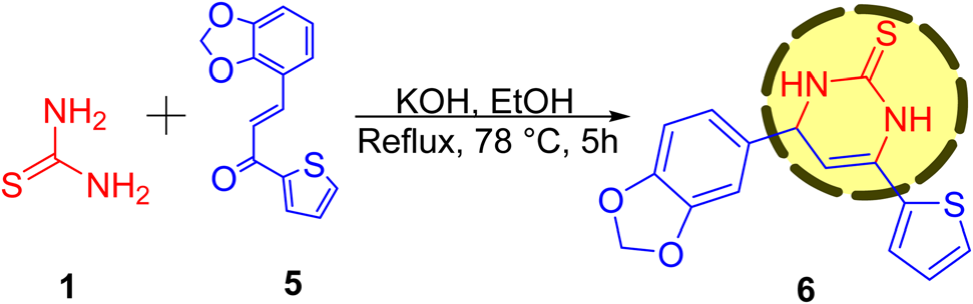
Advanced for pyrimidine scaffold 6.

Scheme 2 illustrates how a binary combination of potassium hydroxide (8 mmol), thiourea (1) (5 mmol), and chalcone 5 (5 mmol) in absolute ethanol (20 mL) was heated at reflux at 78 °C for five hours to afford the hybrid of pyrimidine-2-thione 6.^56^

**Scheme 3:**
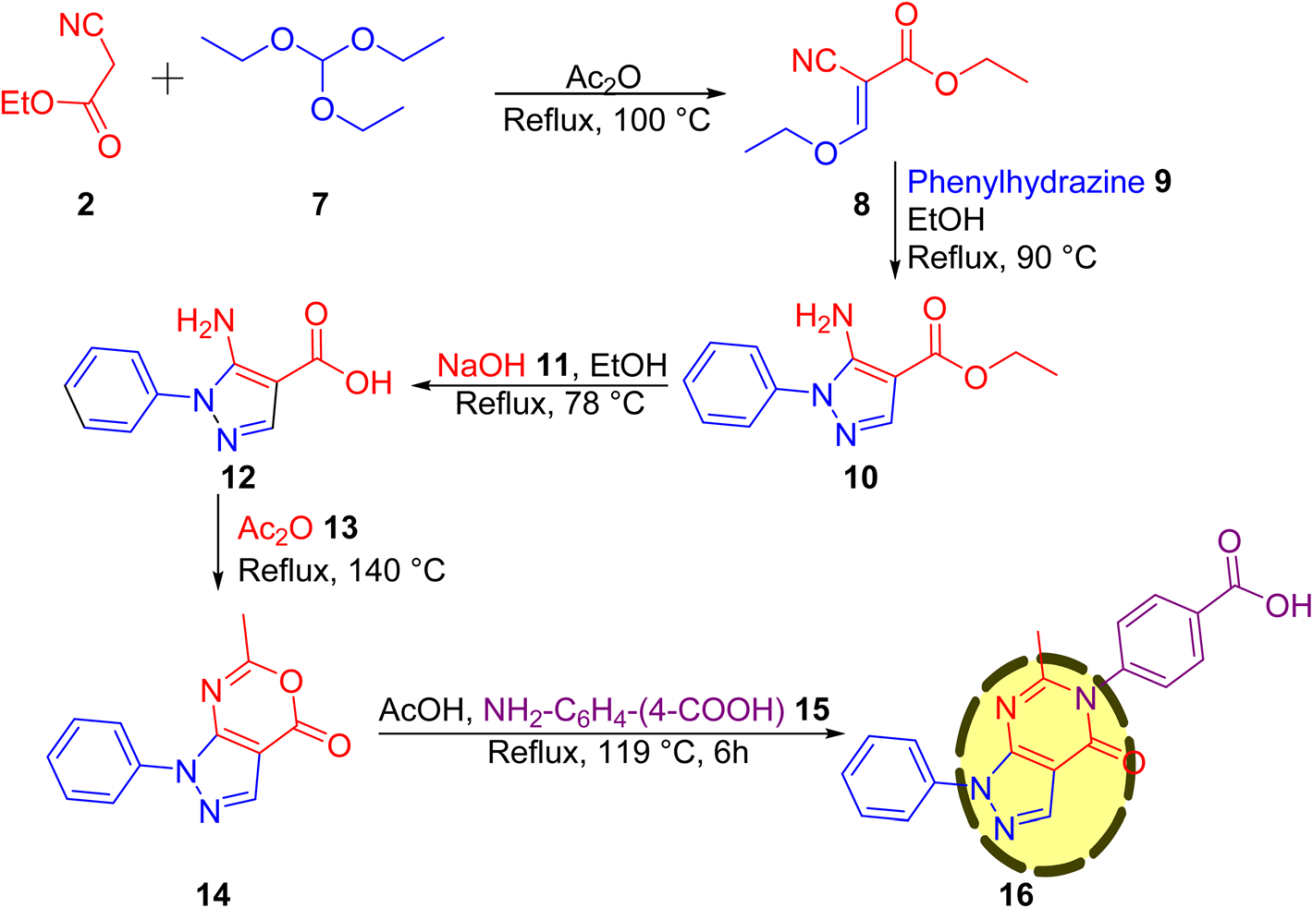
Synthetic steps for the targeted compound 16.

Based on the procedures that have been outlined,^57,58^ the primary and intermediates substances ethyl 2-cyano-3-ethoxyacrylate (8), ethyl 5-amino-1-phenyl-1*H*-pyrazole-4-carboxylate (10), 5-amino-1-phenyl-1*H*-pyrazole-4-carboxylic acid (12), and 6-methyl-1-phenylpyrazolo[3,4-*d*][1,3]oxazin-4(1*H*)-one (14) were established. 14 (0.01 mol) in AcOH (20 mL) was refluxed at 119 °C for six hours with an equimolar quantity of 4-aminobenzoic acid (15), affording the target compound 16, as shown in Scheme 3.^59^

##### 3.1.1.2 Synthesis of uracil

General methods in order to synthesis uracil: —

###### 3.1.1.2.1 From urea and cyanoacetylene

**Scheme 4:**
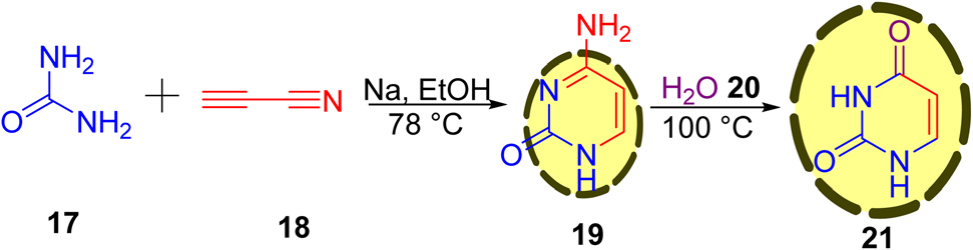
The primary syn. of the uracil nucleus is 19 and its modification is 21.

The urea (17) (0.01 mol) is treated with cyanoacetylene (18) (0.01 mol) in sodium ethoxide (20 mL) and refluxed at 78 °C to produce cytosine (19). Cyanoacetylene (18) is one of the main nitrogen-containing components of an electric discharge acting on a nitrogen and methane combination. Given that cytosine (19) (0.01 mol) is hydrolyzed by water (20 mL) and heated at 100 °C to uracil (21), these processes result in the prebiotic formation of pyrimidine (Scheme 4).^60–62^

###### 3.1.1.2.2 From enriched urea and propiolic acid

**Scheme 5:**
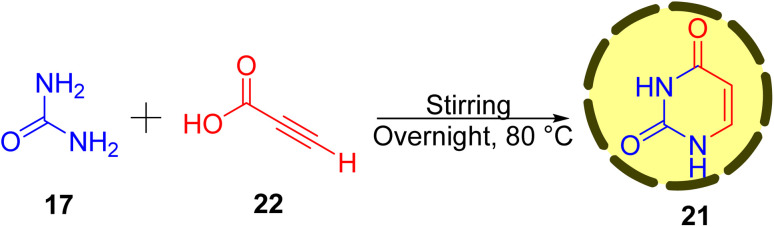
The synthetic route of uracil hybrid 21 by Harada and Suzuki's method.

Harada and Suzuki's method was made to synthesis uracil (21) by stirring, a mixture of ^15^N enriched urea (17) and propiolic acid (22) (1.2 equiv.) at a temperature of 80 °C overnight (Scheme 5). Filtration yielded a 72% recovery of crystalline ^15^N-enriched uracil. Uracil crystals started to form as quickly as possible, and the solution was kept at 4 °C for the entire night.^63^

###### 3.1.1.2.3 From urea and maleic acid

**Scheme 6:**
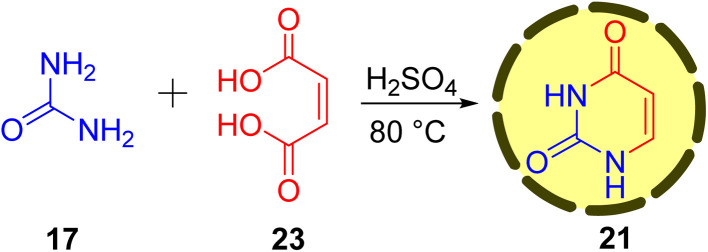
Syn. of uracil 21 by Ramesh *et al*.^64^

Scheme 6 explains how urea (17) and maleic acid (23) (1.2 equiv.) react to produce uracil (21) within the corporation with sulfuric acid (5 mL) under stirring at a temperature of 80 °C overnight.^64^

##### 3.1.1.3 Synthesis of 6-aminouracil

###### 3.1.1.3.1 From urea and ethyl cyanoacetate

**Scheme 7:**
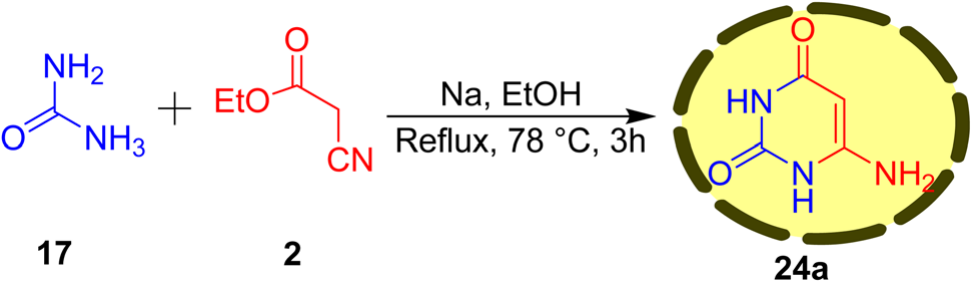
Syn. of the 6-aminouracil 24a.

The condensation of urea (17) (0.013 mol) and ethyl cyanoacetate (2) (0.013 mol) as well as a freshly prepared solution of sodium ethoxide (Na, 0.013 mol, in EtOH, 20 mL) at 78 °C afforded 6-aminouracil (24a) (Scheme 7).^65^

##### 3.1.1.4 Synthesis of barbituric acid

###### 3.1.1.4.1 From urea and diethyl malonate

**Scheme 8:**
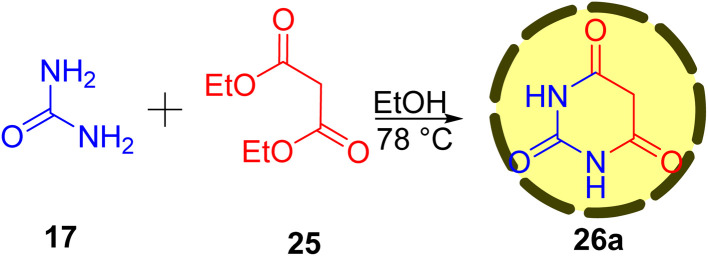
Syn. of target barbituric acid (26a).

The cyclocondensation of urea (17) (0.013 mol) and diethyl malonate (25) (0.013 mol) in absolute ethanol (20 mL) at 78 °C afforded barbituric acid (26a) (Scheme 8).^66^

##### 3.1.1.5 Reactions of uracil derivatives

**Scheme 9:**
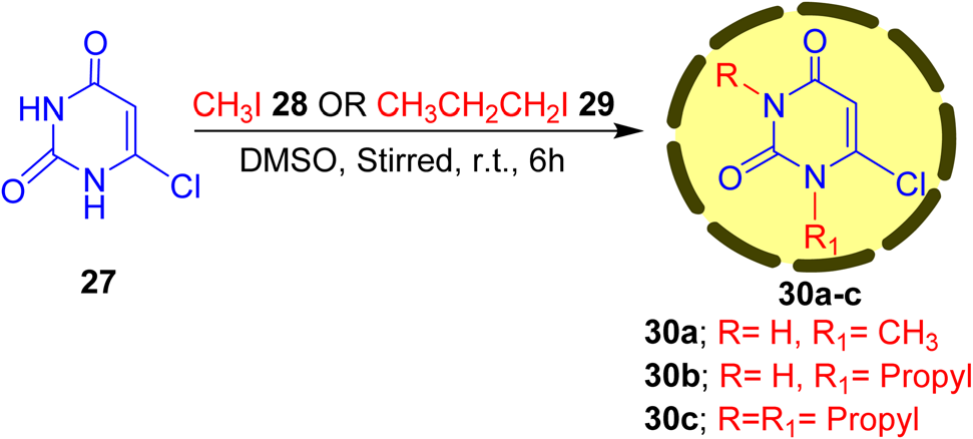
Alkylation Rx. of 6-chlorouracil (27).

Scheme 9 was followed; regioselective alkylation of 6-chlorouracil (27) (40 mmol) with methyl and/or propyl iodide (28 and 29, respectively) (40 mmol) in 20 mmol of potassium carbonate as a basic media in DMSO (25 mL) yielded alkylated uracil 30a–c. 6-Chlorouracil was heated gently until dissolved, then potassium carbonate was added with stirring. The alkylated agent was added one time, and the mixture was stirred at room temperature for 6 hours.^67^

**Scheme 10:**
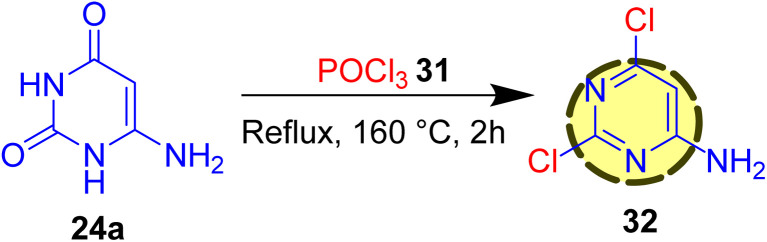
The chloration Rx. Of 6-aminouracil (24a).

Scheme 10 was followed for the reaction of certain uracil moiety. For more than a century, the simple approach of hydroxypyrimidine reacting with phosphorous oxychloride (POCl_3_) has been frequently utilized to produce the resulting compounds of chlorinated pyrimidines.^68^ Improvements in reducing the amount associated with POCl_3_ used in massive operations chlorination procedures can be noteworthy throughout economic, environmental, and safety reasons.^69^ Equimolar POCl_3_ may effectively chlorinate hydroxypyrimidine as well as a variety of other substrates. In accordance with the described procedure, compound 32 was made in the lab by heating 6-aminouracil (24a) (0.3 moles) under reflux with phosphorous oxychloride (31) (0.6 moles, 1 equivalent POCl_3,_ per reactive OH). The reaction mixture was refluxed at 160 °C for 2 hours.^70^

**Scheme 11:**
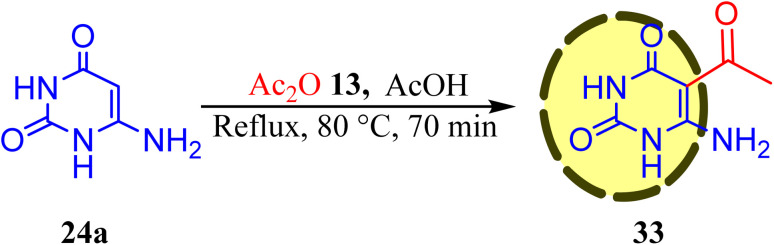
Acetylation Rx of 6-aminouracil (24a).

However, due to solubility problems, the particular reaction conditions of Pfleiderer^71^ were not applicable to the other compounds. It was discovered that anhydride-acetic acid combinations could potentially be used to solve solubility difficulties. Functionalized molecule 24a (100 mg) was refluxed in an acidic mixture of acetic acid (15 mL) and acetic anhydride (13) (5 mL) for approximately seventy minutes at eighty degrees celsius to produce 5-acetyl-6-aminouracil (33) (Scheme 11).^14,72^

**Scheme 12:**
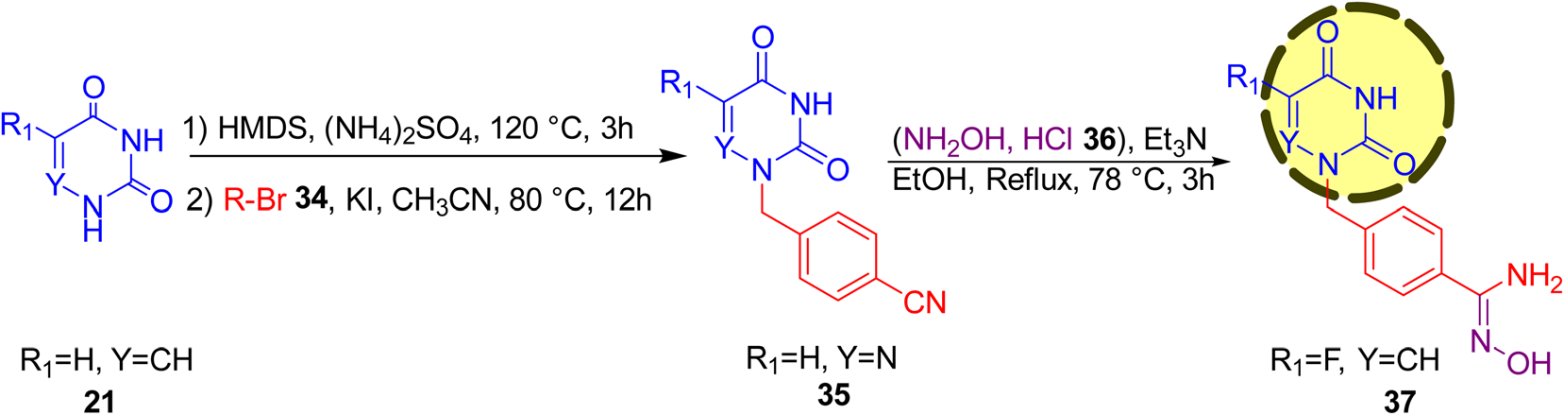
Synthetic route for the compound amidoxime 37.

A mixture of the appropriate pyrimidine 21 (1 mmol) and ammonium sulfate (0.1 mmol) in hexamethyldisilazane (HMDS) (1.5 mL) was refluxed at 120 °C until a clear solution was obtained. Then 4-(bromomethyl)benzonitrile (34) (1.5 mmol), KI (0.75 mmol), and acetonitrile (2.5 mL) were added. The reaction mixture was heated at 80 °C for 12 hours. Amidoxime 37 was produced by treating 35 (0.41 mmol) with hydroxylamine hydrochloride (36) (0.615 mmol) and trimethylamine (0.615 mmol) in ethanol (2 mL) under reflux at 78 °C (Scheme 12).^73^

**Scheme 13:**
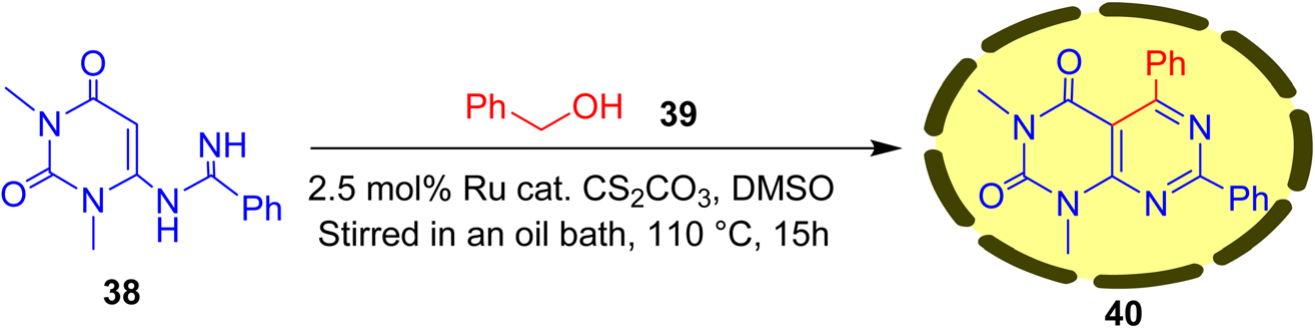
Chemical synthesis methodology of *N*-uracil benzamidine 38 with phenyl methanol (39).

Scheme 13 shows that it's reasonable to suppose that alcohols 39 (0.75 mmol) with *N*-uracil benzamidine 38 (0.5 mmol) process the pyrimidouracil 40. 1 mL of DMSO was added by syringe, and the tube was sealed without inserting any gas protection. The accessible at any time, inexpensive aryl methanol 39 was initially used in the manufacture of *N*-uracil amidine 40 using 2.5 mol% [RuCl_2_(*p*-cymene)]_2_ and CS_2_CO_3_ (0.75 mmol) as an effective catalytic system. The reaction mixture was stirred in an oil bath at 110 °C for 15 hours. This synthetic approach is applicable to a wide range of alcoholic substrates and does not require protection throughout the entire preparation process because alcohols are more stable than aldehydes. The methodology that is being given has the potential to prepare important items that are now out of stock or very difficult to obtain by following standard practice. As a result, this methodology is significantly more effective than today's methods.^74^

#### 3.1.2 Synthesis of pyrido[2,3-*d*]pyrimidine analogous

Pyrido[2,3-*d*]pyrimidines have recently gotten a lot of attention from multi-component synthetic protocols and medicinal chemists. Drugs produced from pyrido[2,3-*d*] pyrimidine exhibit many pharmacological characteristics,^75^ including anti-inflammatory,^76^ cytotoxic,^77^ and antimicrobial.^78^

##### 3.1.2.1 General methods for pyrido[2,3-*d*]pyrimidine synthetic

**Scheme 14:**
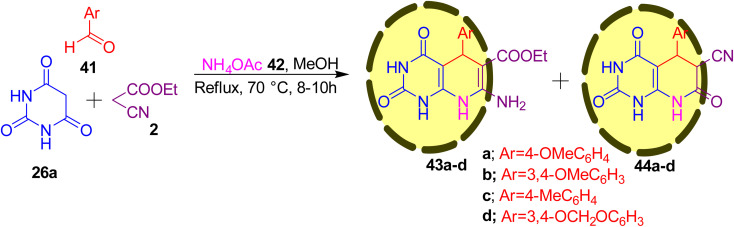
Pyrido[2,3-*d*]pyrimidine 43a–d as well as 44a–d synthesis *via* four-component cyclocondensation.

According to the initial Knoevenagel, subsequent Michael, and final heterocyclization reactions, Scheme 14's four-component one-pot cyclocondensation of a mixture of equimolar amounts (0.01 moles) of aromatic aldehydes 41, ethyl cyanoacetate (2), barbituric acid (26a), and ammonium acetate (42) in methanol was refluxed on a water bath at 70 °C for 8–10 hours and produced alternative and functionalized pyrido[2,3-*d*]pyrimidine analogs 43a–d as well as 44a–d.^79^

**Scheme 15:**
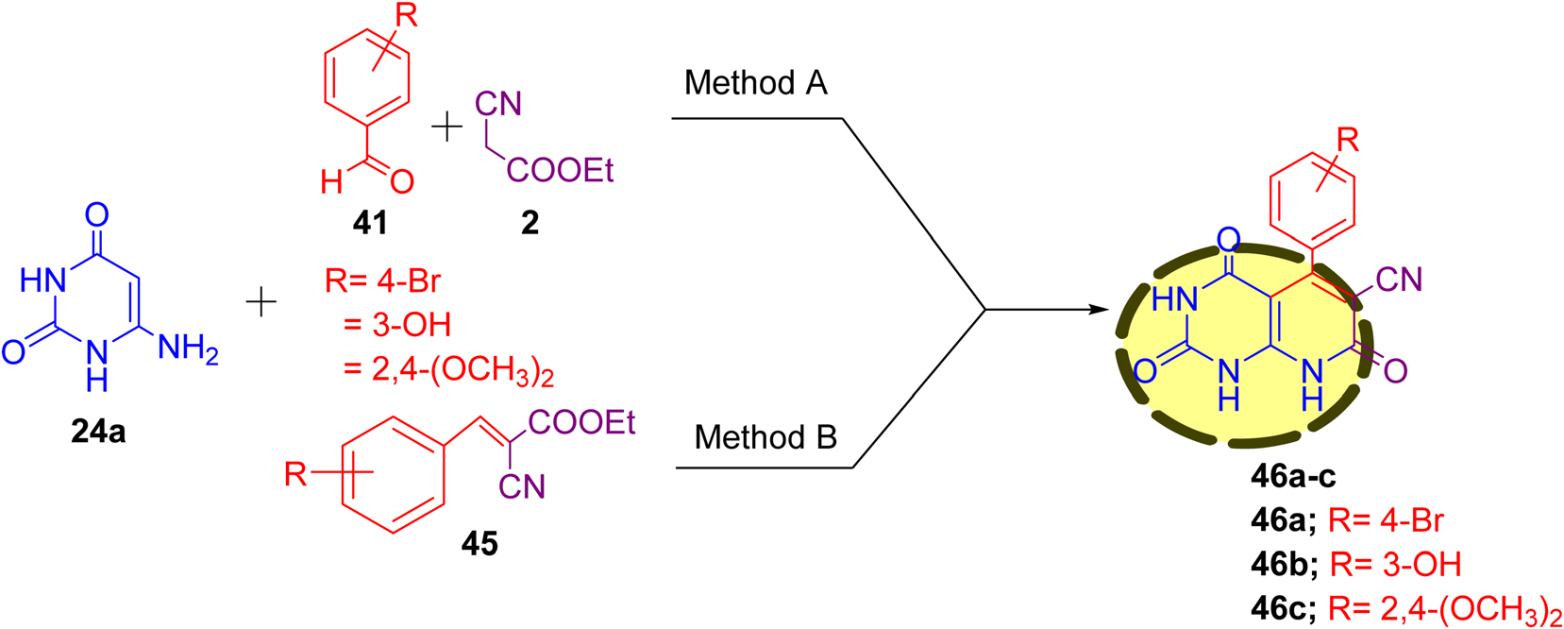
Synthesis of 6-cyano-7-oxopyrido[2,3-*d*]pyrimidine analogous 46a–c by one-pot techniques.

Scheme 15 displayed the procedure used to synthesize compounds 6-cyano-7-oxopyrido[2,3-*d*]pyrimidine 46a–c. One-pot processes using equivalent proportions of 24a (2 mmol), the relevant aldehyde 41 (2 mmol), and ethyl cyanoacetate (2) (2 mmol) and absolute ethanol (20 mL) were heated under reflux at 78 °C for 4 hours (method A), or a reaction of 24a (2 mmol) with the benzylidene analogues of ethyl cyanoacetate 45 (2 mmol) and absolute ethanol (15 mL) was heated under reflux at 78 °C for 2 hours (method B), which were the synthetic routes utilized.^80^

**Scheme 16:**
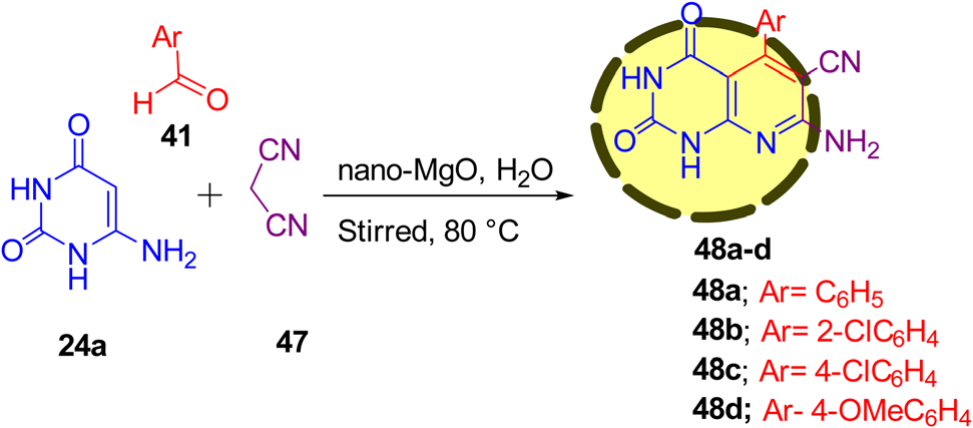
Green advancement of pyrido[2,3-*d*]pyrimidine 48a–d analogues using MgO nanocrystals.

The 6-aminouracil (24a) (1 mmol) condensation with arylaldehydes 41 (1 mmol) and malononitrile (47) (1 mmol) to create pyrido[2,3-*d*]pyrimidine analogues 48a–d was stirred efficiently over MgO nanocrystalline (0.25 mmol) at about eighty degrees celsius while using water (5 mL) as a green solvent (Scheme 16). By avoiding hazardous catalysts and solvents, this technology enables notable benefits to support the development of pyrido[2,3-*d*]pyrimidine compounds 48a–d in terms of product yield, ease of use, and green features.^81^

**Scheme 17:**
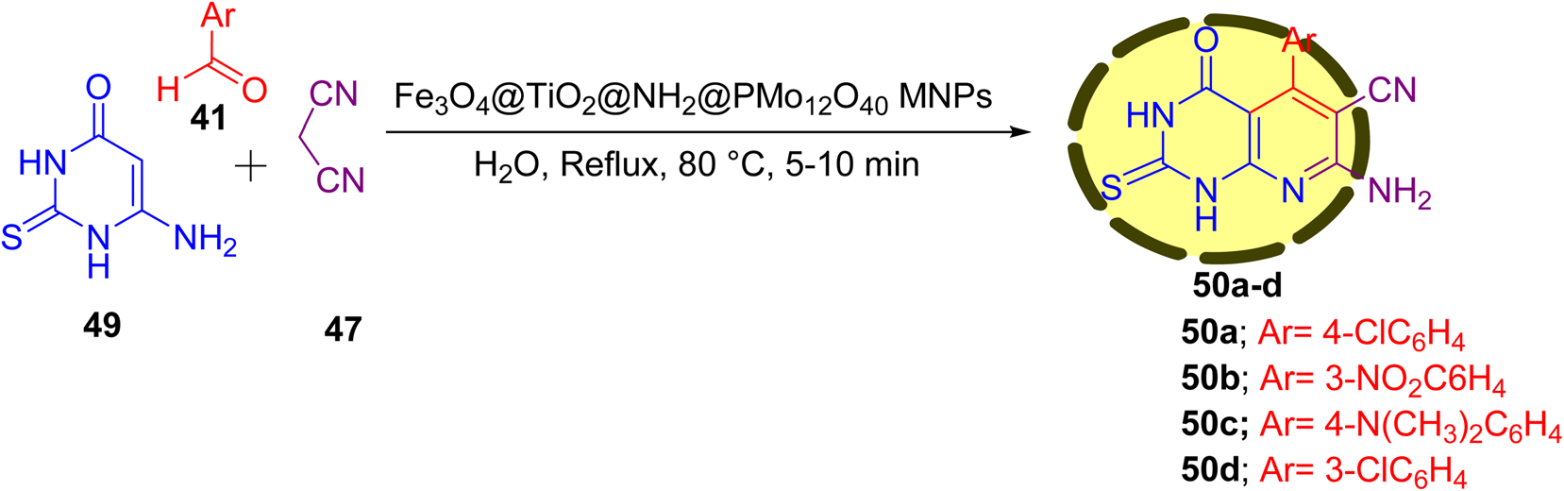
Catalytic advancement of pyrido[2,3-*d*]pyrimidine 50a–d analogous.

Regarding 6-amino-2-thiouracil (49) (1 mmol), malononitrile (47) (1 mmol), and aldehydes 41 (1 mmol) were carried out using the 10 mg of catalyst Fe_3_O_4_@TiO_2_@NH_2_@PMo_12_O_40_. This produced a variety of pyrido[2,3-*d*]pyrimidine substitutes 50a–d (Scheme 17) within a significant yield (92–98%) along with a brief reaction period (5–10 min) under reflux at 80 °C in water (5 mL). Eight batches of the catalyst were utilized and recovered with no noticeable decrease in catalytic function.^82^

**Scheme 18:**
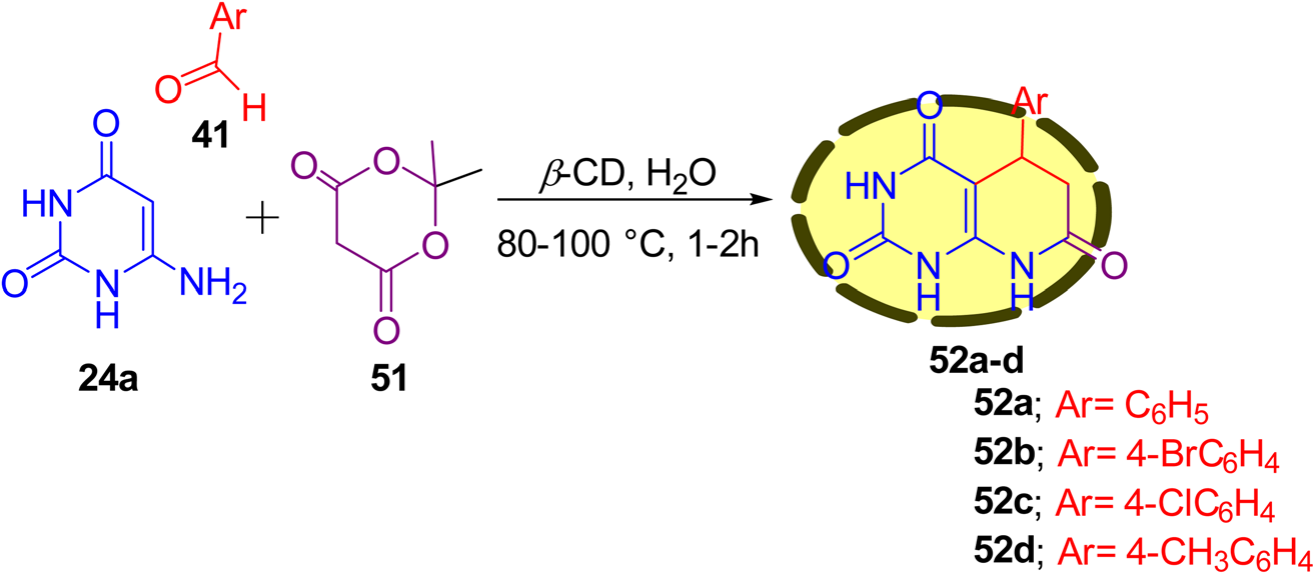
Green development of fused bicyclic pyrimidine analogous 52a–d*via* a three-component reaction.

Scheme 18 shows the usual Meldrum's acid (51) (1 mol) was used to initiate a three-component reaction between derivatives of benzaldehyde 41 (1 mol) along with 6-aminouracil (24a) (1 mol) in an aqueous medium. This reaction was accelerated by 15 mol% β-cyclodextrin, which produced 52a–d in higher yields (85–92%) in a single reaction procedure with an air- and humidity-maintained catalyst in water (5 mL) as a green solvent under heating conditions (80–100 °C). The current method lays the basis for the building of medically attractive structural scaffolds and offers benefits when combined, involving low catalyst dosage and simple purification procedures. The utilized β-cyclodextrin catalyst was recovered and replicated multiple times without experiencing a significant decrease in catalytic activity, a vital feature of green chemistry.^83^

**Scheme 19:**
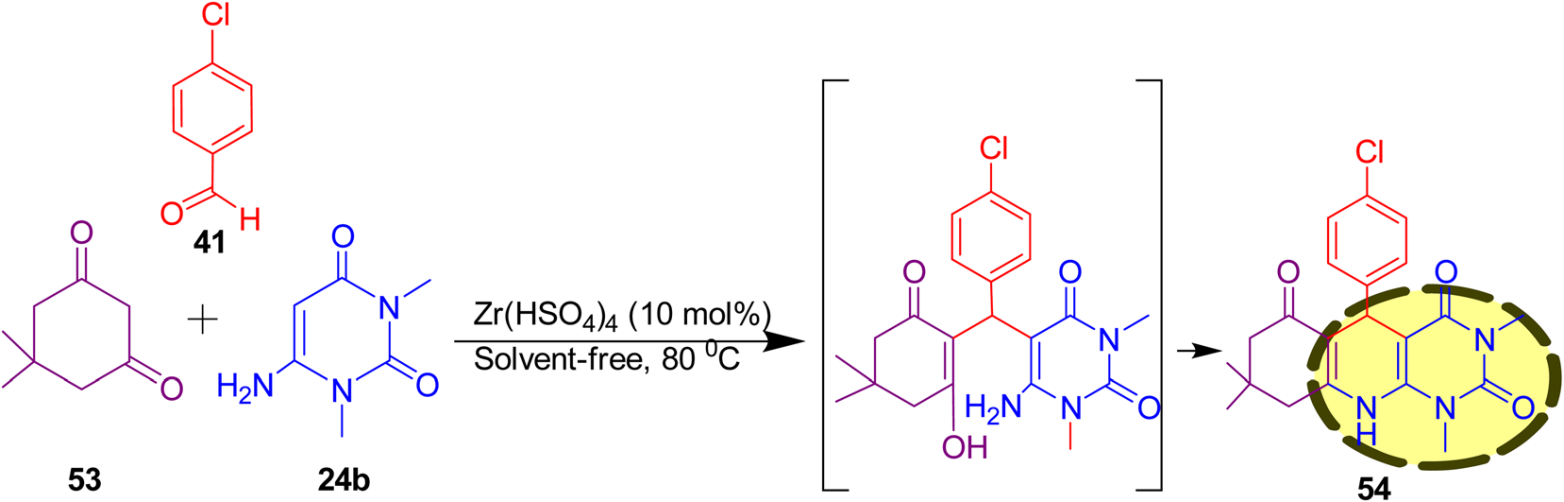
One-pot development of pyridopyrimidine 54 using the Zr(HSO_4_)_4_ catalyst.

5-(4-Chlorophenyl)-1,3,8,8-tetramethyl-5,8,9,10-tetrahydropyrimido[4,5-*b*]quinoline-2,4,6(1*H*,3*H*,7*H*)-trione (54) is produced by a simple one-pot, three-component reaction of aromatic aldehyde 41 (1 mmol), 6-amino-1,3-dimethyluracil (24b) (1 mmol), and active methylene compound 53 (1 mmol) regarding the addition of Zr(HSO_4_)_4_ (10 mol%) as a heterogeneous catalyst under solvent-free circumstances, stirred at 80 °C for 1 hour (Scheme 19). This method offers a number of benefits, including easy process construction, solvent-free reaction conditions, high yields (95%), recyclable catalyst potential, and a harmless environmental effect.^84^

**Scheme 20:**
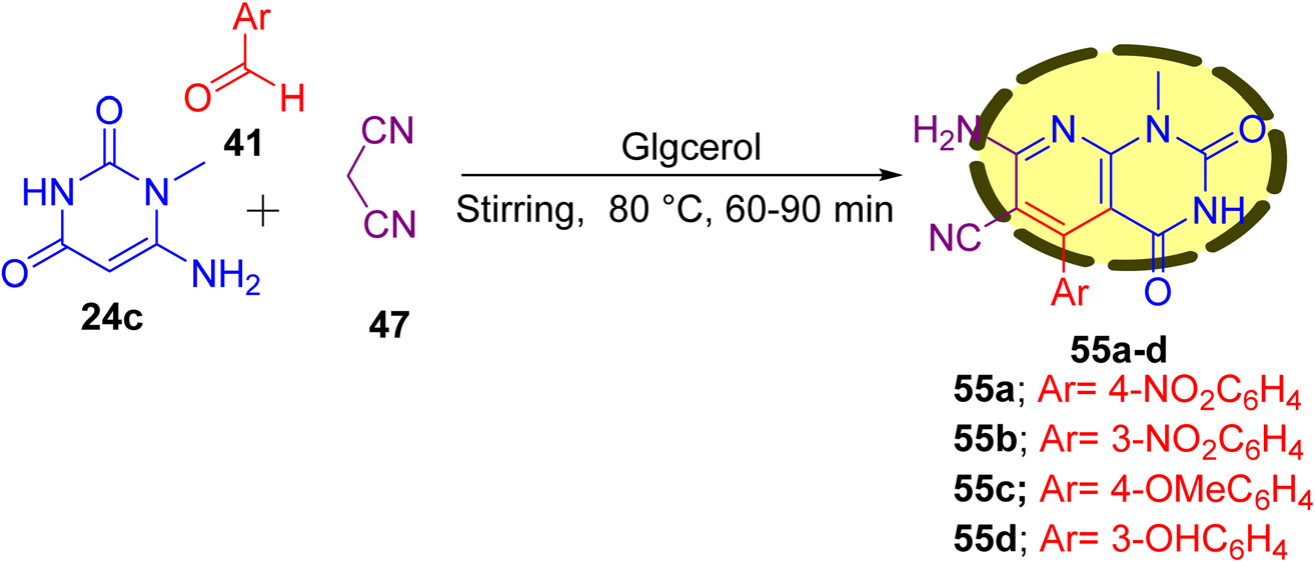
Catalyst-free advancement of fused pyrimidine derivatives 55a–d in glycerol.

The respective one mole of aromatic aldehyde 41 (1 mmol) and one mole of malononitrile (47) (1 mmol) reacted with one mole of the 6-amino-1-methyluracil (24c) (1 mmol) added to the reactant mixture and were mixed to generate 55a–d (Scheme 20) in 5 mL of glycerol under stirring, and the temperature of the reaction was set at 80 °C, a beneficial medium that was reused. The current technique's benefits refer to a one-pot catalyst-free, environmentally friendly strategy, complete economy, low costs, a wide range of substrates, ease of running, quick reaction times, a simple preliminary process, and excellent yields (90–94%).^85^

**Scheme 21:**
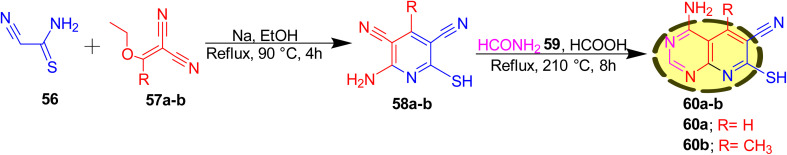
Synthesis of fused pyrimidine derivatives 60a–b.

The reaction of cyanothioacetamide (56) (22.3 mmol) with 2-(arylidene)malononitrile 57a–b (23 mmol) in sodium ethoxide, prepared from Na (570 mg) in absolute ethyl alcohol (50 mL), was heated under reflux at 90 °C for 4 hours and afforded 2-amino-6-mercaptopyridine-3,5-dicarbonitrile 58a–b. The 0.01 mol of the previous product was reacted along with formamide (15 mL) (59), and 5 mL of formic acid was heated under reflux at 210 °C for 8 hours, yielding pyridopyrimidine 60a–b (Scheme 21).^86^

**Scheme 22:**
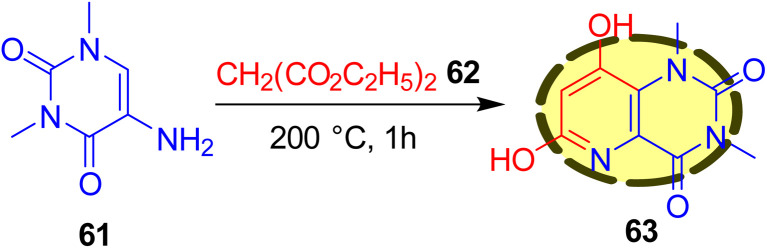
Synthesis of fused bicyclic rings 63.

The fused bicyclic pyrimidine 63 was obtained by heating a combination with 5-amino-l,3-dimethyluracil (61) (23 mmol) and diethyl malonate (62) (23 mmol) at 200 °C (Scheme 22).^87,88^

**Scheme 23:**
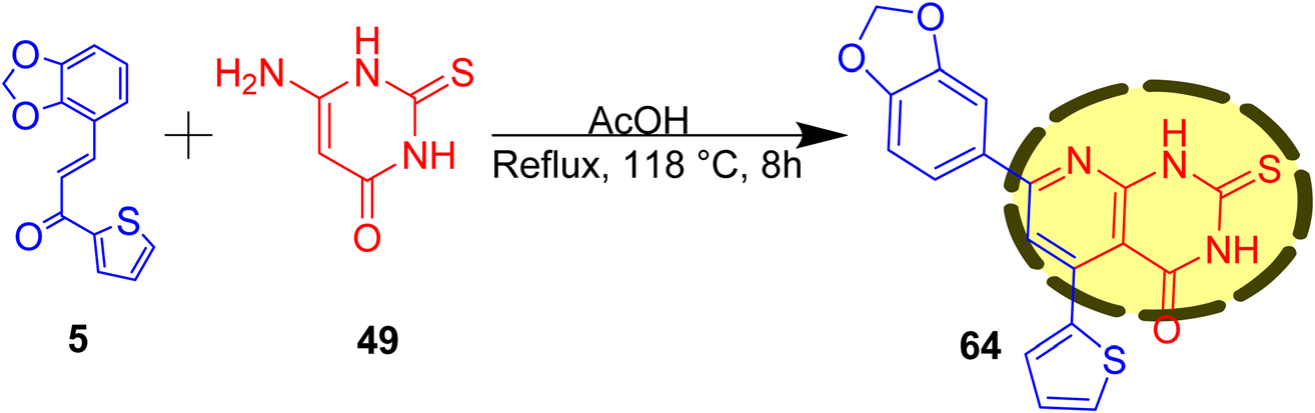
Synthesis of fused pyrimidine hybrid 64.

Chalcone 5 (5 mmol), as the starting substrate, reacted with 6-aminouracil analogue 49 (5 mmol) in glacial acetic acid (20 mL) and was heated at reflux at 118 °C for 8 hours to yield thioxopyrido[2,3-*d*]pyrimidine hybrid 64, as Scheme 23 illustrates.^56^

**Scheme 24:**
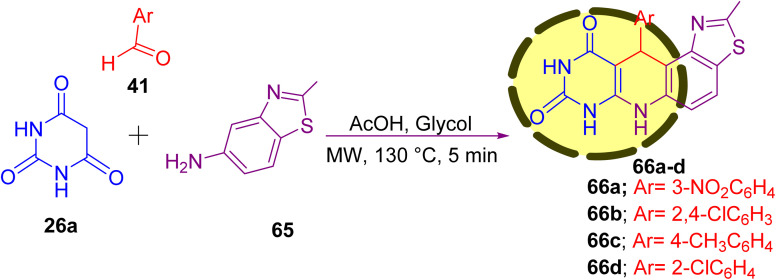
Microwave-assisted advancement of tetra-fused pyrimidine frameworks 66a–d.

Scheme 24 shows how Tu *et al.* used fused thiazole amine 65 (1 mmol), barbituric acid (26a) (1 mmol), and aromatic aldehydes 41 (1 mmol) in a combination of AcOH (1 mL) and ethylene glycol (1 mL) without a catalyst under microwave irradiation at 130 °C to develop a tetra-fused ring framework 66a–d. High yields (90–93%) and a simple conditioning process with a quick reaction time (5 minutes) are the benefits of this one-pot approach.^89,90^

**Scheme 25:**
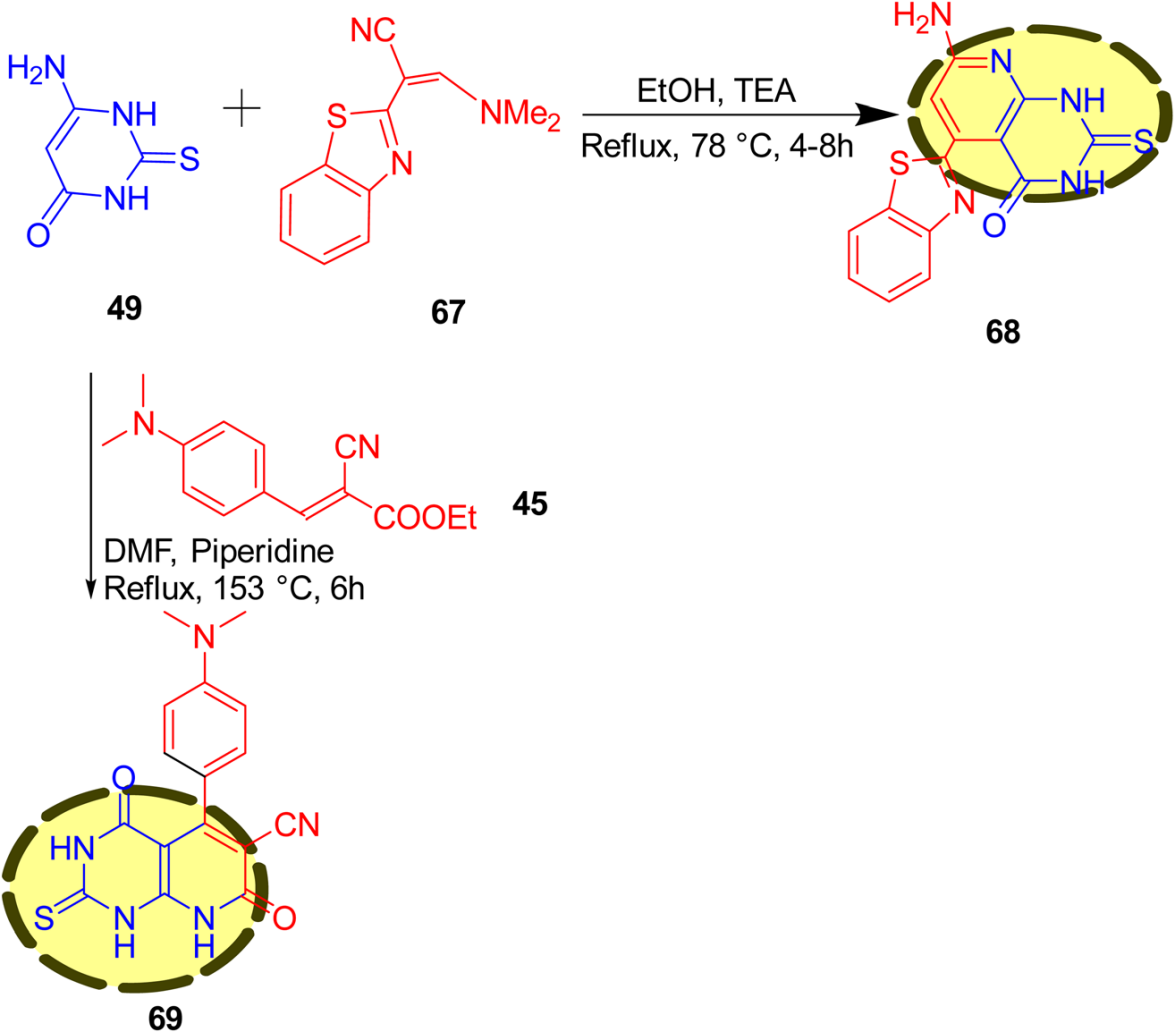
Chemical synthesis procedure for fused N-heterocycle 68–69.

Furthermore, compound 49 (10 mmol) was treated using fused thiazolo-dimethylamino-acrylonitrile 67 (12 mmol) in refluxing ethanol (20 mL) with a catalytic amount of TEA (5 drops) at 78 °C for 4–8 hours to produce pyridopyrimidinone 68 (Scheme 25). Additionally, component 49 (10 mmol) might be refluxed at 153 °C for 6 h utilizing benzylidene acetonitrile derivative 45 (12 mmol) within DMF (20 mL) having a catalytic quantity of piperidine to be a base to produce fused N-heterocycle-based pyrimidine 69.^91^

**Scheme 26:**
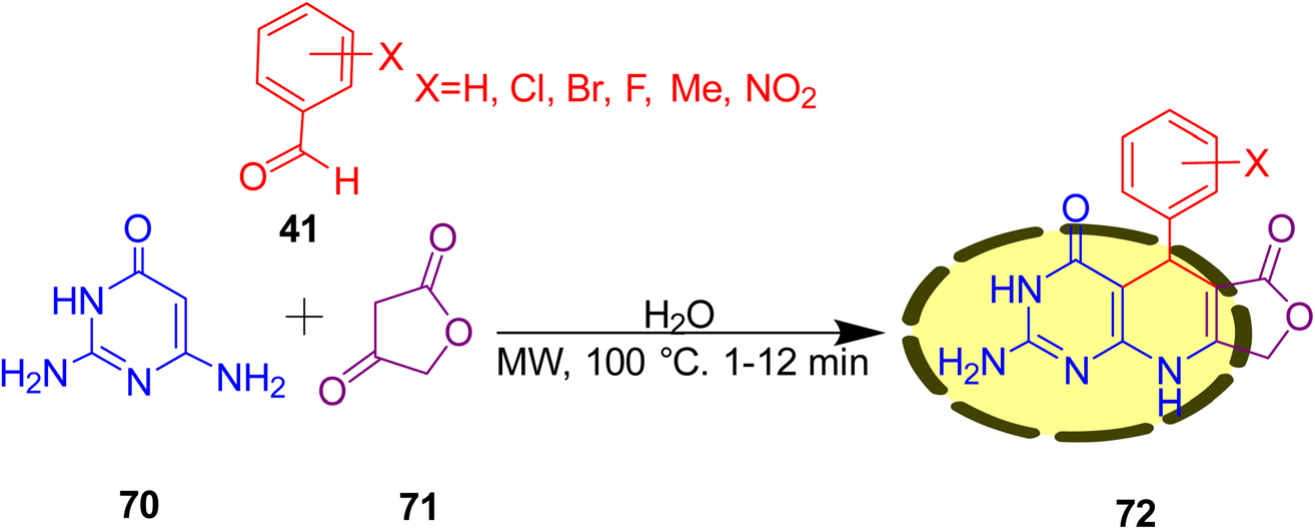
Catalyst-free development of fused tricyclic pyrimidine hybrids 72 in water.

In Scheme 26, a series of fused tricyclic pyrimidine hybrids 72 were obtained by a catalyst-free interaction between aminopyrimidine scaffold 70 (1 mmol), aldehyde 41 (1 mmol), and indane-1,3-dione (71) (1 mmol) in water (2 mL). The mixture was irradiated at 150 W and at 100 °C for 1–12 minutes. To achieve environmentally friendly, microwave (MW) and ultrasound (US) facilitated organic synthesis in water, there is currently a lot of focus. MW and US technologies are effective in the ensuing heating process that initiates the majority of chemical processes, according to investigators in a published report. Additionally, these devices provide energy in accordance with the developers' objective. In other instances, controlled MW dielectric heating has been shown to significantly shorten reaction times—from days to hours, occasionally even minutes. Because of these advantages, scientists have been motivated to use the more environmentally friendly methods of MW and US in the manufacturing of heterocyclic molecules.^92^

**Scheme 27:**
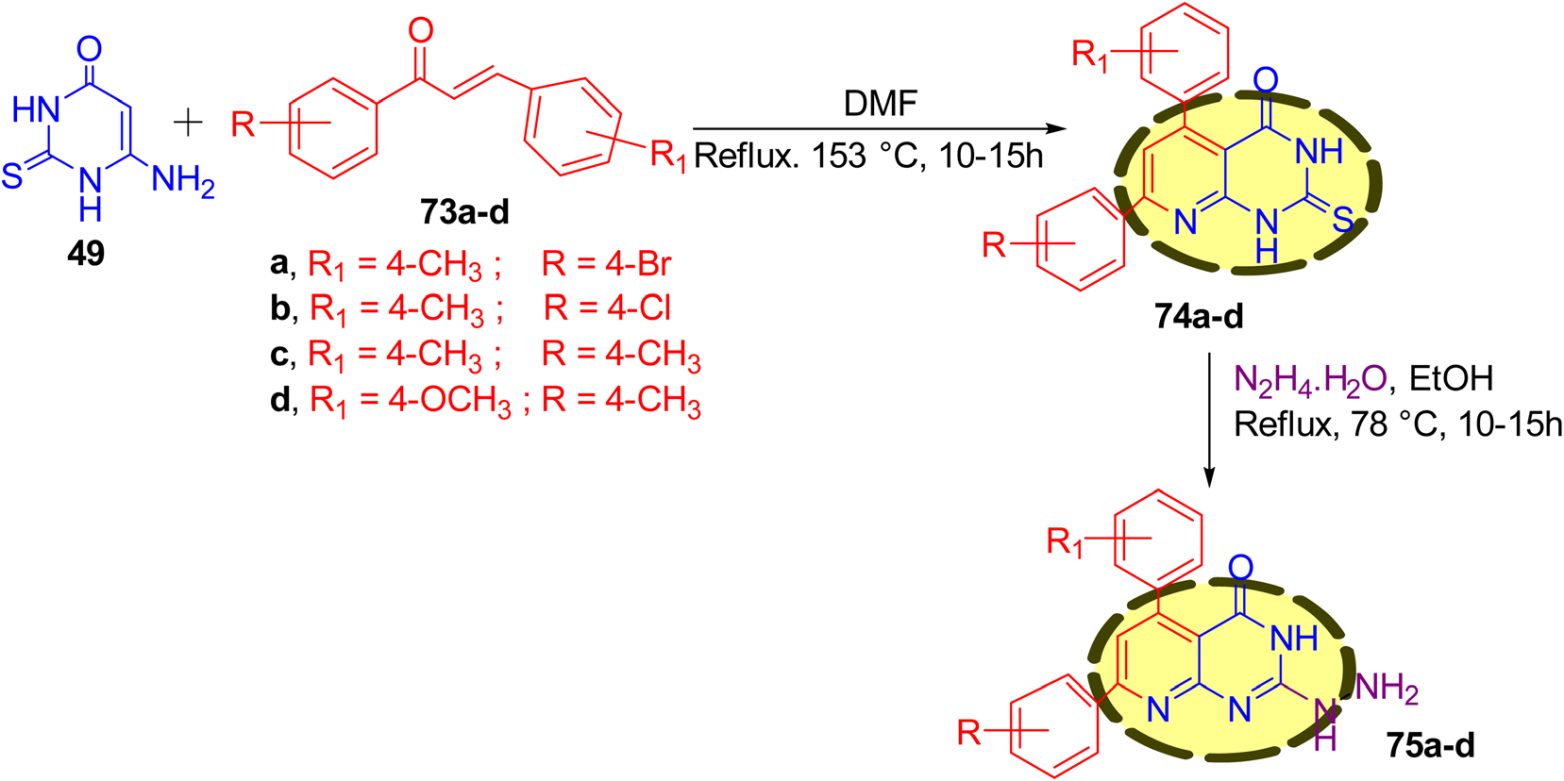
Chemical development procedure for fused pyrimidine hybrids 74a–d, 75a–d.

5,7-Diarylpyrido[2,3-*d*]pyrimidinones 74a–d were produced as a result of the reflux of 49 (0.01 mol) at 153 °C for 10–15 hours using DMF (dry) (20 mL) containing α,β-unsaturated ketones 73a–d (0.01 mol) (Scheme 27). Hydrazine reagent 99% (0.006 mol) and derivatives 74a–d (0.004 mol) were refluxed at 78 °C in 20 mL of absolute EtOH for ten to fifteen hours.^93^

**Scheme 28:**
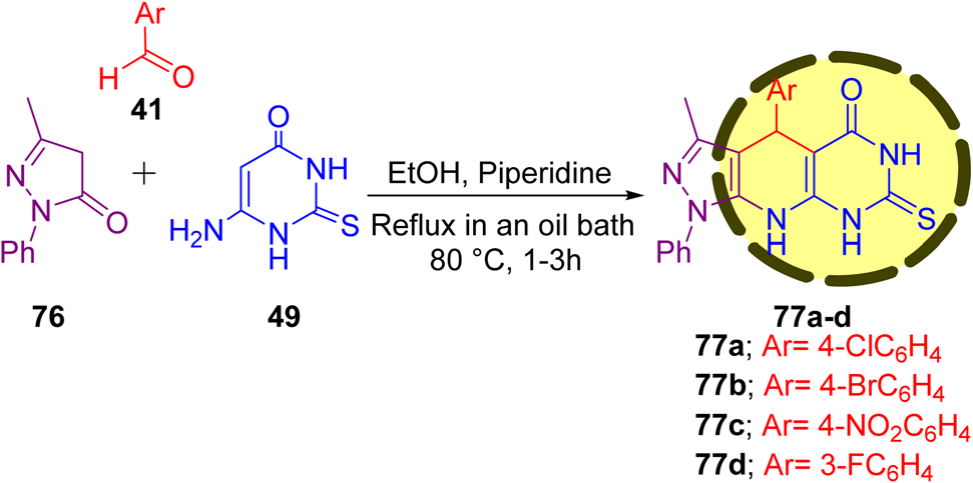
Syn. of tricyclic pyrimidine hybrids 77a–d.

A one-pot process involving 6-aminothiouracil (49) (1.0 mmol) with aldehydes 41 (1.0 mmol) and pyrazoline-5-one 76 (1.0 mmol) in EtOH (8 mL) at reflux in an oil bath maintained at 80 °C for 1–3 hours produces several components of tricyclic pyrimidine hybrids 77a–d functioning in proper quantities (72–96%) when piperidine is present as a basic catalyst (Scheme 28).^94,95^

**Scheme 29:**
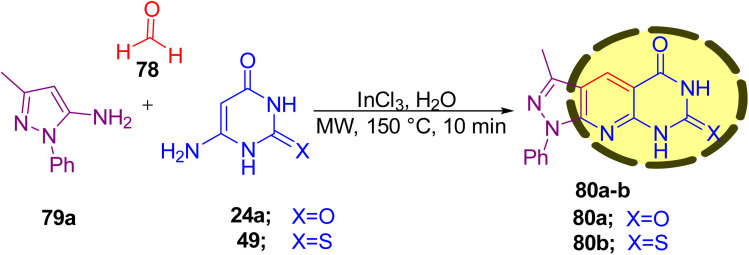
Development of tricyclic pyrimidine hybrids 80a–b.

In the corporation of InCl_3_ (15% mol) and water (3 mL), formaldehyde (78) (1 mmol), pyrazole-5-amine 79a (1 mmol), and 6-aminouracil (24a) or 6-aminothiouracil (49) (1 mmol) were irradiated under MW at 150 °C for 10 min to make tricyclic pyrimidine hybrids 80a–b indicated in Scheme 29.^96^ It has been discovered that this method works well for producing innovative N-fused heterocycle molecules in high yields (95% and 91%, respectively).^97^ These reaction conditions and product yields provide a desirable comparison for the development methods of the previous scheme.

**Scheme 30:**
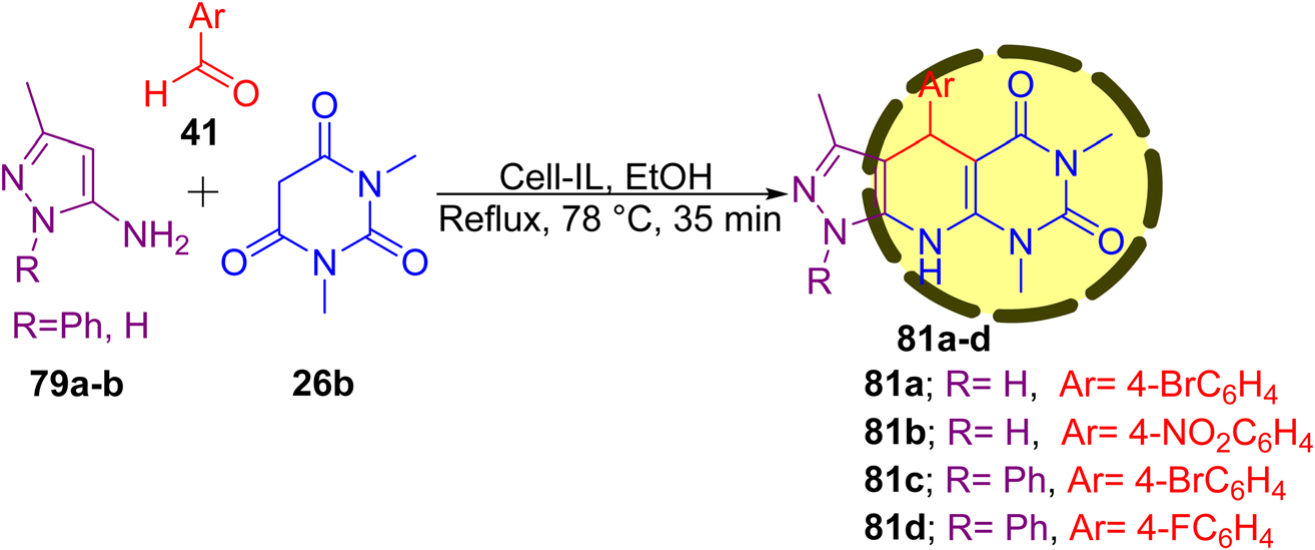
Cellulose-supported ionic liquid catalysis for fused pyrimidinedione synthesis 81a–d.

Stasia *et al.* apparently polycyclic N-heterocycle-based pyrimidine moieties 81a–d can be obtained using aminopyrazoles 79a–b (1 mmol), aldehydes 41 (1 mmol), 1,3-dimethylbarbituric acid (26b) (1 mmol), and 5 mL of ethanol heated in the presence of 30 mg of Cell-IL (Scheme 30) during reflux at 78 °C for 35 minutes in the corporation. It has been discovered that Cellulose-supported acidic ionic liquid (Cell-IL), a novel kind of biopolymer-based heterogeneous catalyst, is exceedingly efficient for the region-specific production of fused pyrimidinediones 81a–d.^98,99^

**Scheme 31:**
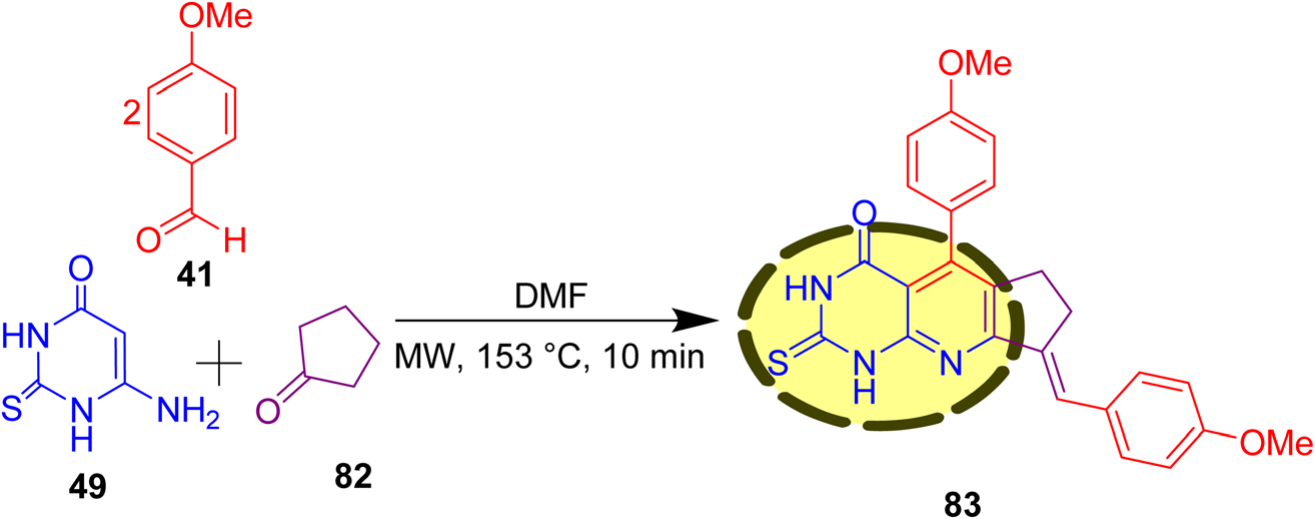
Pyrido[2,3-*d*]pyrimidin-4-one 83 syn. *via* cyclocondensation with microwave enhancement.

A cyclocondensation process between one equivalent of each of cyclopentanone (82) (0.02 mol) and 6-aminothiouracil (49) (0.02 mol) and two equivalents of *p*-anisaldehyde (41) (0.04 mol) in DMF (10 mL) were set in a Teflon vessel and exposed to an 800 W microwave at 153 °C for 10 min, yielding pyrido[2,3-*d*]pyrimidin-4-one 83 (Scheme 31).^100,101^ A subfield of green chemistry called microwave-facilitated chemical synthesis development, or “MORE,” has garnered a lot of interest recently due to its quick chemical production. The microwave aims to enhance the speed of reaction while being easy to use, inexpensive, and harmless to the environment.^102^

#### 3.1.3 Synthesis of pyrano[2,3-*d*]pyrimidine analogous

**Scheme 32:**
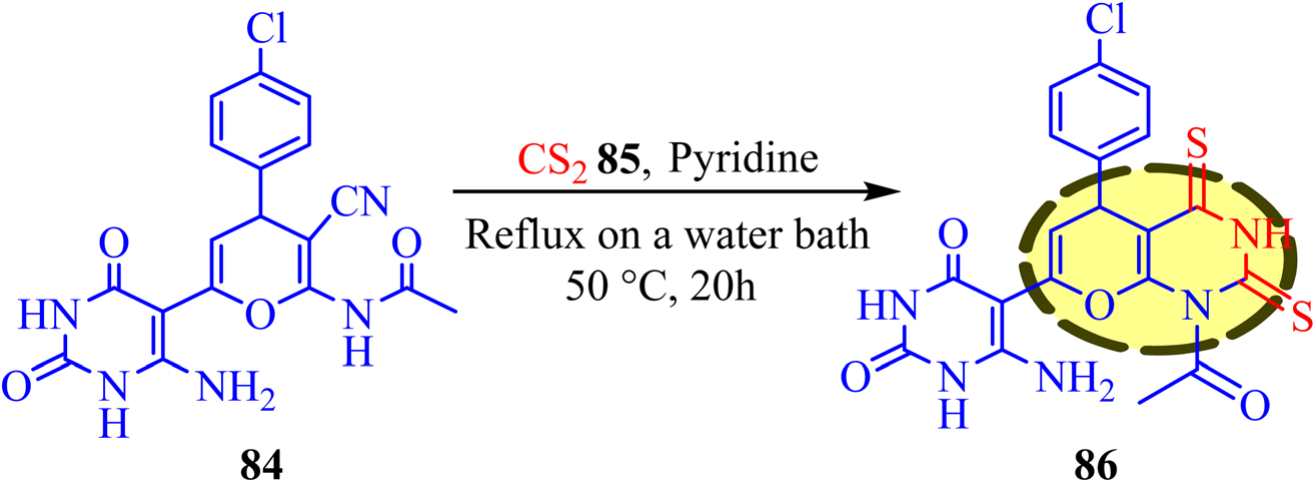
Reaction of 5-(2-pyranyl)uracil 84 with CS_2_85, for the formation of compound 86.

**Scheme 33:**
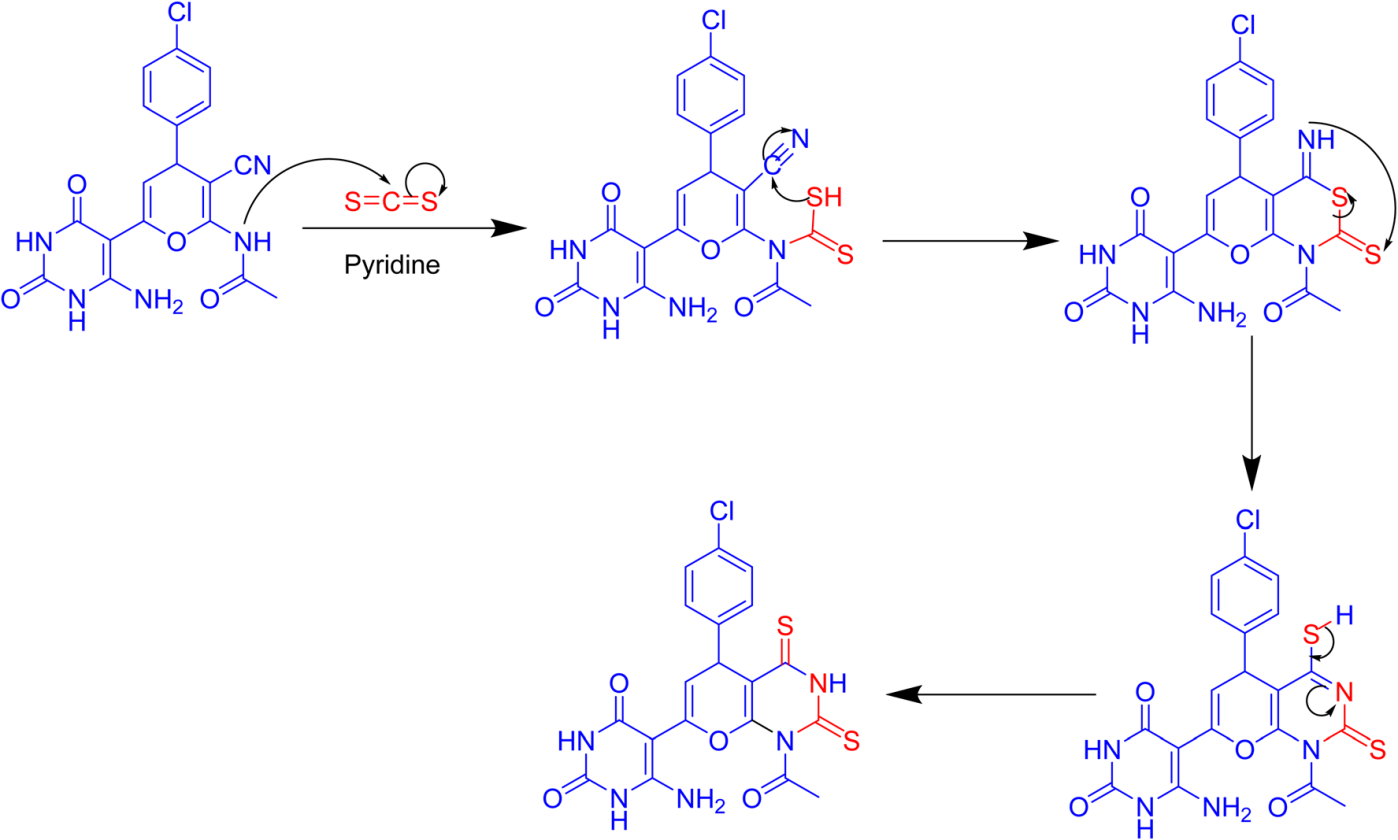
The Dimroth rearrangement is the suggested process for molecule 86's production.

One mole of CS_2_85 was added to a solution of one mole of molecule 84 in pyridine (10 mL), and the mixture was heated under reflux on a water bath at 50 °C for 20 hours. After cooling, the resulting mixture was diluted with EtOH, and pyranopyrimidine 86 was produced *via* Dimroth rearrangement (Scheme 32). The mechanism illustrated in Scheme 33.^14^

**Scheme 34:**
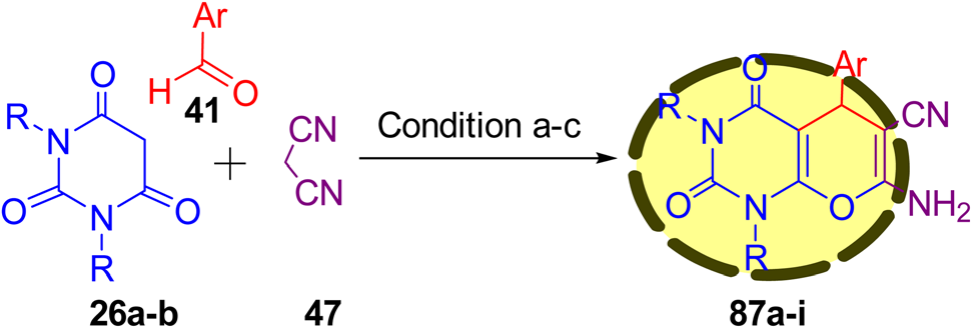
Syn. of fused pyrimidine derivatives 87a–i.

Several fused bicyclic pyrimidine hybrids made under different conditions. An unsaturated N-heterocyclic compound having one oxygen atom at position 8 and two nitrogen atoms at positions 1 and 3, respectively, annulated pyrano[2,3-*d*]pyrimidines are formed *via* the fusion of pyran and pyrimidine rings. A variety of methods based on various multicomponent processes are used to develop annulated pyrano[2,3-*d*]pyrimidines, including Knoevenagel condensation, Michael addition, and cyclodehydration approaches. Refluxing an equimolar proportion of aromatic aldehydes 41 within malononitrile (47) as well as barbituric acid 26a–b, under various conditions (Scheme 34 and Table 2), yielded a number of annulated pyrano[2,3-*d*]pyrimidines 87a–i with a pyrimidine backbone with a high yield (87–97%).^103^

**Table 2:** General requirements for pyranopyrimidine derivative syn

Condition	Condition no.	Substitution		Cpd. no.	Yield	References
		R	Ar			
1,4-Diazabicyclo[2,2,2]octane (DABCO) (5 mole%), EtOH (8 mL), H_2_O (8 mL), r.t	a	H	C_6_H_5_	87a	94%	104 and 105
			4-OCH_3_C_6_H_4_	87b	91%	
			3,4-OCH_3_C_6_H_3_	87c	91%	
			3-OHC_6_H_4_	87d	91%	
			4-BrC_6_H_4_	87e	90%	
Alum (10%), H_2_O (5 mL), 80 °C	b	H	C_6_H_5_	87a	92%	98, 106, and 107
			4-OCH_3_C_6_H_4_	87b	93%	
			4-BrC_6_H_4_	87e	91%	
			3-OHC_6_H_4_	87d	92%	
Catalyst-free, magnetized deionized water (MDW) (5 mL), 70 °C	c	CH_3_	C_6_H_5_	87f	97%	108
			4-OCH_3_C_6_H_4_	87b	92%	
			2-BrC_6_H_4_	87g	87%	
			3-BrC_6_H_4_	87h	91%	
			4-CNC_6_H_4_	87i	95%	

**Scheme 35:**
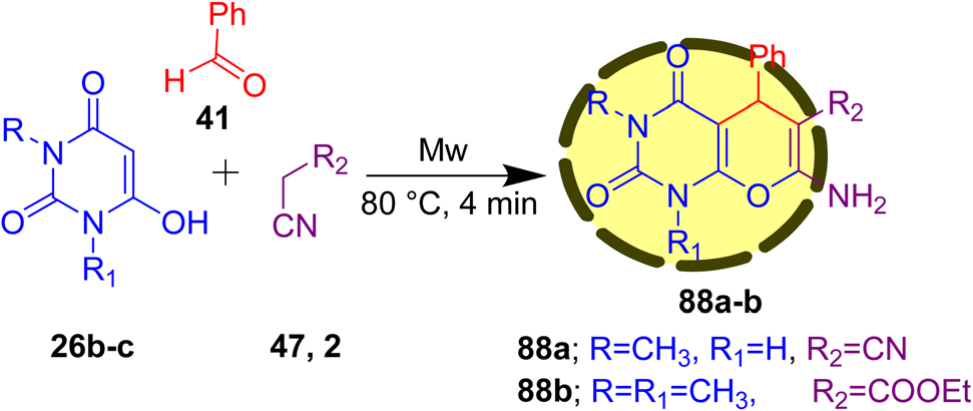
Syn. of pyrano[2,3-*d*]pyrimidines 88a–b in Mw condition.

From the perspective of multifunctional chemistry, multicomponent reactions (MCRs) have garnered significant interest due to their ease of operation, productivity, and typically high yields of molecules.^109,110^ Microwave-assisted three-component cyclocondensation of equimolar amounts of barbituric acid 26b–c, benzaldehyde (41), and alkyl nitriles (47, 2), allowed to react under microwave irradiation at 60% power and 80 °C for 4 min, yielded pyrano[2,3-*d*]pyrimidines 88a–b (Scheme 35).^111^

**Scheme 36:**
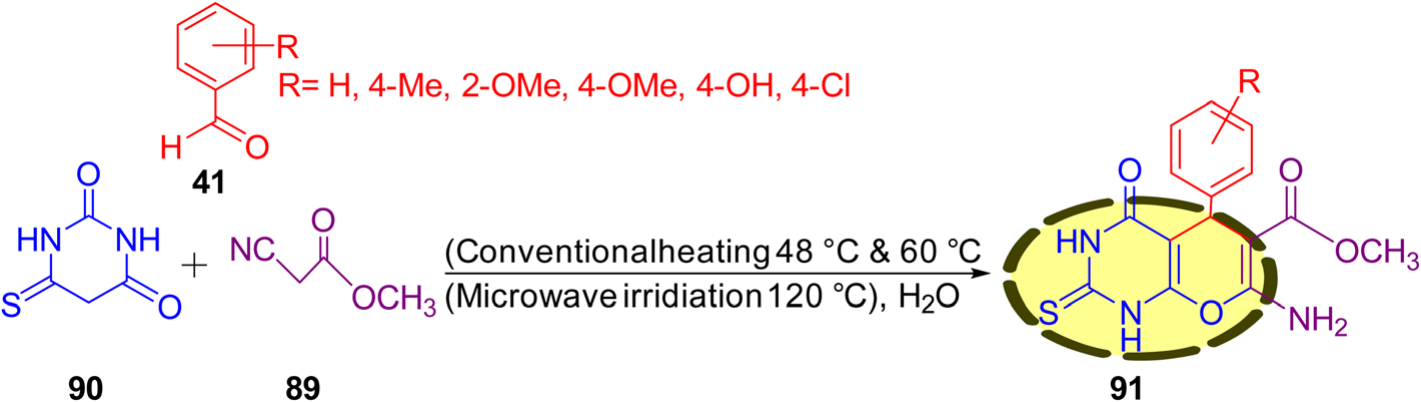
Microwave synthesis of pyrano[2,3-*d*]pyrimidine hybrids 91.

Benzaldehyde derivatives 41 (1 mmol), methyl cyanoacetate (89) (1.2 mmol), thiobarbituric acid (90) (1 mmol), and water (8–10 mL) as a green solvent were mixed together, placed in a Teflon jar, and microwave irradiated under catalyst-free conditions for a specified time at 250 W and 120 °C to produce green pathway synthesis of highly functionalized 7-amino-4-oxo-5-aryl-2-thioxo-pyrano[2,3-*d*]pyrimidine-6-carboxylate 91*via* a one-pot three-component domino Knoevenagel–Michael addition reaction (Scheme 36). Due to its ease of use and many benefits, including high yields (78–94%), quick reaction times (3–6 min), safety, and a green product without the need for a catalyst, a range of modified substances were discovered using this approach.^98,112^

**Scheme 37:**
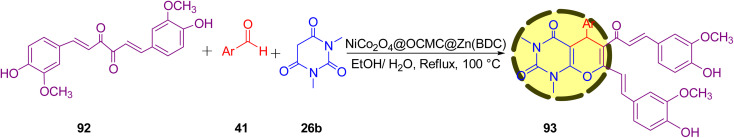
Curcumin-based pyranopyrimidine syn. using a nanocomposite catalyst.

The present study describes an effective production of polycyclic fused pyranopyrimidin 93, which is explained in Scheme 37 through a three-component reaction of curcumin (92) (1.0 mmol), aromatic aldehydes 41 (1.0 mmol), and 1,3-dimethylbarbituric acid (26b) (1.0 mmol) using NiCo_2_O_4_@OCMC(*O*-carboxymethylchitosan)@Zn(BDC) (0.01 g) nanocomposite as a safe and reused catalyst, stirred in ethanol/water (5 mL) under reflux conditions at 100 °C. Curcumin, a class of phenolic chemical analogues derived from the root of *Curcuma longa* (Zingiberaceae), has a number of positive impacts on health and can help prevent particular illnesses.^113^

**Scheme 38:**
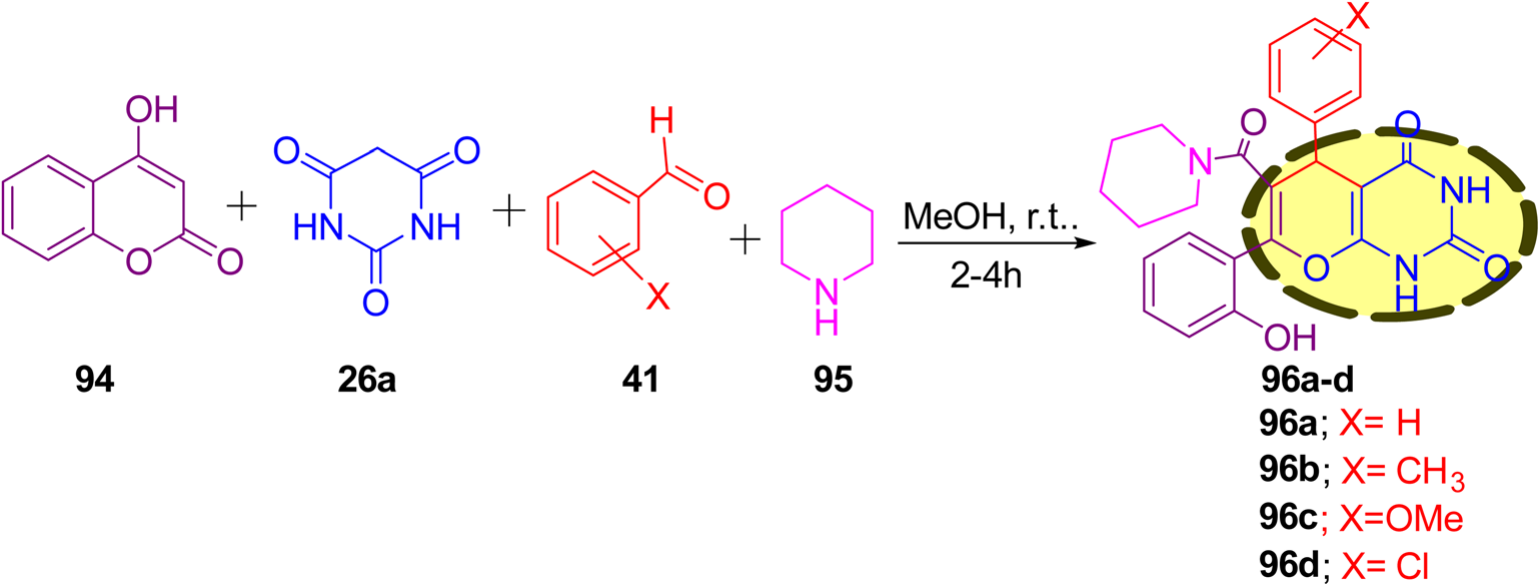
The synthetic strategy used to get the designated products 96a–d.

As shown in Scheme 38, several pyrano[2,3-*d*]pyrimidine-2,4-dione analogues 96a–d were produced by four-component processes incorporating 4-hydroxycoumarin (94) (1 mmol), barbituric acid (26a) (1 mmol), aromatic aldehyde 41 (1 mmol), and piperidine (95) (1 mmol) in methanol (10 mL) magnetically stirred at room temperature for about 2–4 hours.^114^

#### 3.1.4 Synthesis of pyrido[2,3-*d*:5,6-*d*′]dipyrimidine derivatives

**Scheme 39:**
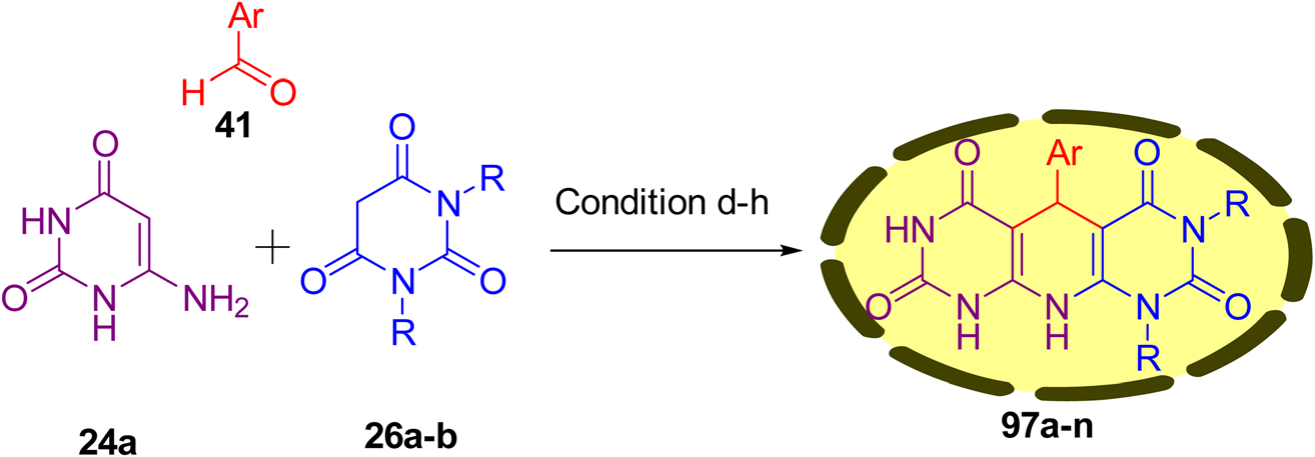
Development of different pyrido[2,3-*d*:5,6-*d*′]dipyrimidine analogues 97a–n.

Using a one-pot three-component condensation Rx. of the analogues barbituric acid 26a–b (1 mmol), 6-aminouracil (24a) (1 mmol), and aromatic aldehydes 41 (1 mmol) reacted under various circumstances under reflux systems to produce pyrido[2,3-*d*:5,6-*d*′]dipyrimidine hybrids 97a–n with different reaction conditions (Scheme 39 and Table 3). These targeted catalysts can be easily removed from the resulting environment with little loss of activity, allowing them to be recovered multiple times.

**Table 3:** General conditions in order to synthesis pyridodipyrimidine

Catalyst	Condition no.	Substitution		Cpd. no.	Yield	References
		R	Ar			
Multi-walled carbon nanotubes (MWCNTs)@l-Histidine (l-His)/Cu(ii) (0.005 g), EtOH (5 mL), reflux, 78 °C	d	H	Ph	97a	92%	115 and 116
			4-OMeC_6_H_4_	97b	89%	
			3-NO_2_C_6_H_4_	97c	93%	
			4-NO_2_C_6_H_4_	97d	93%	
Diisopropylethylammonium acetate (DIPEAc) (5 mL), sir, r.t. 45 min	e	H	Ph	97a	95%	117
			3-ClC_6_H_4_	97e	91%	
			2-OHC_6_H_4_	97f	87%	
			4-OHC_6_H_4_	97g	95%	
			4-OMeC_6_H_4_	97b	91%	
			2,3-(OMe)_2_C_6_H_3_	97h	89%	
Santa Barbara Amorphous (SBA)-Pr-SO_3_H, solvent-free, 140 °C	f	H	Ph	97a	90%	118
			2-OMeC_6_H_4_	97i	85%	
			3-OMeC_6_H_4_	97j	65%	
			4-OMeC_6_H_4_	97b	80%	
			3-NO_2_C_6_H_4_	97c	84%	
			2-OHC_6_H_4_	97f	80%	
			4-OHC_6_H_4_	97g	82%	
			4-NO_2_C_6_H_4_	97d	65%	
			2,3-(OMe)_2_C_6_H_3_	97h	45%	
Piperidine, H_2_O (5 mL), 60 °C, 1h	g	CH_3_	C_6_H_5_	97k	86%	119
			4-MeC_6_H_4_	97l	91%	
			4-NO_2_C_6_H_4_	97m	91%	
			4-ClC_6_H_4_	97n	89%	
MWCNTs@l-His/Cu(ii), EtOH (5 mL), reflux, 78 °C	d	CH_3_	C_6_H_5_	97k	80%	115 and 116
			4-MeC_6_H_4_	97l	78%	
Hexamethylenetetramine-based ionic liquid (HMTA-BAIL)@MIL-101(Cr) (0.008 g), solvent-free, 80 °C	h	CH_3_	C_6_H_5_	97k	92%	120
			4-MeC_6_H_4_	97l	89%	
			4-NO_2_C_6_H_4_	97m	97%	
			4-ClC_6_H_4_	97n	95%	

**Scheme 40:**
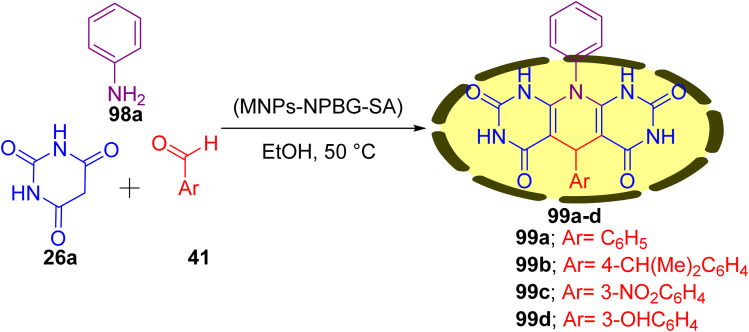
MCR of fused tricyclic pyrimidine hybrids 99a–d using magnetic NPs catalyst.

MNPs-NPBG-SA [magnetic nanoparticles *N*-propylbenzoguanamine sulfonic acid] (7 mg) and 5 mL of EtOH were added to a combination of barbituric acid (26a) (2 mmol), aromatic benzaldehyde 41 (1 mmol), and aniline (98a) (1.2 mmol), and the reaction mixture was stirred magnetically at 50 °C to yield 1,4-dihydropyridine hybrids 99a–d (Scheme 40).^121^

**Scheme 41:**
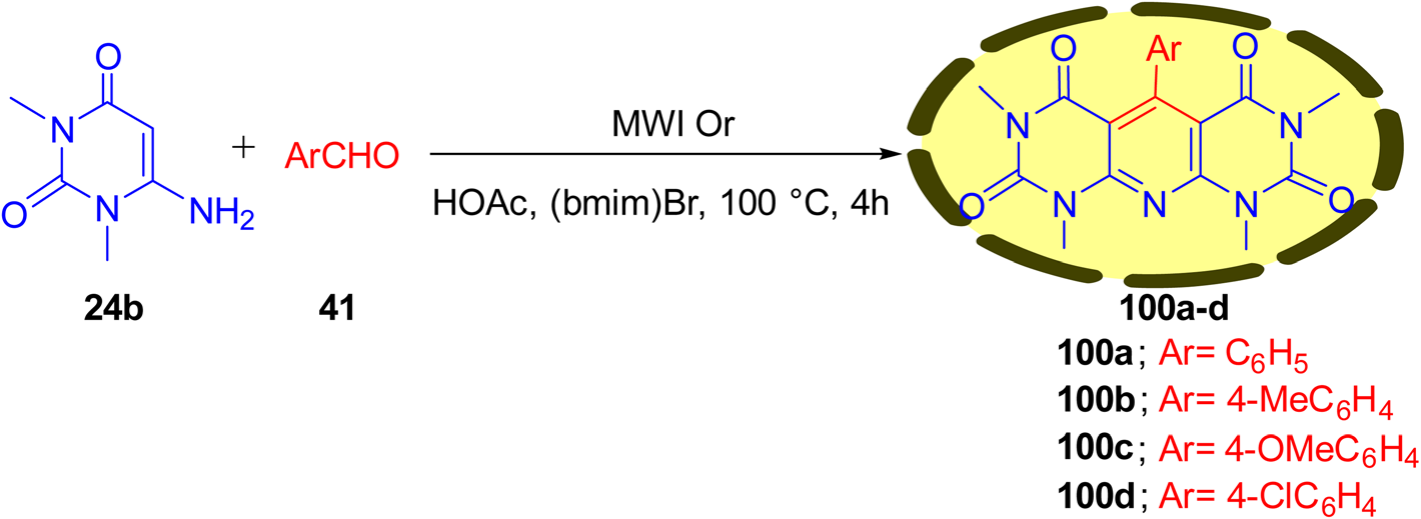
Rx. of aminouracil 24b with aromatic aldehydes 41.

According to Dabiri *et al.*, aromatic aldehyde 41(1 mmol) interacted with two moles of 6-amino-1,3-dimethyluracil (24b) (2 mmol) to produce pyrido[2,3-*d*:6,5-*d*′]dipyrimidine-2,4,6,8-tetrone hybrids 100a–d, as indicated in Scheme 41. Catalyzed by glacial acetic acid (15 mL) in ionic liquid as a solvent, was stirred at 100 °C in a pre-heated oil bath for 4 hours.^122–124^

**Scheme 42:**
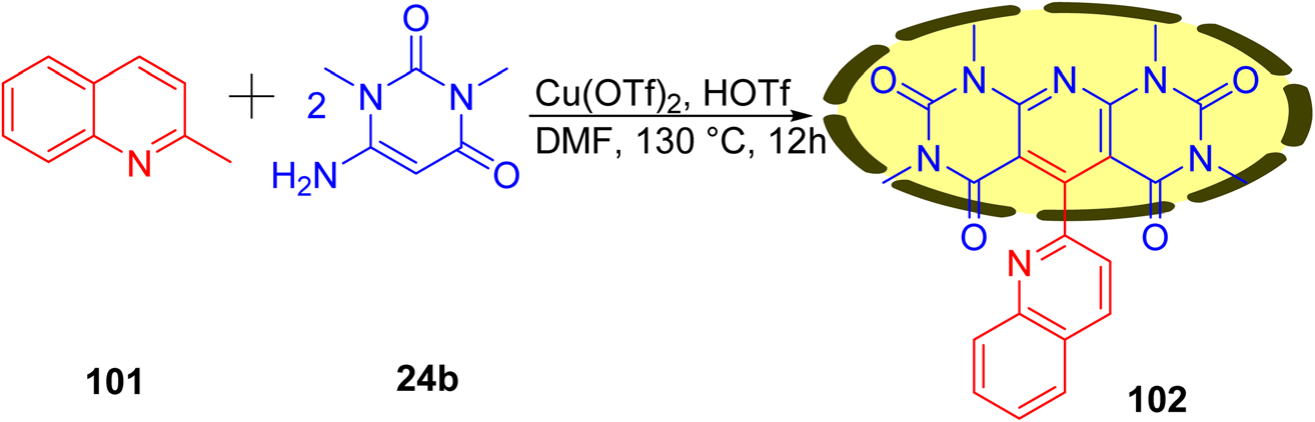
Synthesis of dipyrimidine-fused pyridine 102.

Model substrates for the reaction of 2-methylquinoline (101) (0.3 mmol) and two moles of 6-amino-1,3-dimethylpyrimidine-2,4-dione (24b) (0.66 mmol) with Cu(OTf)_2_ (20 mol%) and additive (1.0 equiv.) in DMF (3.0 mL) heated at 130 °C in an O_2_ environment for 12 hours afforded the desired product 102 outlined in Scheme 42. In addition, O_2_ has been widely used as a natural, environmentally friendly oxidant in the production of organic.^125,126^ Compounds Friedel–Crafts alkylation, Michael addition, deamination, and oxidative aromatization are all part of the chemical pathway.^127^

**Scheme 43:**
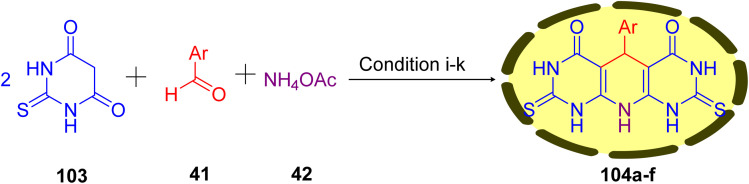
Synthetic route from thiobarbituric acid 103 to dithioxo pyridodipyrimidine conjugates 104a–f.

The synthetic production of pyrido[2,3-*d*:5,6-*d*′]dipyrimidines 104a–f under various circumstances has been described in the present review, with high yields (88–95%). Pyrido[2,3-*d*:5,6-*d*′]dipyrimidines 104a–f were produced from the four-component Rx. of barbituric acid derivative 103 (1 mmol), benzaldehyde analogous 41 (0.5 mmol) and ammonium acetate (42) (0.6 mmol) (Scheme 43 and Table 4).

**Table 4:** General methods in order to synthesis dithioxo pyridodipyrimidine

Condition	Condition no.	Substitution	Cpd. no.	Yield	References
CoFe_2_O_4_@SiO_2_-PA-CC-guanidine-SA (0.06 g), H_2_O (2 mL), r.t	i	Phenyl	104a	95%	128
		4-Nitrophenyl	104b	91%	
		4-Methoxyphenyl	104c	94%	
		3-Methoxyphenyl	104d	88%	
		4-Chlorophenyl	104e	90%	
Nano-[SiO_2_-R-NMe_2_SO_3_H][Cl] (0.02 g), solvent-free, 90 °C	j	Phenyl	104a	89%	129
		4-Nitrophenyl	104b	94%	
		4-Methoxyphenyl	104c	90%	
		3-Methoxyphenyl	104d	88%	
		4-Chlorophenyl	104e	93%	
		2-Flourophenyl	104f	95%	

**Scheme 44:**
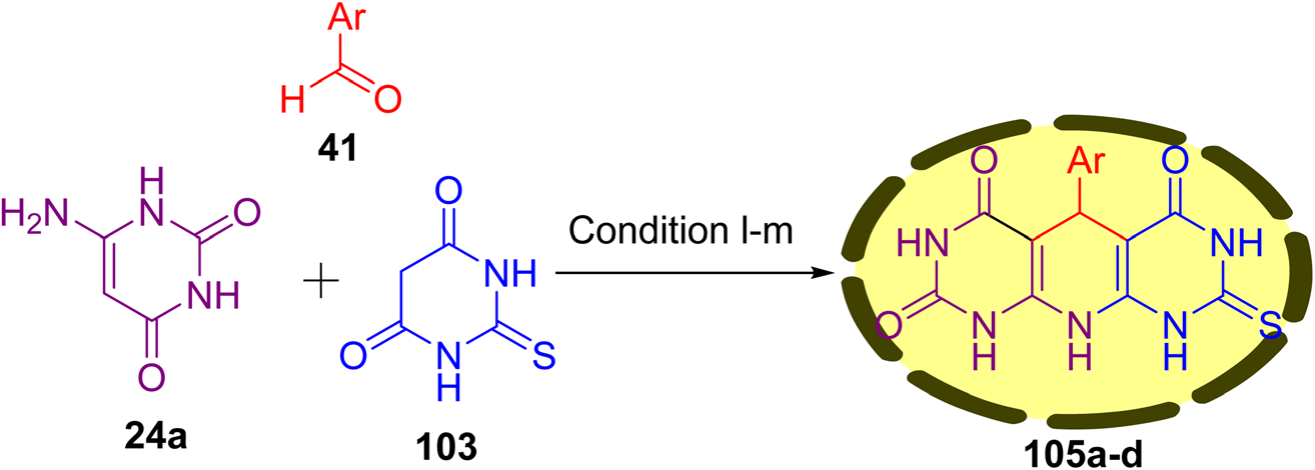
Synthesis of thioxo pyridodipyrimidine conjugates 105a–d.

The development of pyrido[2,3-*d*:5,6-*d*′]dipyrimidines 105a–d under refluxing conditions is reported using a simple three-component one-pot cyclo-condensation process using a barbituric acid analogue (103) (1 mmol), aromatic aldehydes 41 (1 mmol), and 6-aminouracil (24a) (1 mmol) (Scheme 44 and Table 5).

**Table 5:** General conditions in order to synthesis thioxo pyridodipyrimidine

Condition	Condition no.	Substitution	Cpd. no.	Yield	References
DBU (20 mol%), EtOH (14 mL), r.t	l	C_6_H_5_	105a	83%	105 and 130
		4-ClC_6_H_4_	105b	78%	
		4-OMeC_6_H_4_	105c	81%	
		4-OHC_6_H_4_	105d	89%	
*p*-toluene sulfonic acid (*p*-TSA) (0.1 g), H_2_O (5 mL), reflux, 78 °C, 4h	m	C_6_H_5_	105a	81%	131 and 132
		4-ClC_6_H_4_	105b	88%	

**Scheme 45:**
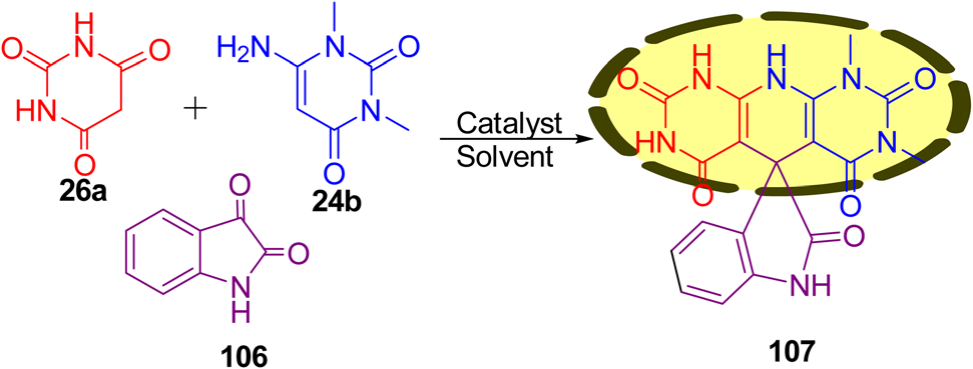
The multicomponent reaction between isatin (106), barbituric acid (26a), and uracil analogous 24b.

According to Scheme 45, a three-component Rx. comprising an analogue of substituted uracil 24b (1 mmol), barbituric acid (26a) (1 mmol), as well as dimedone as a 2,3-dicarbonyl compound 106 (1 mmol) results in the target product 107. 1.5 mol% of catalysts were used. An optimal catalyst for the one-pot advancement of the newly developed spirooxindole derivative 107 in mild circumstances (Table 6) and with a reach sufficient yield was found to be a magnetic, supported, acidic ionic liquid (MSAIL).^133^

**Table 6:** Different condition reaction

Entry	Solvent/condition	Cat.	Time (h)	Yield
1	EtOH (15 mL)/Reflux, 78 °C	—	24	Trace
2	EtOH (15 mL)/Reflux, 78 °C	CH_3_COOH	24	39%
3	MeCN (15 mL)/Reflux, 82 °C	FeCl_3_	24	45%
4	MeCN (15 mL)/Reflux, 82 °C	AlCl_3_	24	50%
5	H_2_O (15 mL)/r.t	Nano-MSAIL	5	89%
6	H_2_O (15 mL)/Reflux, 100 °C	Nano-MSAIL	5	75%
7	DMF (15 mL)/Reflux, 153 °C	Nano-MSAIL	10	75%
8	MeOH (15 mL)/Reflux, 65 °C	Nano-MSAIL	8	75%
9	EtOH (15 mL)/Reflux, 78 °C	Nano-MSAIL	8	75%
10	H_2_O (15 mL)/Reflux, 100 °C	*p*-TSA	13	75%
11	EtOH (15 mL)/Reflux, 78 °C	H_3_PMo_12_O_40_	10	70%
12	H_2_O (15 mL)/Reflux, 100 °C	Nano-Fe_3_O_4_	24	Trace
13	EtOH (15 mL)/Reflux, 78 °C	[MIMPSA][HSO_4_]	12	65%

**Scheme 46:**
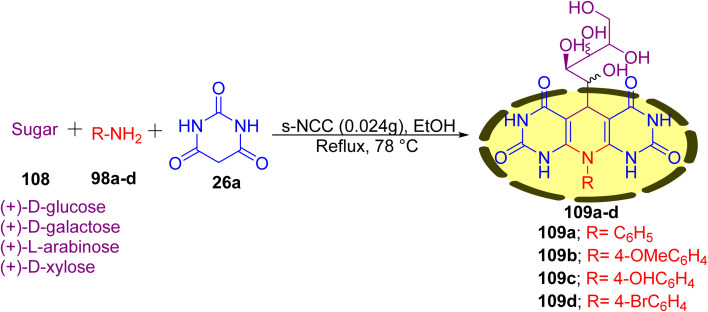
Green syn. of PPFHs 109a–d using s-NCC.

Scheme 46 shows a one-pot, pseudo four-component condensation Rx. of barbituric acid (26a) (2 mmol), carbohydrates 108 (1 mmol), and aromatic amines 98a–d (1 mmol) yielded polyhydroxy pyrimidine-fused heterocyclic compounds (PPFHs) 109a–d using 0.024 g of nanocrystalline cellulose sulfuric acid (s-NCC) in refluxing 78 °C ethanol (5 mL). Mild along with neutral environments, high product yields, quick reaction intervals, ease of process, minimal environmental effects, and simple preparation are only a few benefits of the process. Additionally, the catalysts are recyclable, which aligns with green chemistry.^134^

**Scheme 47:**
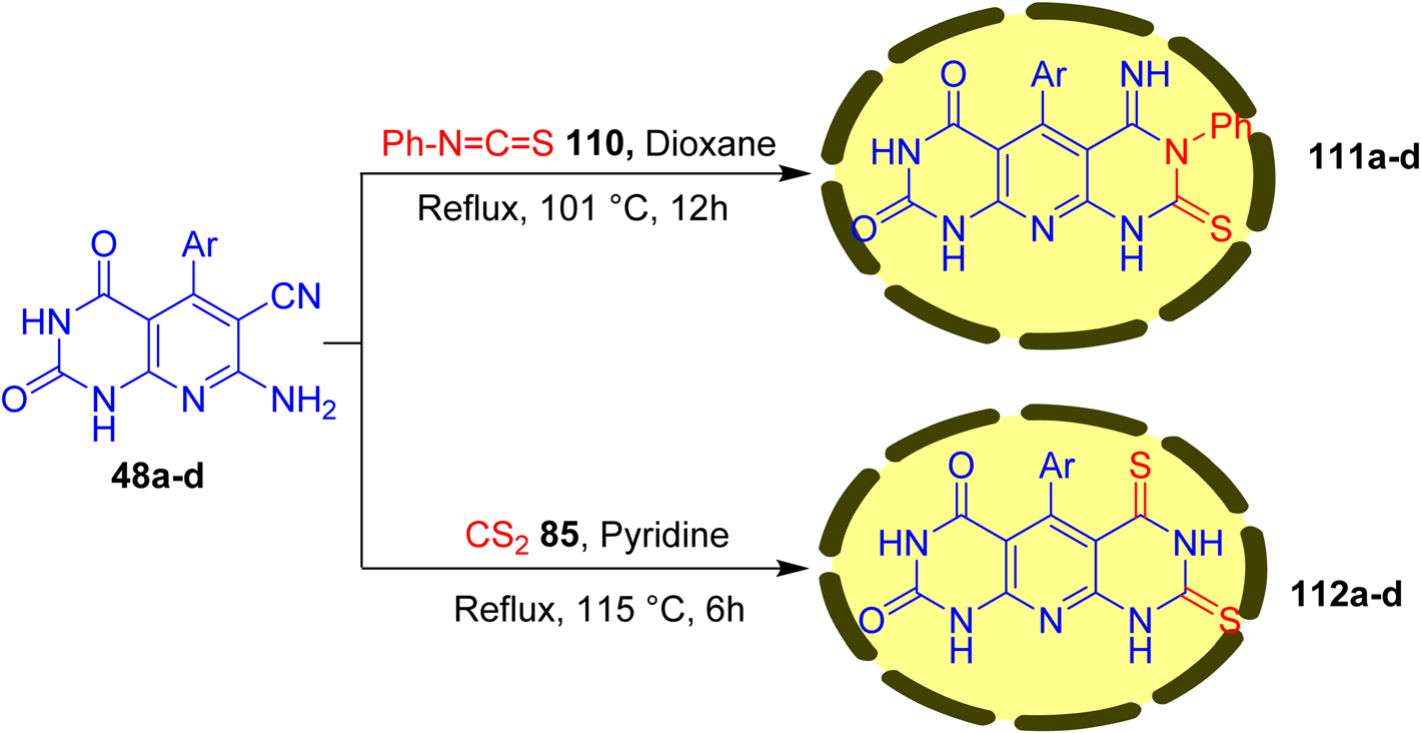
Reactions of compounds 48a–d with phenyl isothiocyanate (110) and carbon disulphide (85).

The synthetic activity of the β-enamino nitrile 48a–d (0.01 mol) was reacted with phenyl isothiocyanate (110) (0.015 mol) and sodium metal (0.01 mol) in dry dioxane (20 mL) and refluxed at 101 °C for 12 hours, or carbon disulphide (85) (0.1 mol) in dry pyridine (30 mL) was heated under reflux at 115 °C for 6 hours to produce pyridopyrimidinethione derivative 111a–d or pyridopyrimidine dithione derivative 112a–d, respectively (Scheme 47).^13^

**Scheme 48:**
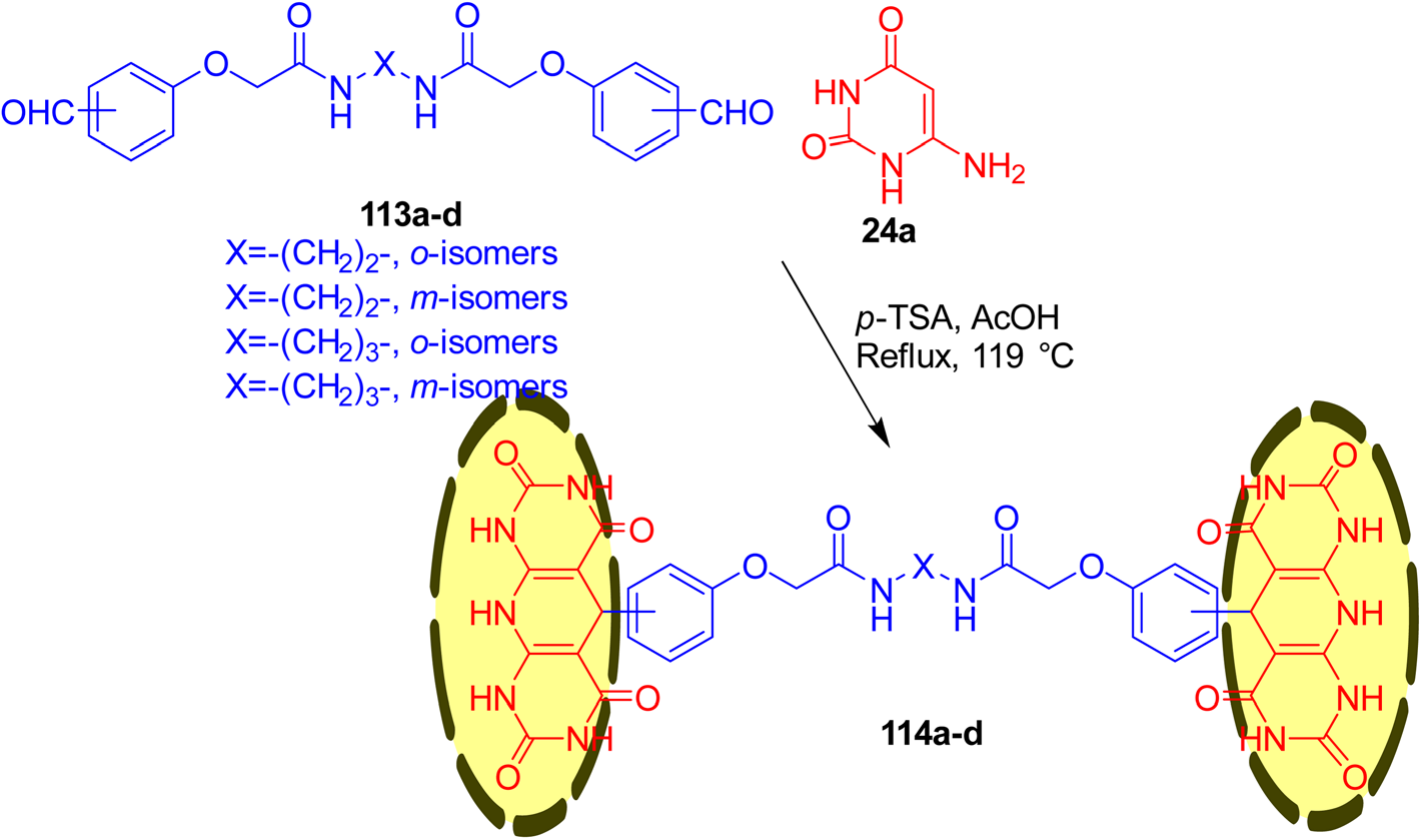
Syn. of bis(pyrido[2,3-*d*:6,5-*d*′]dipyrimidinetetraones) hybrids 114a–d.

The compounds were made in accordance with the literature (Scheme 48). In keeping with our interest in enamine chemistry^135^ and the production of bis(heterocycles), as well as the significance of both 1,4-dihydropyridine and the dipyrimidine ring. Here, we present a highly effective approach for generating bis(pyrido[2,3-*d*:6,5-*d*′]dipyrimidinetetraones) connected to an aliphatic-aromatic core through ester amide connections. A one-pot component cyclo-condensation Rx. of bis(aldehydes) 113a–d with ether-amide bonds and four moles of 6-aminouracil (24a) in boiling AcOH (20 mL) at 119 °C produced bis(pyrido[2,3-d:6,5-*d*′]dipyrimidine-tetraones) 114a–d.^136^

### 3.2 Biological activity of 1,3-dinitrogen atoms heterocyclic and their fused derivatives with their SAR

Pyrimidine derivatives are essential in the search for anticancer drugs because of their superior pharmacological and structural flexibility. Uracil and its derivatives are important chemical substances with a large number of applications. Heterocycles with the uracil moiety show moderate to strong antibacterial activity against a large number of biological targets. The biological targets of changing the substituents on the uracil nucleus range from a variety of cancerous cells, microbial and viral disorders to inflammatory characteristics.

#### 3.2.1 Anticancer activity

Among these, pyrimidine derivatives have garnered particular interest in anticancer drug discovery due to their structural resemblance to nucleobases. Their ability to interact effectively with biological receptors allows modulation of critical signaling pathways involved in tumor proliferation. Academics frequently use 5-fluorouracil and doxorubicin, the most renowned anticancer drugs, as important references in their research.

**Fig. 1:**
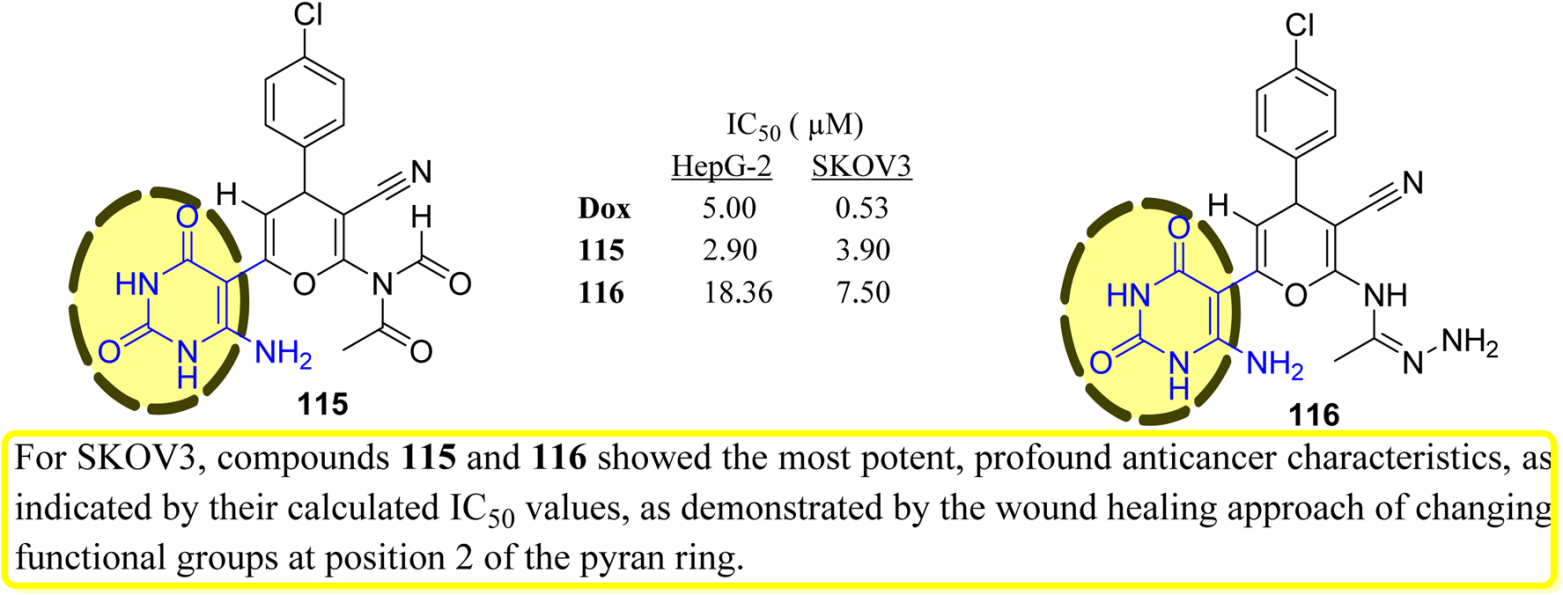
Pyran-functionalized uracil 115–116 containing anticancer activity.

Compound 115 demonstrated the strongest anticancer activity against HepG-2, as evidenced by its computed IC_50_ of 2.90 µM. Compounds 115 and 116 demonstrated the strongest profound anticancer properties for SKOV3, as demonstrated by their computed IC_50_ values of 3.90 µM and 7.50 µM, respectively, in comparison to doxorubicin (Fig. 1).^14^ The SAR investigations demonstrated that, in comparison to the molecular weight of molecules, high molecular weights have remarkably greater cytotoxicity.

**Fig. 2:**
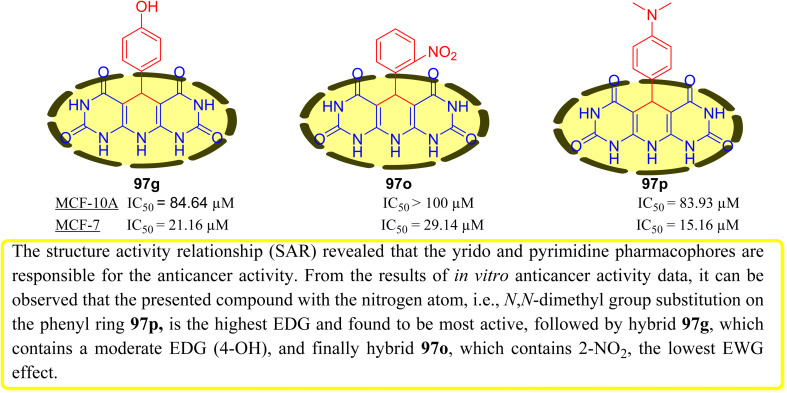
Anticancer compound specificity comparison for breast cell lines of trifused pyrido[2,3-*d*:6,5-*d*′]dipyrimidinetetraone 97g and 97o–p.

To determine the preference for tumor cells, the most active substances, 97g, 97o, and 97p, were evaluated on a breast epithelial cell line that is not tumorigenic (MCF-10A), and adriamycin, a well-known anticancer medication, served as a positive control. Compared to MCF-10A cell lines, molecule 97g demonstrated over fourfold greater sensitivity for tumor cell lines MCF-7. Molecule 97p, the most effective anticancer substance, demonstrated nearly 5.5-fold more specificity for MCF-7 tumor cell lines compared to MCF-10A, indicating that the synthesized substance 97o will not harm or destroy normal cells when employed as a cancer treatment in the future. Substance 97p's sensitivity for tumor cells further indicates that the resulting drug will be less hazardous and have fewer side effects when administered to patients according to the anticancer investigation Fig. 2.^117^

**Fig. 3:**
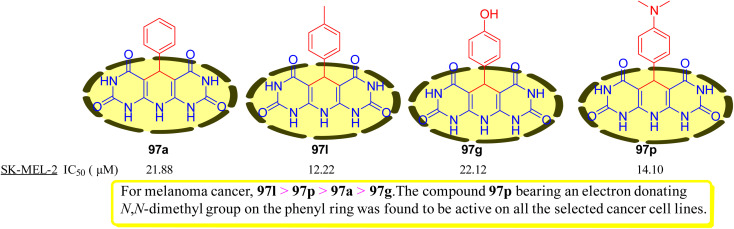
Pyridodipyrimidine moieties 97a, 97g, 97l, and 97p and their antiproliferative activity.

The chemicals 97a, 97g, 97l, and 97p were shown to be potent cancer treatments against the SK-MEL-2 tumor cell line when compared to the conventional medication adriamycin. Substance 97p (IC_50_ = 14.10 µM) has a “nitrogen” atom, or *N*,*N*-dimethyl group, in the para position of the phenyl core. It was satisfying to find that a substance, 97g, which has a hydroxyl group on the phenyl ring at the para position, has an IC_50_ value of 22.12 µM for the SK-MEL-2 tumor cell line. Substance 97l, which has a para-methyl group on the phenyl ring, was shown to be the most effective against SK-MEL-2 tumor cell lines, with an IC_50_ value of 12.22 µM. Substance 97a, which has no phenyl ring substitution, exhibits an IC_50_ value of 21.88 µM for the SK-MEL-2 tumor cell line (Fig. 3).^117^ The SAR investigations demonstrated that electron-donating groups enhance anticancer activity, while polar substituents (97g) weaken it due to increased polarity and reduced lipophilicity. The methyl group, small electron-donating group, improves lipophilicity and enhances cell penetration, thus, hybrid 97l has the most potent cancer activity. The dimethylamino group, strong electron-donating substituent, increases polarity and hydrogen-bonding potential, so hybrid 97p has high cancer action.

**Fig. 4:**
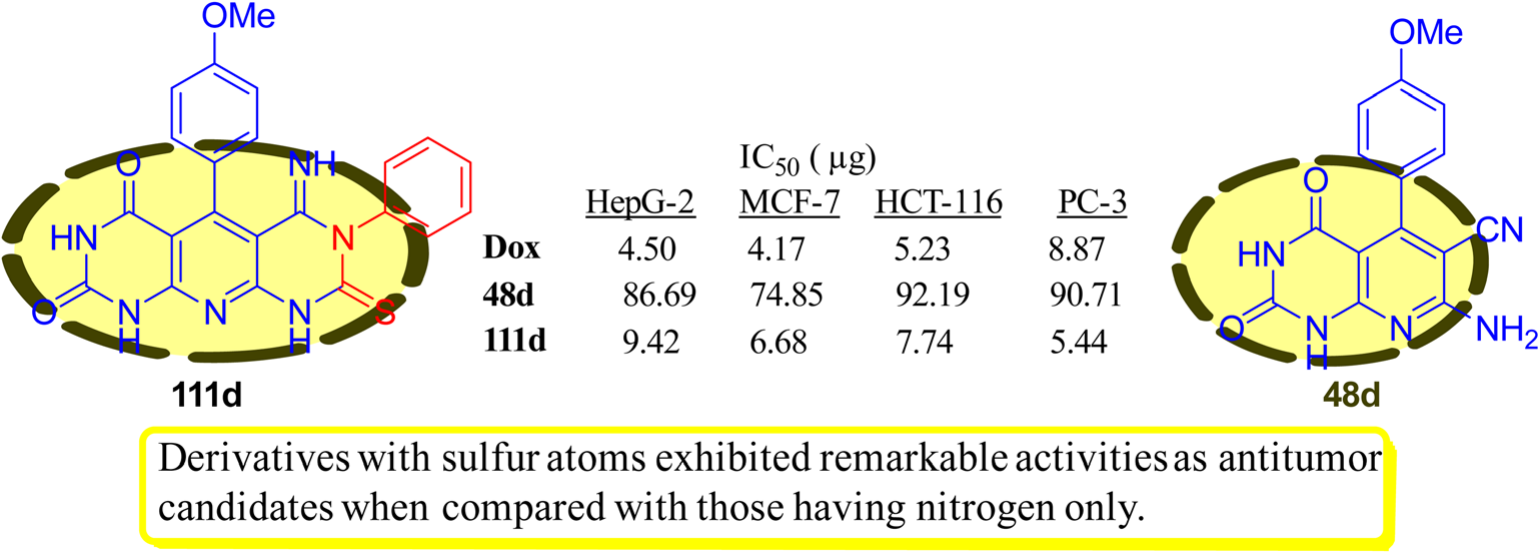
Compounds of pyrido[2,3-*d*]pyrimidine-4(1*H*) dione 48d and 111d that could be *in vitro* anticancer.

Compound 111d showed very strong anti-proliferative activity against the four cell lines HePG-2, HCT-116, PC-3, and MCF-7, with IC_50_ of 9.42, 7.74, 5.44, and 6.68 µg, respectively, which are compared to the reference anticancer medication DOX, which has IC_50_ of 4.50, 5.23, 8.87, and 4.17 µg (Fig. 4).^13^ A sulfur atom (111d) was necessary for potent action more than a found nitrogen atom only (48d), according to the SAR investigation.

**Fig. 5:**
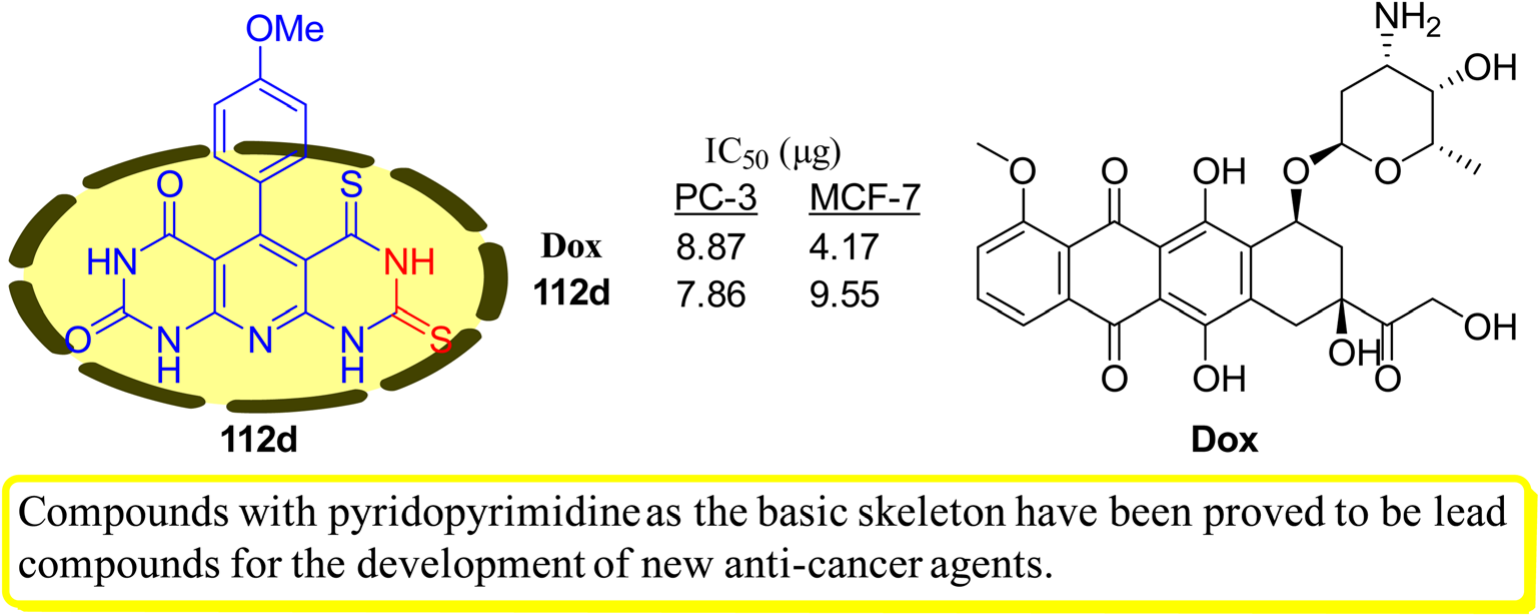
Hybrid 112d with its antiproliferative properties.

Compound 112d showed a much stronger antitumor impact on PC-3 cell lines than the standard reference drug (Dox).^13^ Hybrid 112d demonstrated an antitumor profile, especially toward prostate cancer (Fig. 5). According to SAR research, pyrido[2,3-*d*]pyrimidine-4(1*H*) dione derivatives with a pyrimidine-based heterocyclic ring exhibited stronger anticancer properties than those with other non-heterocyclic systems.

**Fig. 6:**
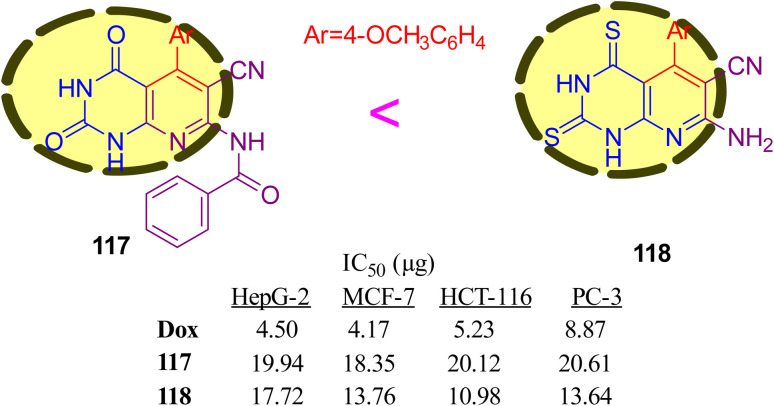
Pyridopyrimidine carbonitrile hybrids 117–118 with its promising antiproliferative character.

With IC_50_ values of 19.94, 20.12, and 18.35 µg, compound 117 showed high anticancer properties against HePG-2, HCT-116, and MCF-7, respectively. IC_50_ values of 4.50, 5.23, 8.87, and 4.17 µg are equivalent to the reference anticancer medication DOX. Compound 118, on the other hand, had antitumor action that is effective against all four cell lines with IC_50_ values of 17.72, 10.98, 13.64, and 13.76 µg for HePG-2, HCT-116, PC-3, and MCF-7, respectively, according to antiproliferative studies Fig. 6.^13^ Sulfur atoms (118) were vital for potent activity more than nitrogen atoms only (117), according to the SAR investigation.

**Fig. 7:**
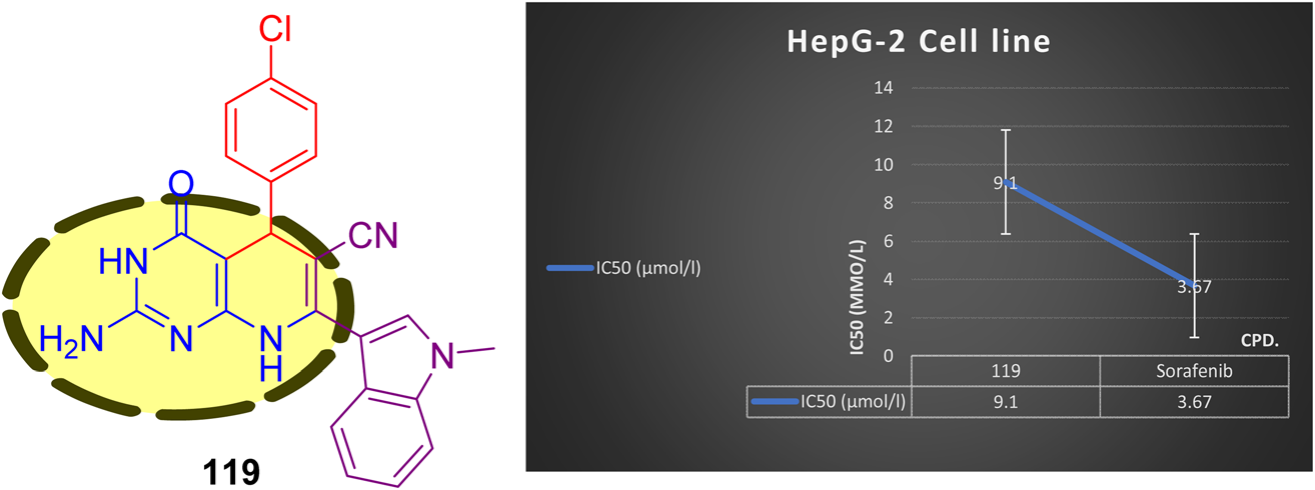
Pyrido[2,3-*d*]pyrimidine 119 with its HepG-2 cell line.

Sorafenib is utilised as the reference medication. Furthermore, derivative 119 was discovered to significantly inhibit the HepG-2 cell line (9.1 µmo L^−1^). Additionally, substance 119 demonstrated anticancer activity by focusing on the ERK1/2 and PI3K/AKT signaling pathways (Fig. 7).^131,137^ According to SAR investigations, activities involved the pyrimidine with a fused system.

**Fig. 8:**
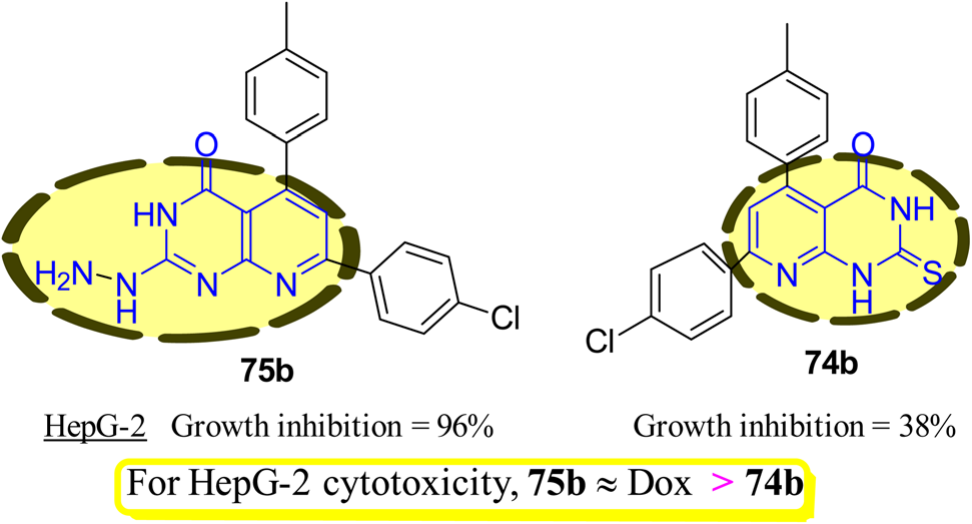
Pyrido[2,3-*d*]pyrimidine hybrids 74b and 75b with antihepatic cancer properties.

The conversion of the 74b's thio group to 75b's hydrazide moiety (equipotent in activity to doxorubicin) significantly increased the antiherpetic cancer action (Fig. 8). The growth inhibition value of doxorubicin is 100%.^93^ The hydrazono moiety is crucial for pyrido[2,3-*d*]pyrimidine' anticancer action, according to SAR studies.

**Fig. 9:**
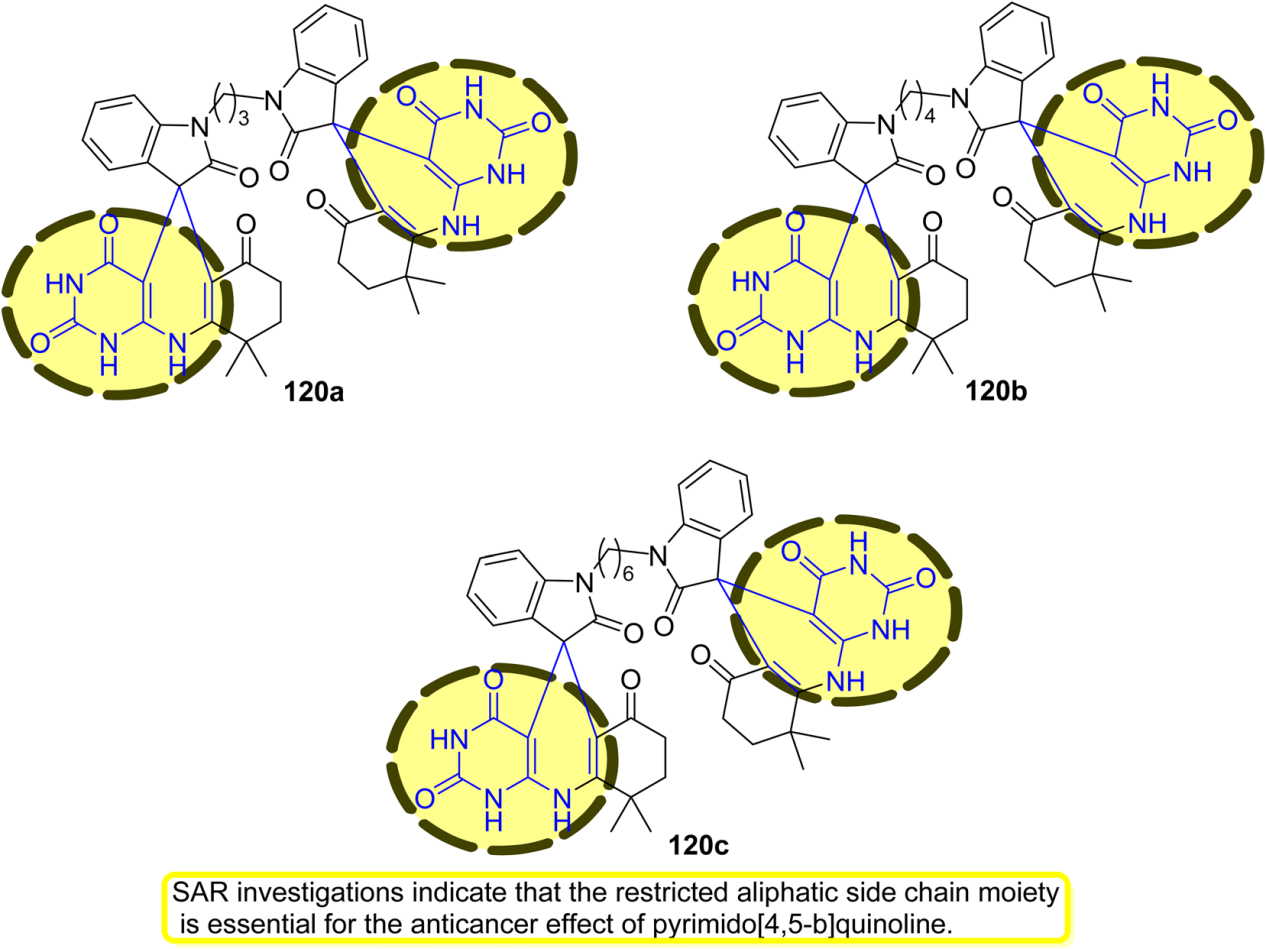
Pyrimido[4,5-*b*]quinoline hybrids 120a–c with anticancer properties.

One popular antibiotic is fluorouracil (5-FU). Among the derivatives 120a–c tested against MCF-7, HCT-116, and A-549 cell lines, substance 120a was shown to be the most active and promising (Fig. 9).^94,138^ The limited aliphatic side chain moiety is crucial for pyrimido[4,5-*b*]quinoline's anticancer action, according to SAR studies.

**Fig. 10:**
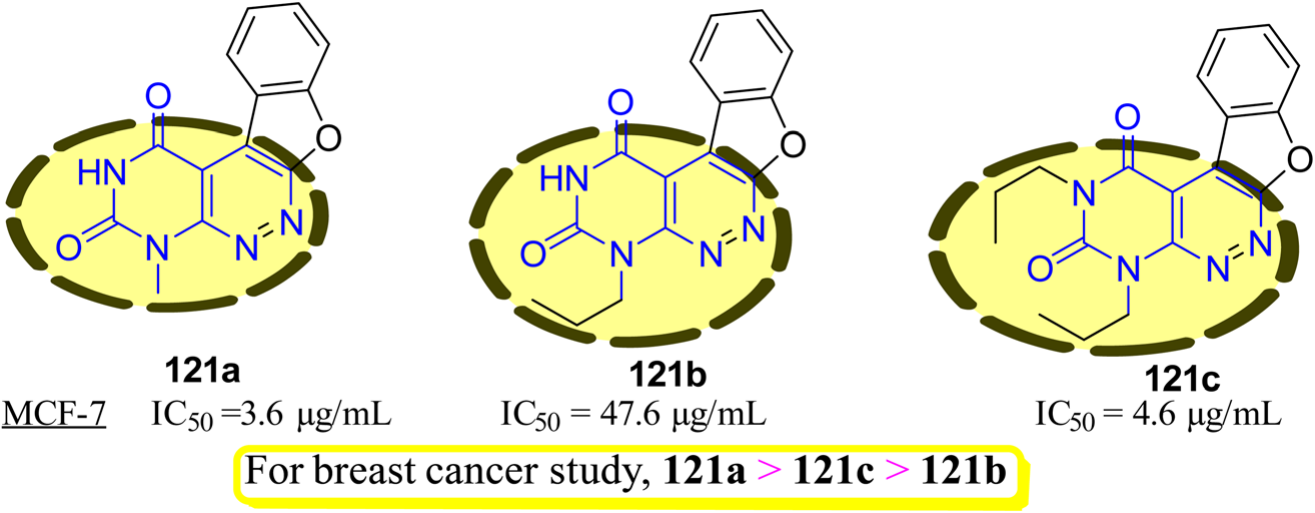
Fused pyrimidine derivatives 121a–c with anticancer properties.

Using a colorimetric viability test, the synthesized compound's 121a–c growth-inhibiting efficacy *in vitro* was compared to the popular anticancer medication 5-flourouracil under the same circumstances. When compared to the standard medication, molecule 121a was the most potent against the breast cancer cell line (MCF-7), with IC_50_ values of 3.6 µg mL^−1^ (Fig. 10), while the IC_50_ value of 5-FU is 4.1 µg mL^−1^.^67^ According to a SAR investigation, 121a's structure may enhance hydrogen bonding with the target site, 121b's modification reduces planarity, weakening binding, and 121c retains partial activity, suggesting intermediate structural compatibility.

**Fig. 11:**
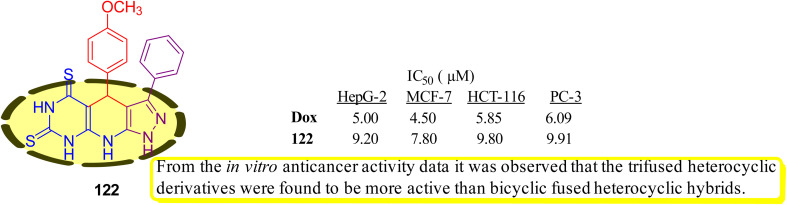
Tricyclic fused pyrimidine 122 containing anticancer activity.

Compound 122 was determined to be the most powerful analogues against the HePG-2, MCF-7, HCT-116, and PC-3 cell lines. Doxorubicin, one of the most widely used and potent anticancer drugs, was employed as a positive control (IC_50_ = 5 µM, 4.5 µM, 5.85 µM, and 6.09 µM, respectively). Compound 122 was 1.84, 1.73, 1.67, and 1.62 times as effective against HePG-2, MCF-7, HCT-116, and PC-3, respectively, as doxorubicin (Fig. 11).^139^ According to SAR research, pyrimidine derivatives with fused system exhibited anticancer properties.

**Fig. 12:**
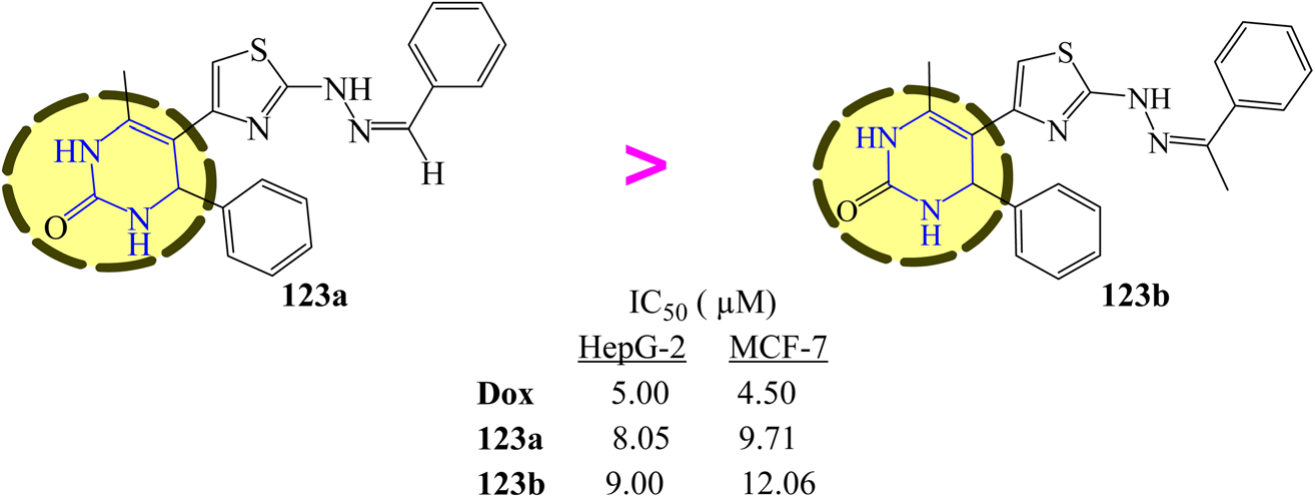
Representative *in vitro* anticancer studies of thiazolyl–pyrimidine hybrids 123a–b.

Lower IC_50_ values showed that HepG-2 cells tend to be more effective than MCF-7 for the majority of substances that are comparable to doxorubicin. For example, the methyl substitution of pyrimidine analog 123b (9.00 ± 0.38 µM for HepG-2 and 12.06 ± 9.00 µM for MCF-7) was weaker than 123a (8.05 ± 0.59 µM for HepG-2 and 9.71 ± 8.05 µM for MCF-7) (Fig. 12).^140^ Planarity of 123a > 123b, which has an electronic conjugation effect that enhance activity, according to SAR studies.

**Fig. 13:**
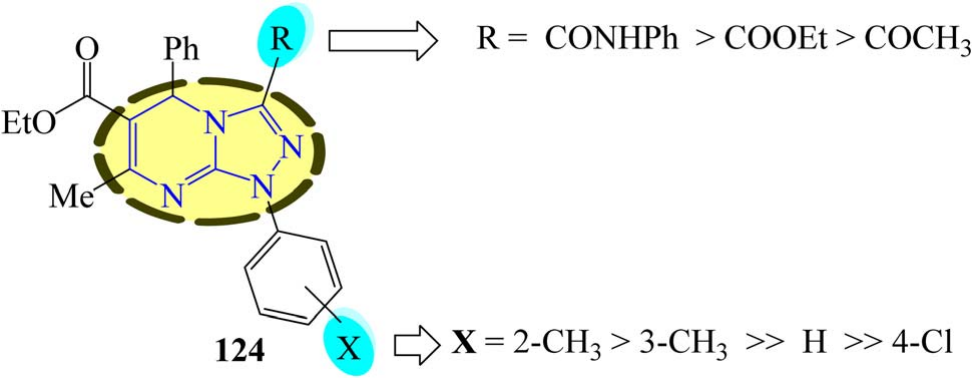
Representative *in vitro* HepG-2 and MCF-7 cell lines of azolo-pyrimidine hybrids 124.

The triazolopyrimidines 124's cytotoxic assessment against HepG-2 and MCF-7 cells demonstrated specific activity patterns that offer important structure–activity insights (Fig. 13). Overall, the potency of compounds 124 varied, with IC_50_ values ranging from >20 µM to low micromolar using doxorubicin as the standard medication.^141^

**Fig. 14:**
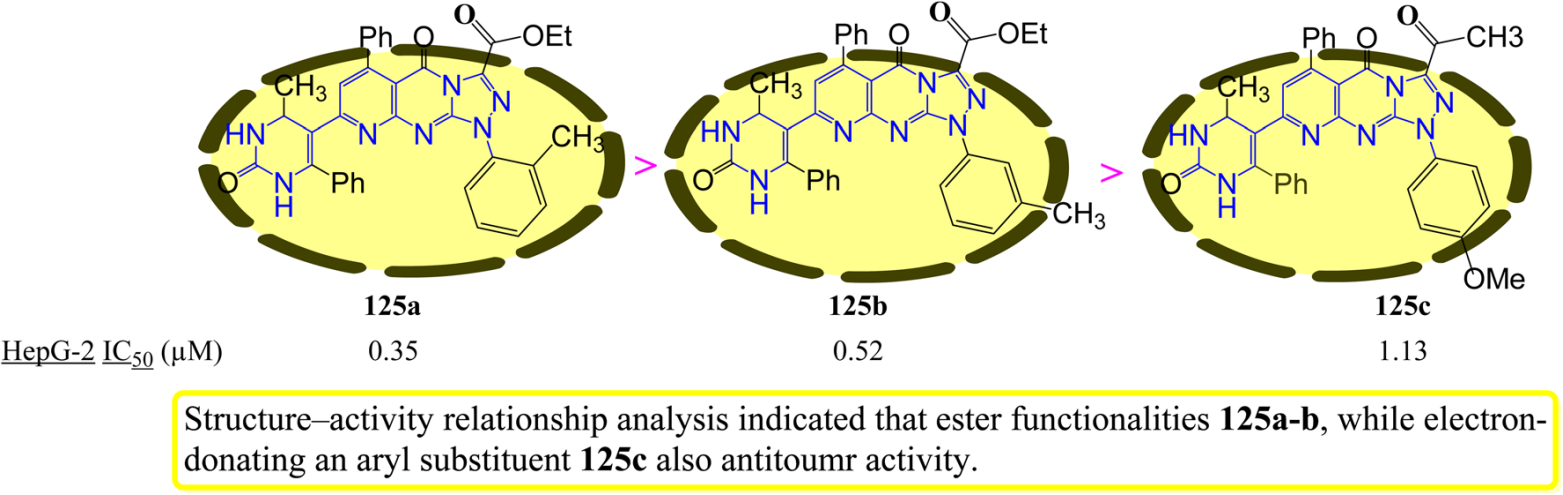
Representative *in vitro* HepG-2 cell lines of pyrido[2,3-*d*]azolopyrimidinone hybrids 125a–c.

According to structure–activity relationship analysis, potency is increased when ester functionalities (COOEt) are combined with ortho- or meta-substituted aryl groups, as in 125b (IC_50_ = 0.52 µM) and 125a (IC_50_ = 0.35 µM). Additionally, electron-donating substituents, such as OMe in 125c (IC_50_ = 1.13 µM), moderate activity in comparison to electron-withdrawing groups (Fig. 14), with activity profiles that match those of doxorubicin (IC_50_ = 0.31 µM).^142^

#### 3.2.2 Inhibitory action of α*-*amylase *in vitro*

**Fig. 15:**
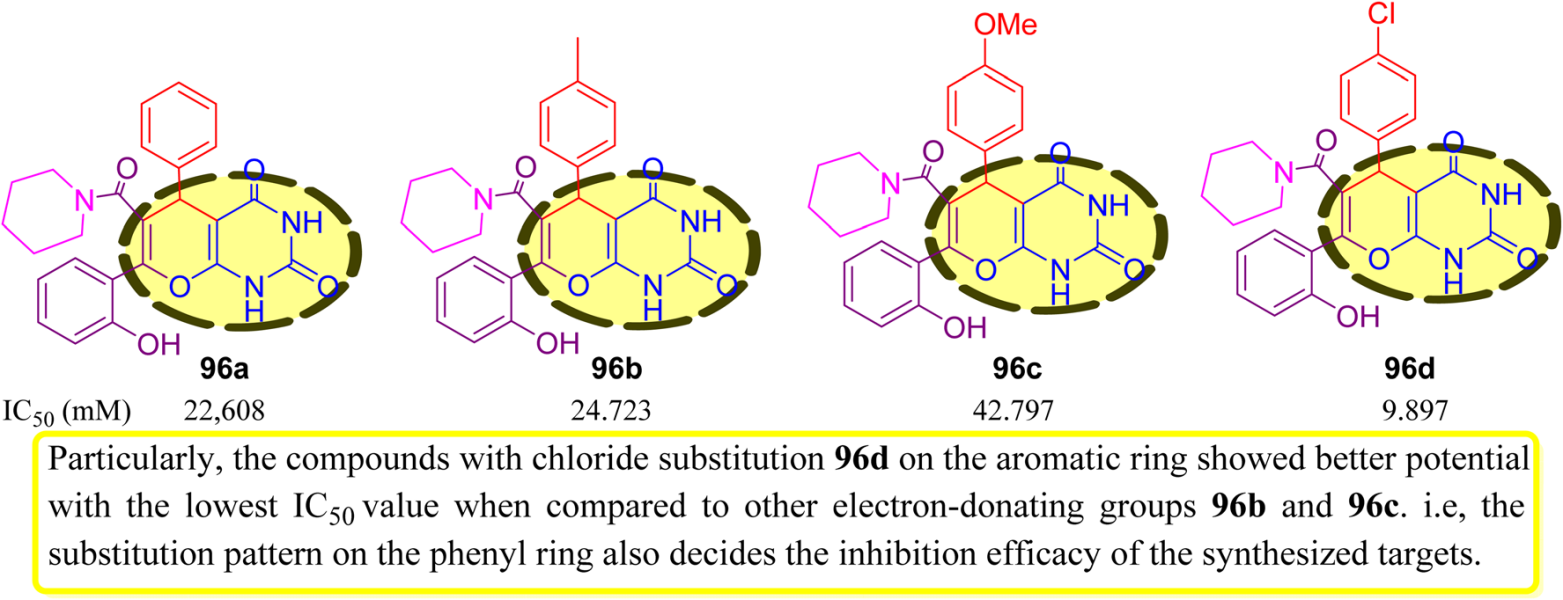
Representative *in vitro* α*-*amylase inhibitory studies of pyrano[2,3-*d*]pyrimidine-2,4*-*dione analogues 96a–d.

The compound with an electron-withdrawing halogen, 96d, showed potent activity in the series 96a–d, but compounds 96b and 96c showed a significant reduction in the enzyme inhibitory effect with electron-donating groups (CH_3_ and OCH_3_), as shown in Fig. 15. When compared to acarbose (IC_50_ = 0.2137 mM), a common α-amylase inhibitor, compound 96a, which had no substitution on the aromatic ring, had minor efficacy.^114^ The *p*-methoxyphenyl scaffold 96c exhibited less productive anticancer properties, according to the structural activity relationship (SAR). At this point, anticancer activity was reduced when electron-donating group (methyl) 96b was substituted for phenyl moiety.

#### 3.2.3 Antimicrobial activity

**Fig. 16:**
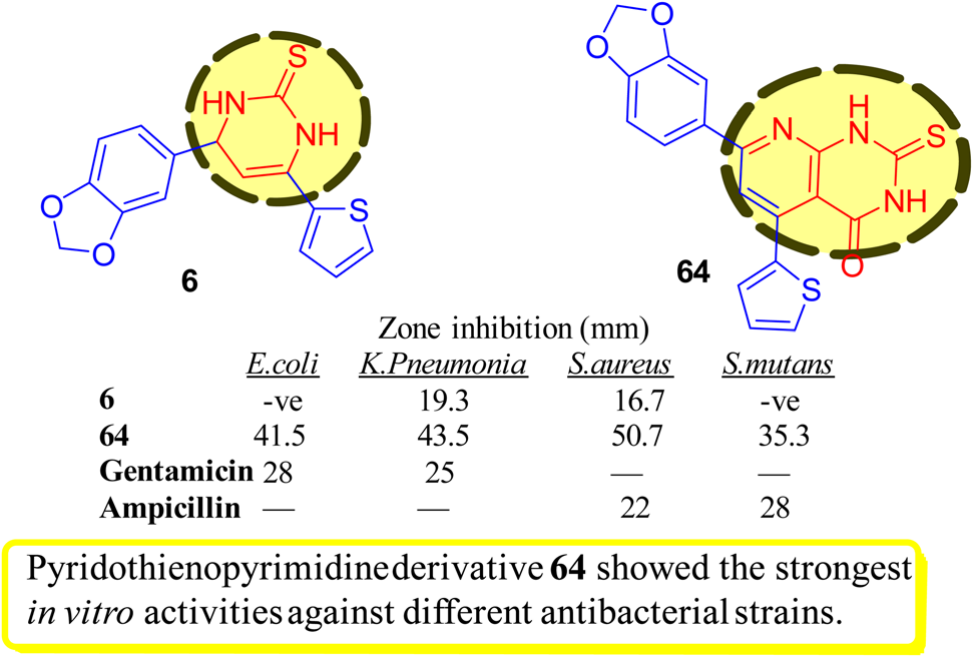
Representative antibacterial studies of pyrimidine 6 and their related fused heterocyclic 64 analogue.

Additionally, pyridopyrimidine 64 has greater activity than pyrimidine 6 when compared to reference drugs (gentamicin for Gram-negative bacteria, which tested for *E. coli* and *K. pneumonia*, and ampicillin for Gram-positive bacteria, which focused on *S. aureus* and *S. mutans*) (Fig. 16).^56^ Fused system improved the molecules' activity, according to a SAR investigation, in which they additionally contributed to making the pyrimidine ring more lipophilic.

**Fig. 17:**
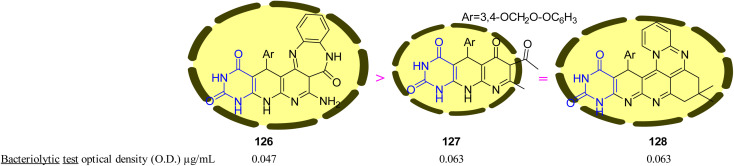
Fused pyrimidine scaffolds 126–128 with its *E. coli* strain.

Several compounds containing the uracil moiety showed promise against a wide spectrum of microorganisms (Fig. 17). Compounds 126–128 had high antibacterial efficiency towards *E. coli*, according to the screening data; the basic antibiotic was ciprofloxacin. The –NH– and the –O– atoms may attach to the negatively charged phosphate group on the phospholipids found on the bacterial wall. Because the phospholipid bilayer's negative charges are neutralized, lysosomal phospholipase activities are inhibited, perhaps resulting in antibacterial action.^79^ According to SAR, the hybrids have more –NH– and the –O– atoms (126) have potent antibacterial activity.

**Fig. 18:**
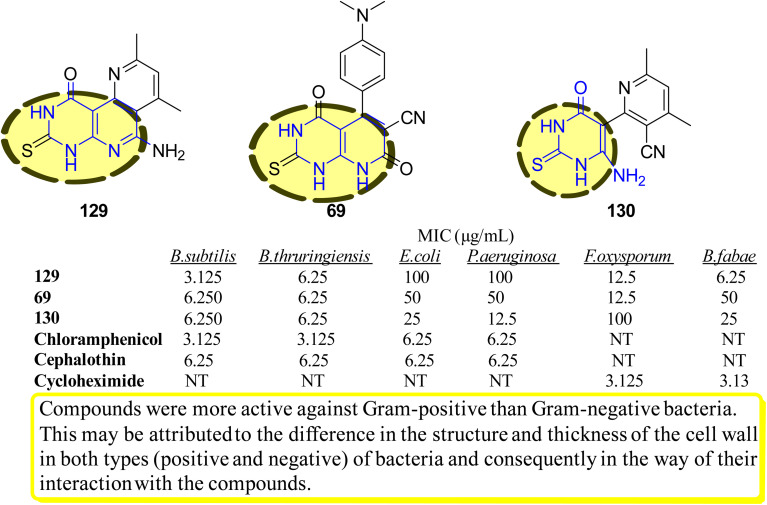
Thiopyrimidine 69 and 129–130 with their antimicrobial evaluations.

Compounds 69 and 129 demonstrated a broad-spectrum antibacterial characteristic toward the investigated species based on the fused pyrimidine analogues' structure–activity relationship towards Gram-positive bacteria. Molecules 69 and 130 have the same potency as cephalothin in blocking the growth of *B. subtilis* and *B. thuringiensis* (MIC 6.25 µg mL^−1^), but their MICs were two times higher (6.25 µg mL^−1^) than those of chloramphenicol (MIC 3.125 µg mL^−1^). Compound 129 was 25% less active than cycloheximide against *F. oxysporum* (MIC 12.5 µg mL^−1^) and 50% less active than cycloheximide in the growth inhibition of *B. fabae* (MIC 6.25 µg mL^−1^) (Fig. 18).^91,94^

**Fig. 19:**
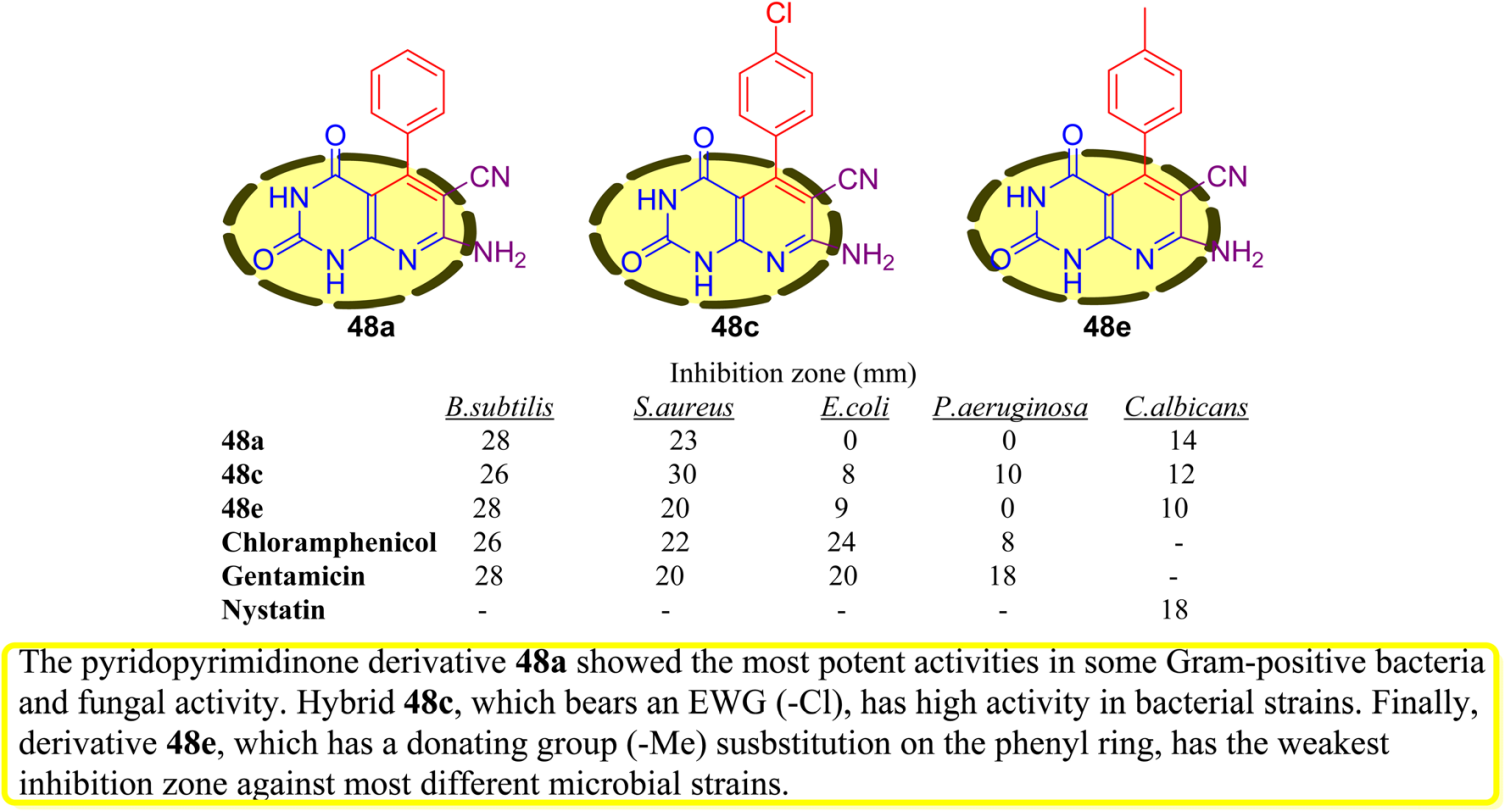
Antibacterial and antifungal activity of compounds 48a, 48c, and 48e.

Fig. 19 was followed in the presentation of compounds 48a, 48c, and 48e, which have antibacterial properties against fungi as well as Gram-positive and negative bacteria, which contrasted with three commercial antibiotics, including nystatin, gentamicin, and chloramphenicol. Molecules 48a, 48c, and 48e exhibit activity against *S. aureus* and *B. subtilis*, while compound 48c exhibits activity towards *E. coli.* Additionally, molecules 48a, 48c, and 48e exhibit potency towards *Candida albicans*, whereas molecule 48c exhibits sensitivity towards *P. aeruginosa*.^143^ As illustrate SAR, electron-withdrawing substituents enhance antimicrobial activity, while electron-donating groups reduce it.

**Fig. 20:**
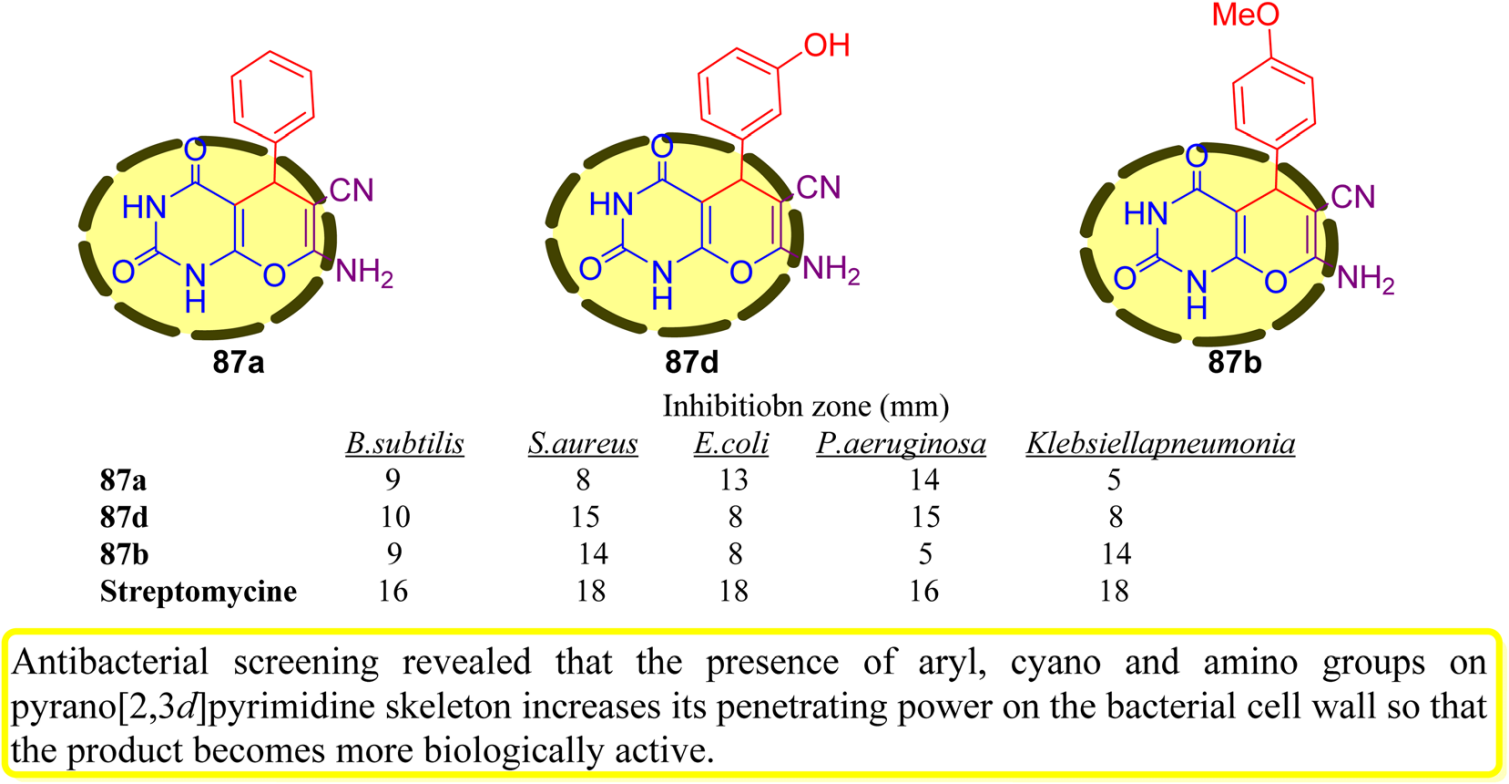
Pyrano[2,3-*d*]pyrimidine hybrids 87a–b and 87d with its promising antibacterial character.

It was shown that substituents had a linked electrical impact on the level of antibacterial properties. The observation that the phenyl (87a), OH (87d), and OCH_3_ (87b) groups are crucial for antibacterial activity against certain Gram-positive and Gram-negative bacteria, including *Pseudomonas aureus*, *E. coli*, *Staphylococcus aureus*, *Klebsiella pneumonia*, and *Bacillus cereus*, is remarkable. This demonstrates the broad impact of bactericidal action on the membrane potential. According to the appropriate studies, the aryl pyrano[2,3-*d*]pyrimidine's moieties, electronic, and polar characteristics were crucial for its antibacterial activity. These results imply that the substances functioned outside the cell and adhered to the bacterial cells' surface groups rather than cracking cell membranes.^104^ Additionally, electron-donating groups decrease antibacterial activity, whereas a hydroxyl substituent has a stronger effect than the methoxy group, thus, 87d is higher than 87b in most bacterial strains, as revealed by SAR (Fig. 20).

**Fig. 21:**
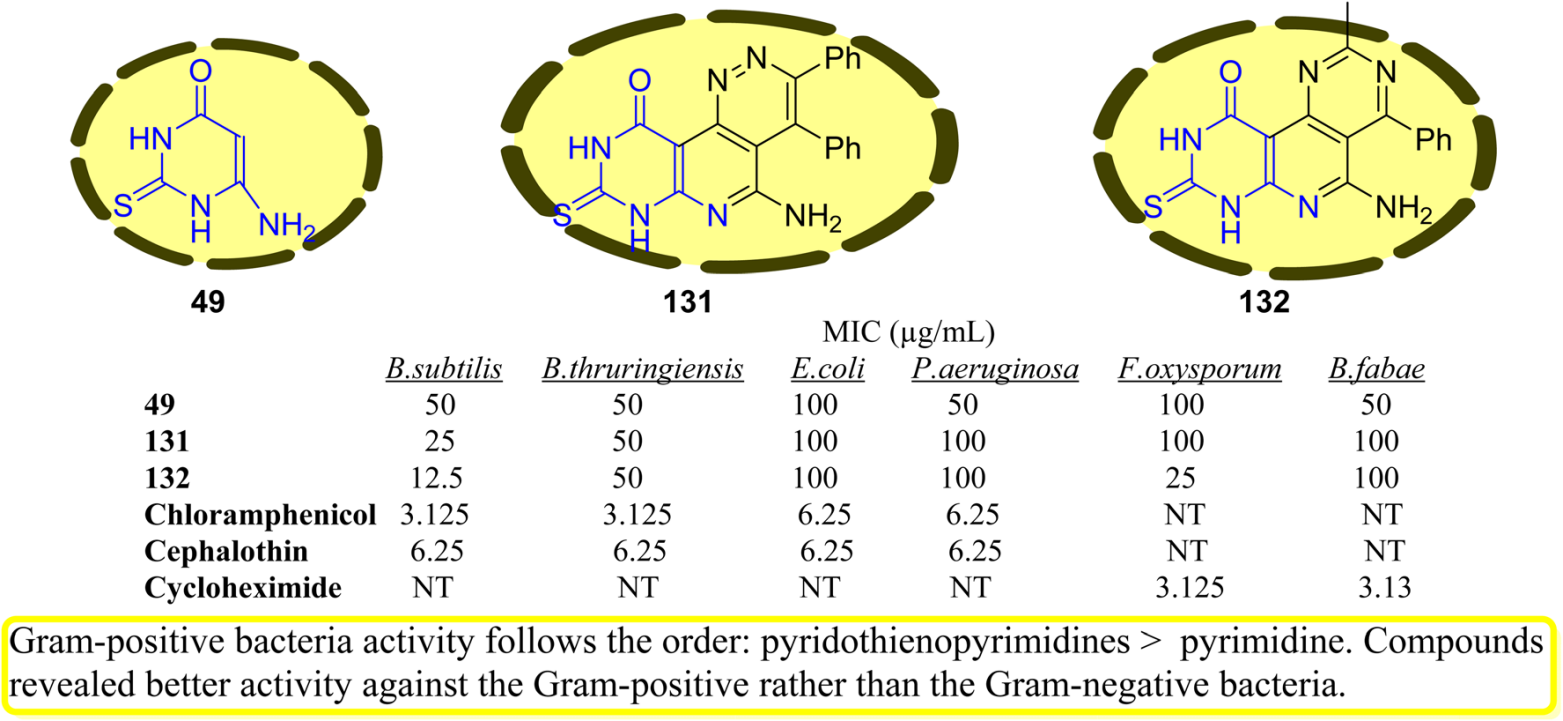
Thiopyrimidine scaffolds 49 and 131–132 with their *in vitro* antmicrobial studies.

Compounds 49 and 131–132, on the other hand, as evidenced by their MIC values, moderately inhibited the growth of Gram-positive bacteria (12.5–50 µg mL^−1^). The other compounds, including 132, demonstrated potent development-inhibitory characteristics against *B. subtilis* (MIC 12.5 µg mL^−1^), which were approximately 25% of the inhibitory effect of chloramphenicol and 50% of the activity of cephalothin towards the same organism. When starting, component 49 was tested against Gram-positive bacteria, it showed a low growth inhibitory effect (MIC 50 µg mL^−1^) (Fig. 21).^91^ The findings show that the sequence of the Gram-positive bacteria activity is as follows: pyridothienopyrimidines > pyrimidine.^56^ The SAR findings are 132 > 131 > 49 for Gram-positive and some antifungal activity. Thus, structural phenyl with additional substituents enhances potency, while most Gram-negative bacteria remain resistant.

**Fig. 22:**
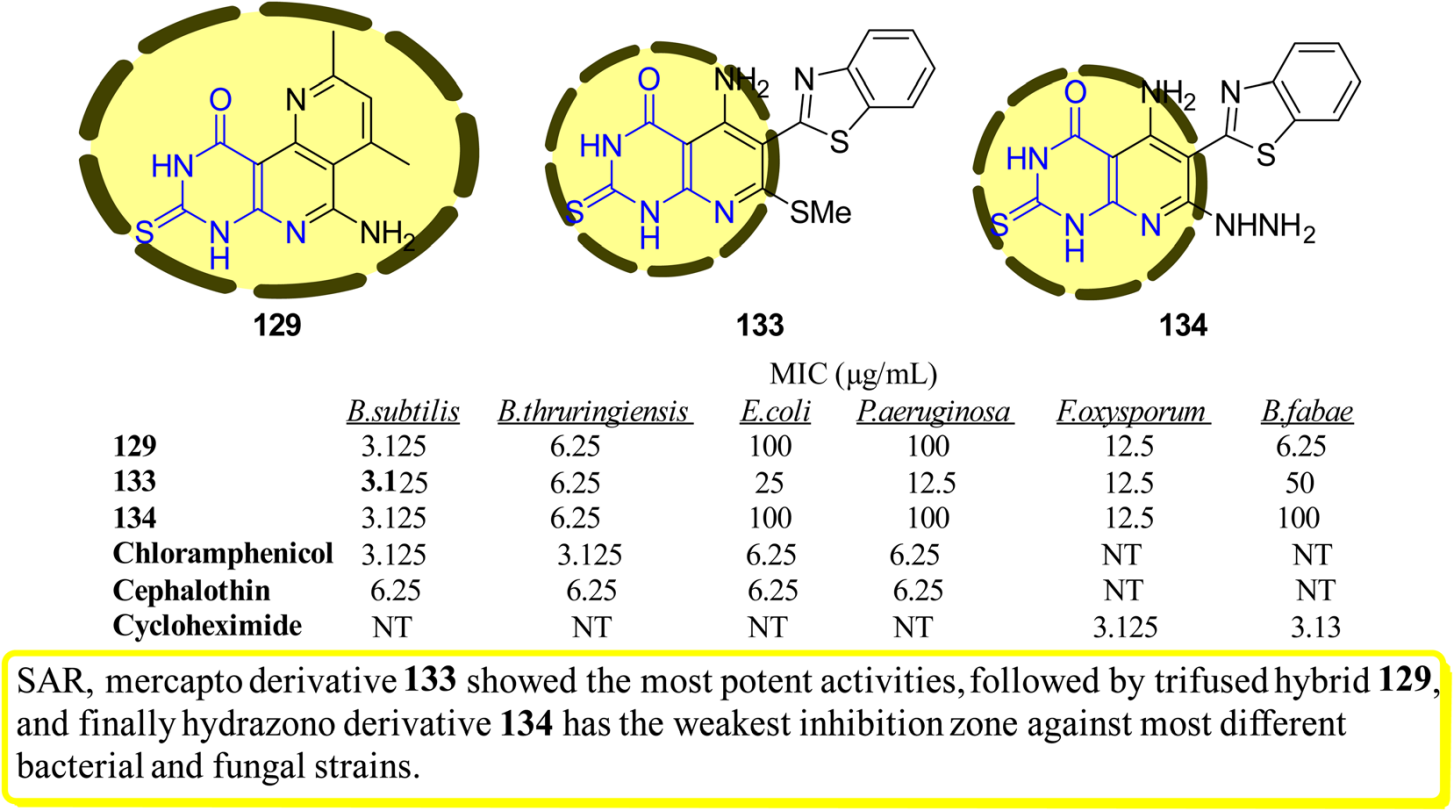
Pyrido–pyrimidine moieties 129 and 133–134 with their antimicrobial activity.

Antifungal activity was observed in some of the presented compounds 129 and 133–134 shown in Fig. 22. Compound 129 was 50% less active than cycloheximide in inhibiting *B. fabae* growth (MIC 6.25 µg mL^−1^), whereas compounds 129 and 133–134 were 25% less active than cycloheximide in inhibiting *F. oxysporum* growth (MIC 12.5 µg mL^−1^).^91^

**Fig. 23:**
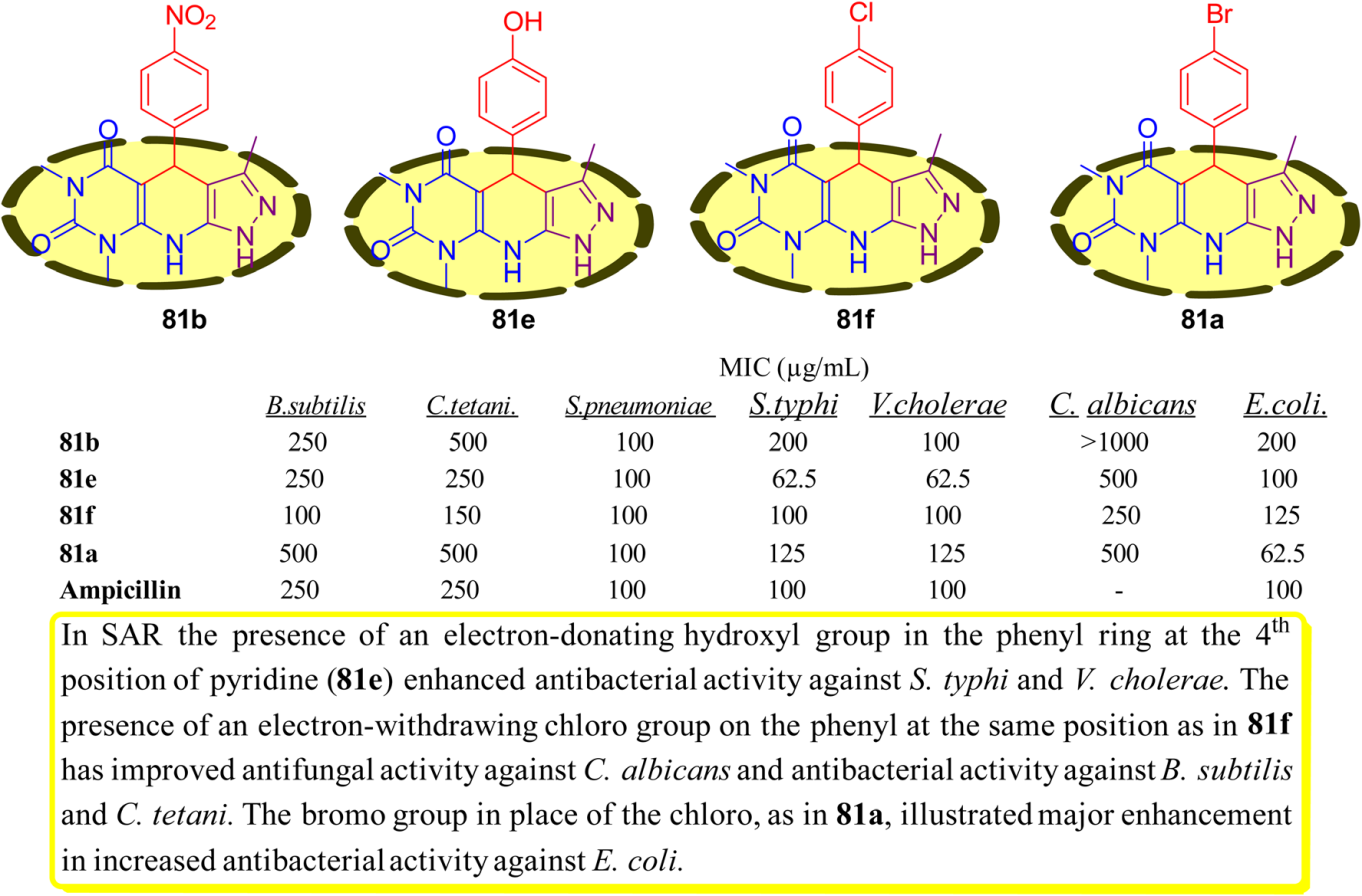
Antimicrobial screening results comparison of pyrazole based pyrido[2, 3-*d*]pyrimidine-diones 81a–b and 81e–f.

When the antibacterial screening data of compounds 81e, 81f, 81a, and 81b were compared to ampicillin (MIC = 250 µg mL^−1^), 81f was found that most of the substances were the most effective towards Gram-positive bacteria *B. subtilis* and *C. tetani*. Fig. 23 showed these compounds had the same potency against *S. pneumoniae*, *i.e.*, 100 µg mL^−1^.^144^

#### 3.2.4 Antiviral activity

**Fig. 24:**
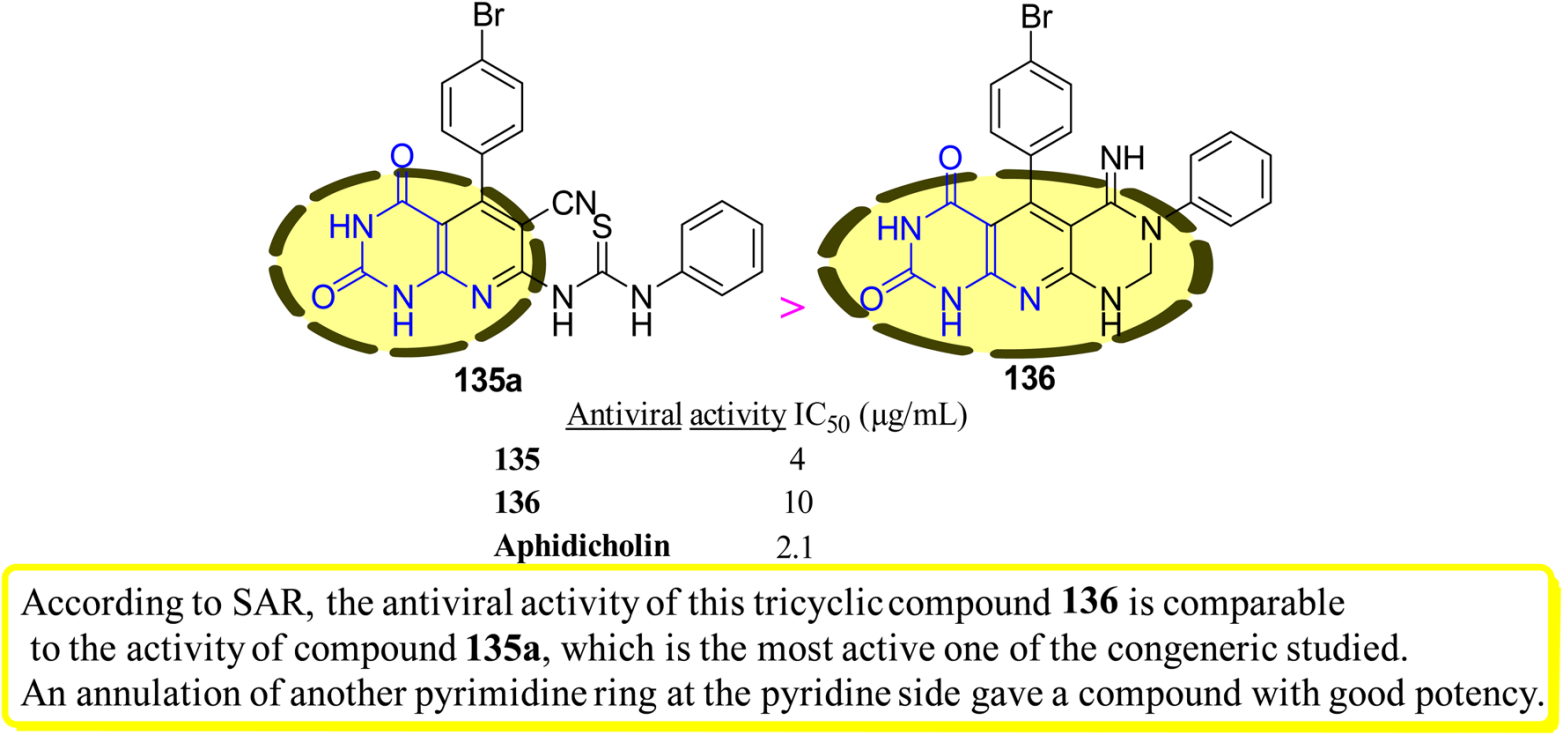
Antiviral activity of fused compounds 135a–136.

Compounds 135a–136, which are fused tricyclic, were discovered to have significant antiviral action (Fig. 24). So, the existence of a substituent with two nitrogen atoms divided by one carbon atom or an additional pyrimidine ring joined to the pyridine nucleus moved the antiviral activity toward greater potency.^80^

**Fig. 25:**
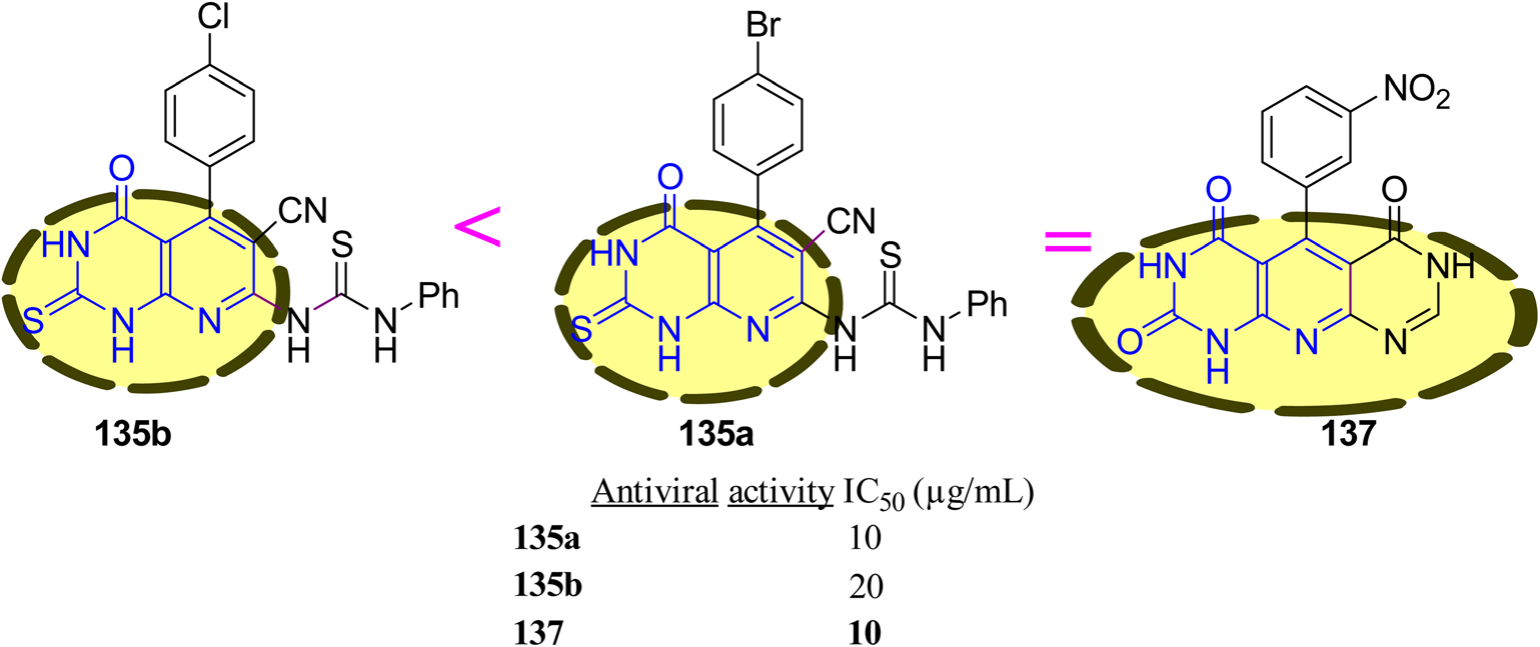
Pyrido[2,3-*d*]pyrimidines 135a–b and 137 hybrids with herpes simplex virus (HSV) properties.

In terms of antiviral activity, as shown in Fig. 25, compounds 135a and 137 with a 4-bromo group was found to be the most efficacious of the test compounds, followed by molecule 137b having a 4-chloro substituent in comparison with aphidicholin.^80^ EWG substituents and fused pyridopyrimidine scaffolds are crucial for target antiviral action. While the bromo group has a stronger EWG effect than the chloro group, hybrid 135a is higher than 135b, according to SAR studies.

**Fig. 26:**
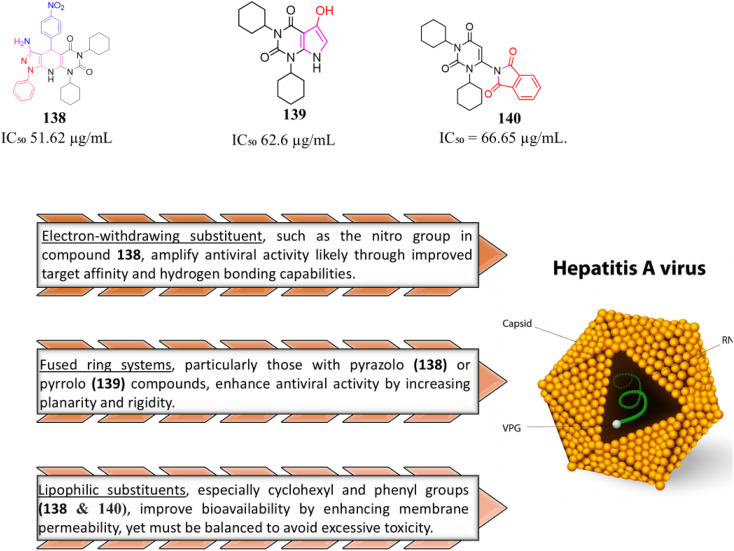
Pyrimidinone scaffolds 138–140 with HAV properties.

Compound 138 demonstrated the greatest action at non-toxic concentrations (64.64% inhibition) when the antiviral efficacy was verified biologically using MTT evaluations on Vero cells, which is compared to acyclovir (95.14%) as a reference drug. Other analogs, such as 139 and 140, also demonstrated notable inhibition (Fig. 26).^145^

#### 3.2.5 Anti-inflammatory activity

**Fig. 27:**
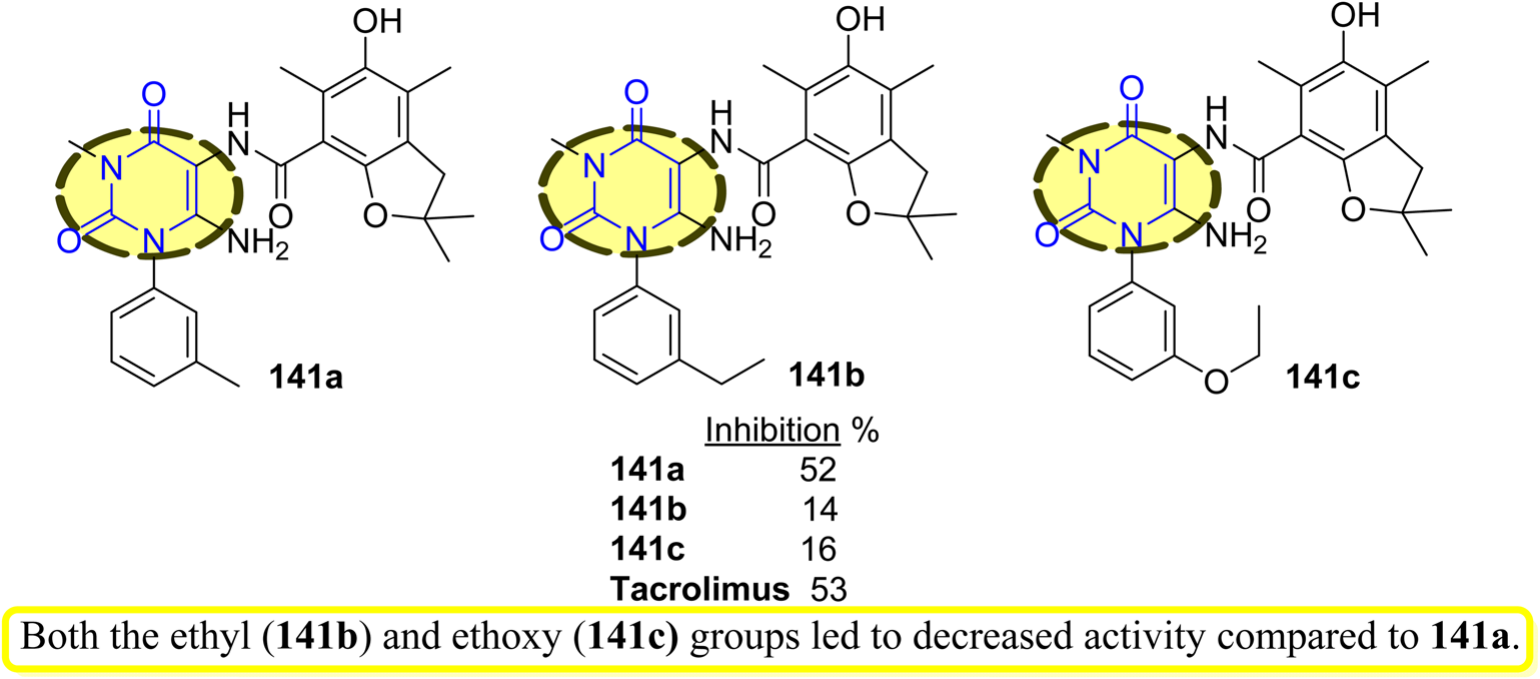
1-Aryl-6-aminouracil derivatives 141a–c with hydroxyl radical-scavenging properties.

Compound 141a, which showed a strong anti-inflammatory effect, has high hydroxyl radical-scavenging activity, reduced substance P was more effective than oxatomide in causing pruritus in mice. The anti-inflammatory activity of 141a was comparable to tacrolimus, a commonly used dermatological medication. With 141a, with strong inhibitory activity, we then concentrated on 3-substitution on the phenyl group (Fig. 27).^146^ The SAR study is 141a (methyl) ≈ tacrolimus > 141c (ethoxy) ≈ 141b (ethyl). Thus, minimal steric bulk and balanced lipophilicity are key for maintaining inhibitory potency.

**Fig. 28:**
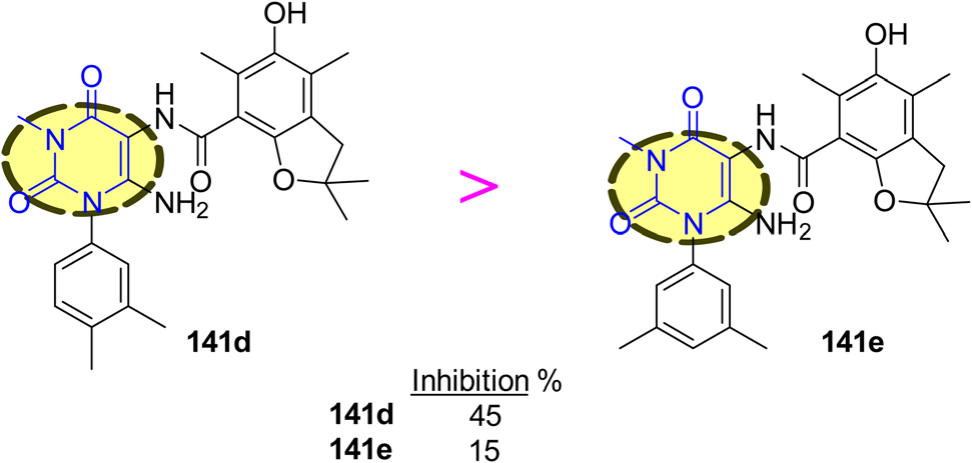
Dimethyldihydrobenzofuranol (DDB)-based 1-phenyl-6-aminouracil 1411d–e containing inflammatory activity.

The 3,4-dimethyl derivative 141d exhibited a strong anti-inflammatory effect that was comparable to tacrolimus, whereas the 3,5-dimethyl derivative 141e had a weaker effect than 141d (Fig. 28).^146^ According to SAR research, pyrimidine derivatives with a 1,2-dimethyl substitution exhibited stronger anti-inflammatory properties than those with a 1,3-postion of the substitution on the phenyl ring.

**Fig. 29:**
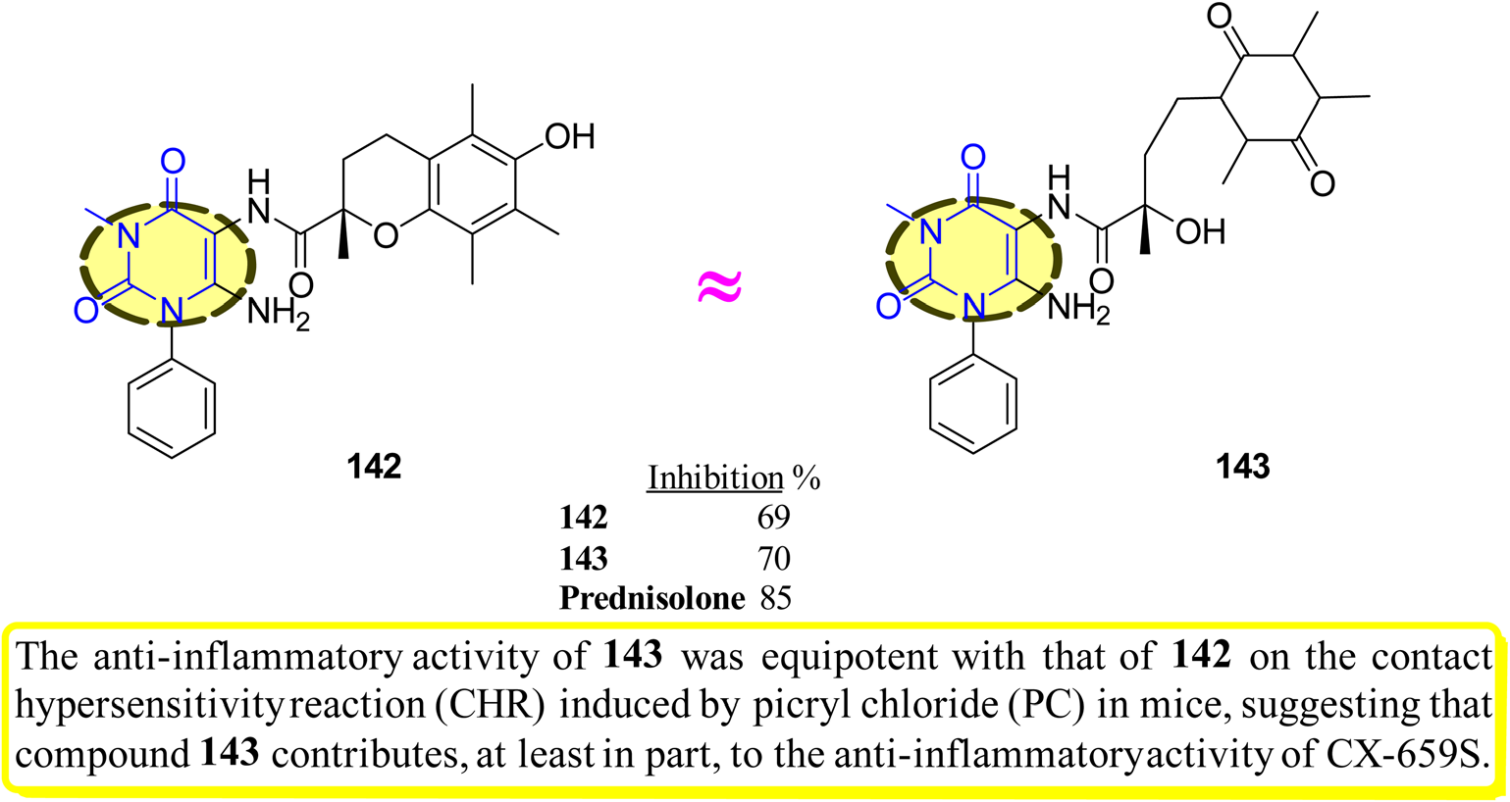
Representative anti-inflammatory studies of pyrimidinediones 142–143.

The potency of 142 was determined to be equivalent to that of 143 as an anti-inflammatory molecule in the comparison with prednisolone (Fig. 29). These data show that this metabolite has a role in CX-659S′ anti-inflammatory properties *in vivo*, at least partially.^147^

#### 3.2.6 Antioxidant activity

**Fig. 30:**
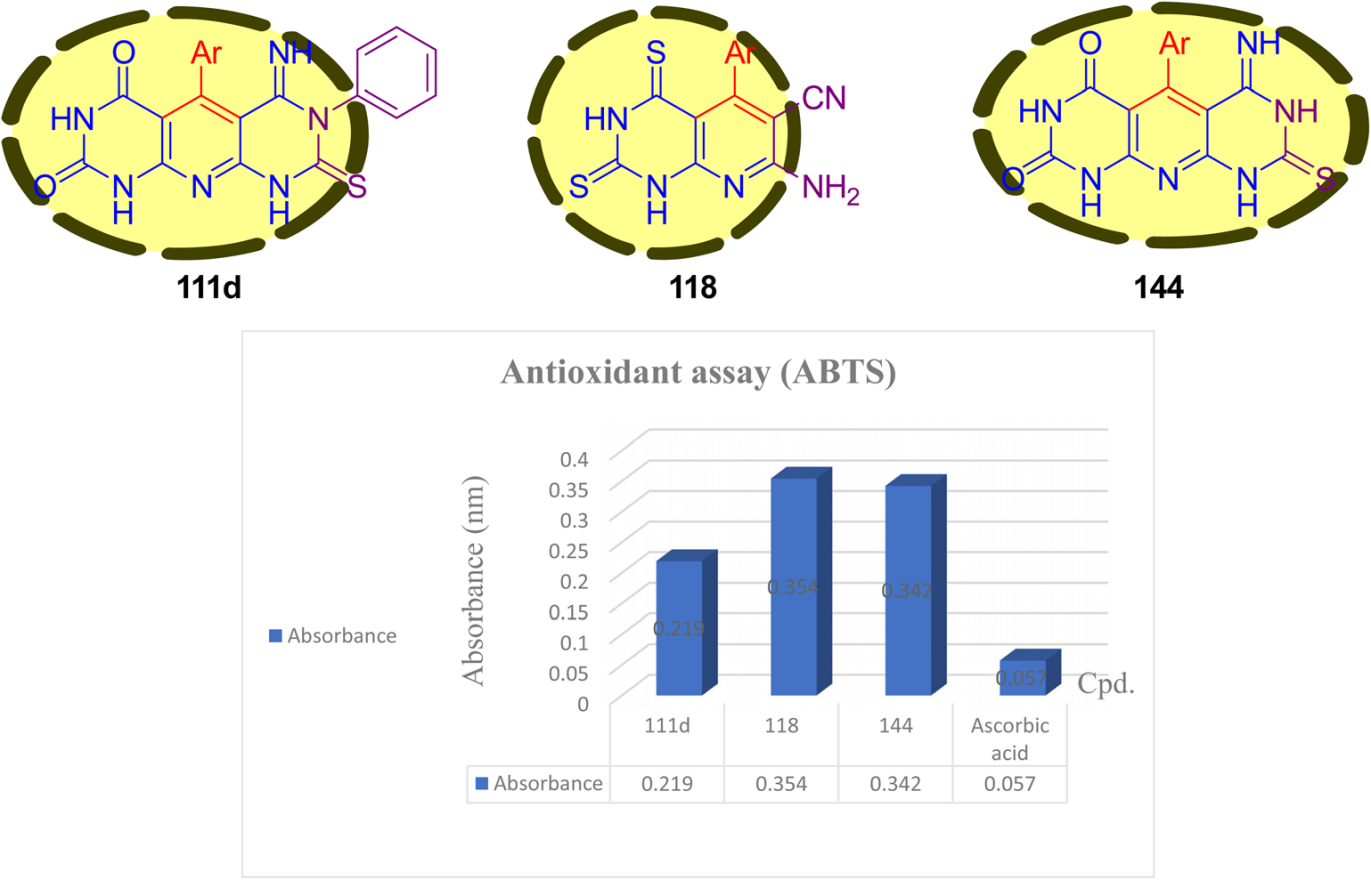
Representative antioxidant studies of fused pyrimidine analogues 111d, 118, along-with 144.

The generalization illustrated in Fig. 30 in comparison to ascorbic acid, compound 111d had the highest antioxidant properties, while compounds 144 and 118 had moderate antioxidant activity, respectively. This could be attributed to the existence of the thioxopyridopyrimidine moiety.^13^ From the *in vitro* antioxidant activity data it was observed that the trifused heterocyclic derivatives were found to be more active than bicyclic fused heterocyclic hybrids. SAR indicated, the more conjugated substituents that enhance radical stabilization without introducing excessive polarity.

**Fig. 31:**
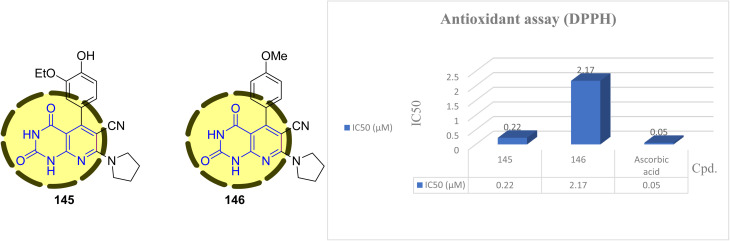
Pyrido[2,3-*d*]pyrimidine Scaffolds 145–146 with antioxidant properties.

The anti-inflammatory properties of the fused pyrido[2,3-*d*]pyrimidines (145–146) were evaluated by Lavanya, Asharani, and Thirumalai (Fig. 31). Studies conducted *in vitro* showed that the compounds' anti-inflammatory effect was comparable to that of diclofenac. When compared to the standard, ascorbic acid, the most potent derivatives, 145 and 146, demonstrated superior antioxidant and anti-inflammatory action.^148^ According to SAR research, pyrimidine hybrids with a disubstitution exhibited stronger anti-inflammatory properties than those with a monosubstitution on the phenyl ring.

#### 3.2.7 Antituberculosis activity

**Fig. 32:**
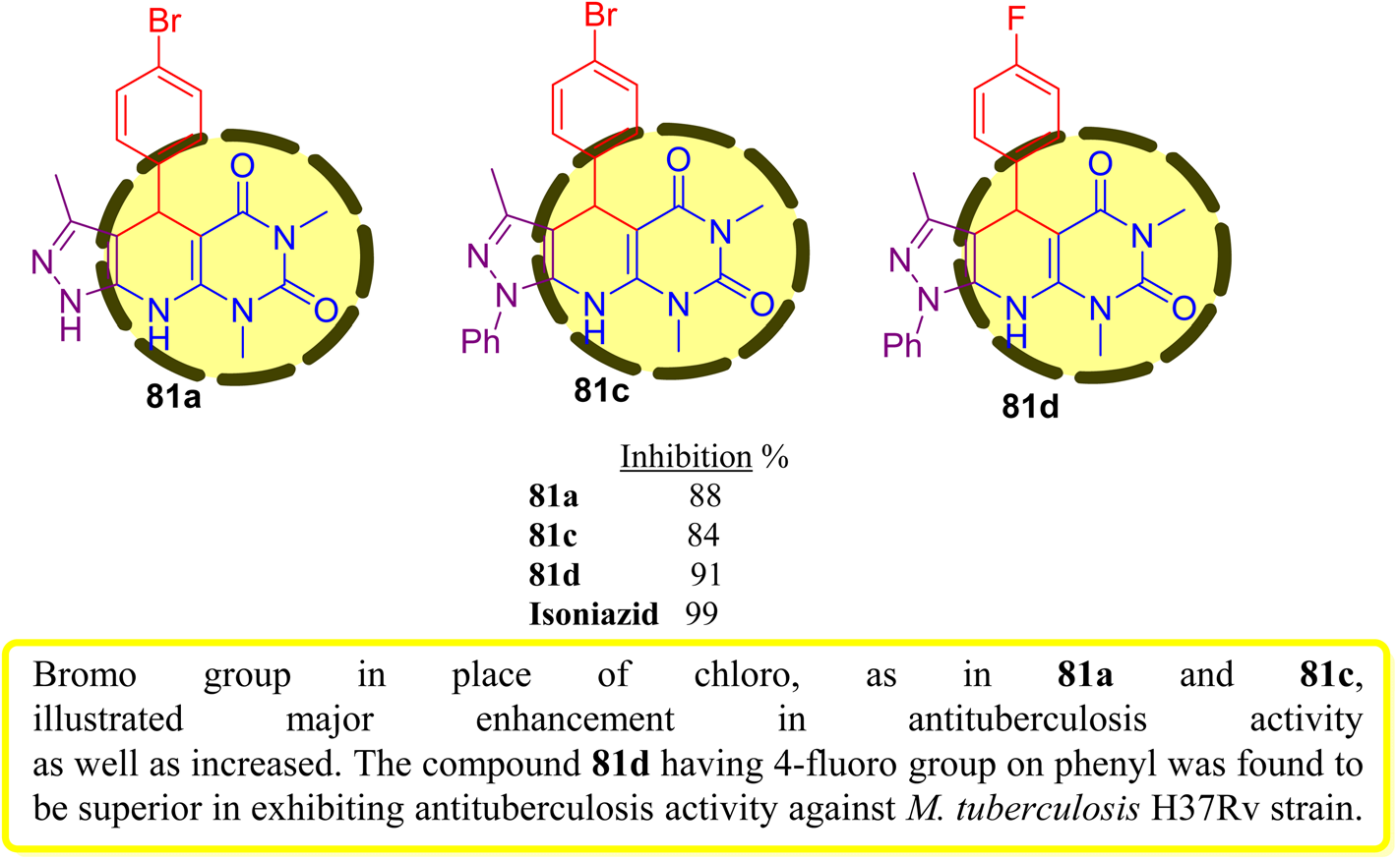
Fused tricyclic pyrimidine hybrids 81a as well as 81c–d with their antitubercular activity.

At a dose of 250 µg mL^−1^, molecules 81a and 81c demonstrated moderate inhibition of 88% and 84%, respectively. In summary, compound 81d provides a new pathway for optimizing this series for a novel class of antitubercular (Fig. 32).^99^ SAR indicated the high molecular weight hybrids that enhance antituberculosis activity. Also, 81a has the ability to form a hydrogen bond than 81c, thus, 81a > 81c in the target activity.

**Fig. 33:**
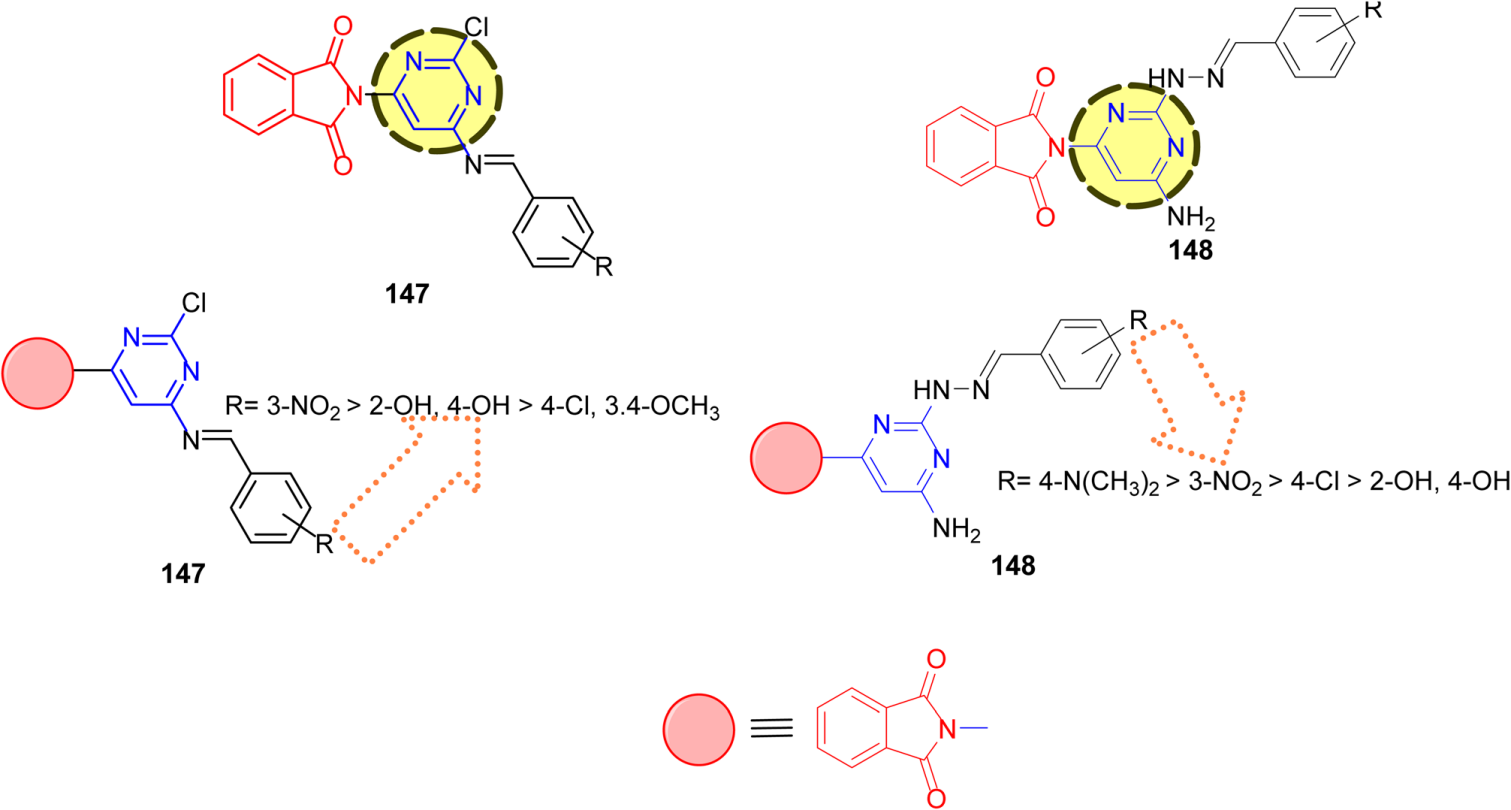
Phthalimide–pyrimidine hybrids 147–148 with their antitubercular activity.

The structural activity relationship (SAR) is the relationship that exists between a structure and its biological activity. This assisted us in determining the substituents at different positions of the pharmacophore (((benzylidene)pyrimidin-4-yl)isoindoline-1,3-dione) and selecting the most effective chemical substitutes to employ in the construction of the hybrid. followed the guideline in Fig. 33.^149^

#### 3.2.8 Antidiabetic activity

**Fig. 34:**
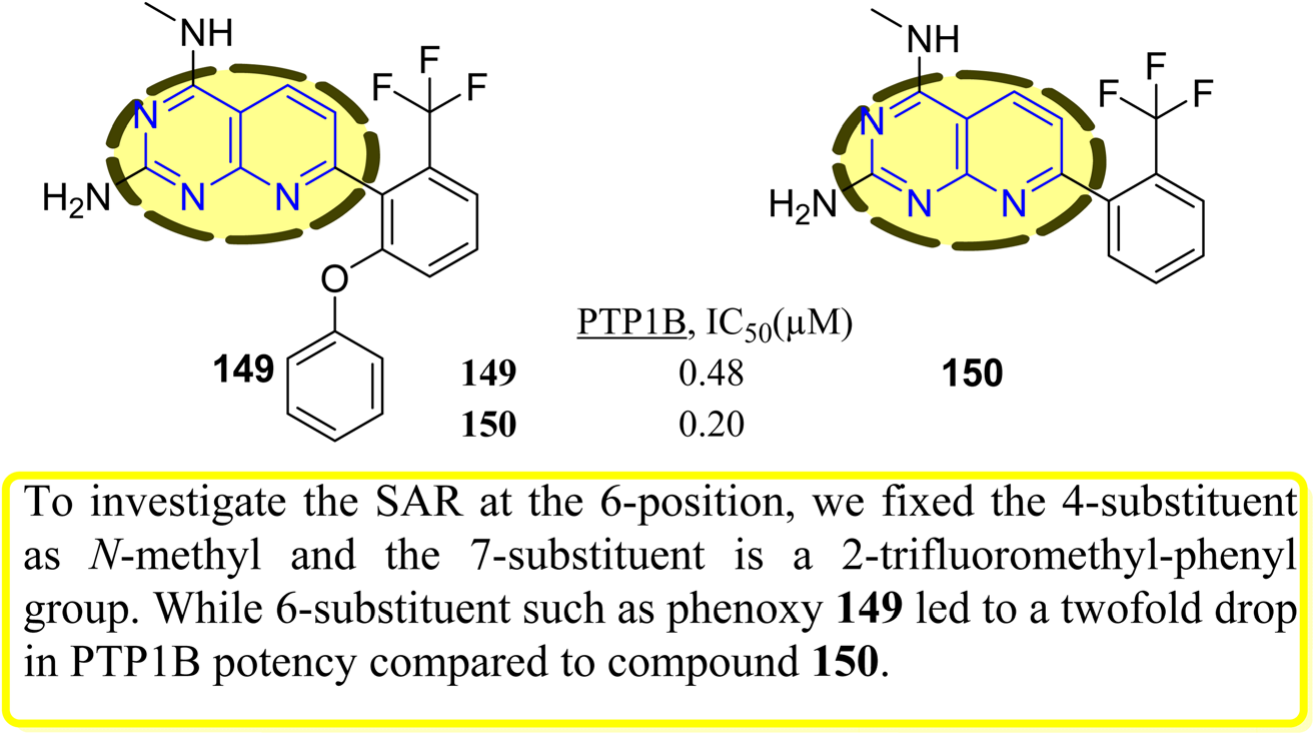
Pyrido[2,3-*d*]Pyrimidine derivatives 149–150 with antidiabetic properties.

The synthesis of pyrido[2,3-*d*]pyrimidine was shown by Cheung *et al.*, who also assessed its pharmacological characteristics as an inhibitor of protein tyrosine phosphatase 1B (Fig. 34). Molecules 149 and 150 were found to be efficacious phosphatase PTP1B inhibitors.^150^

#### 3.2.9 Anticholinesterase activity

**Fig. 35:**
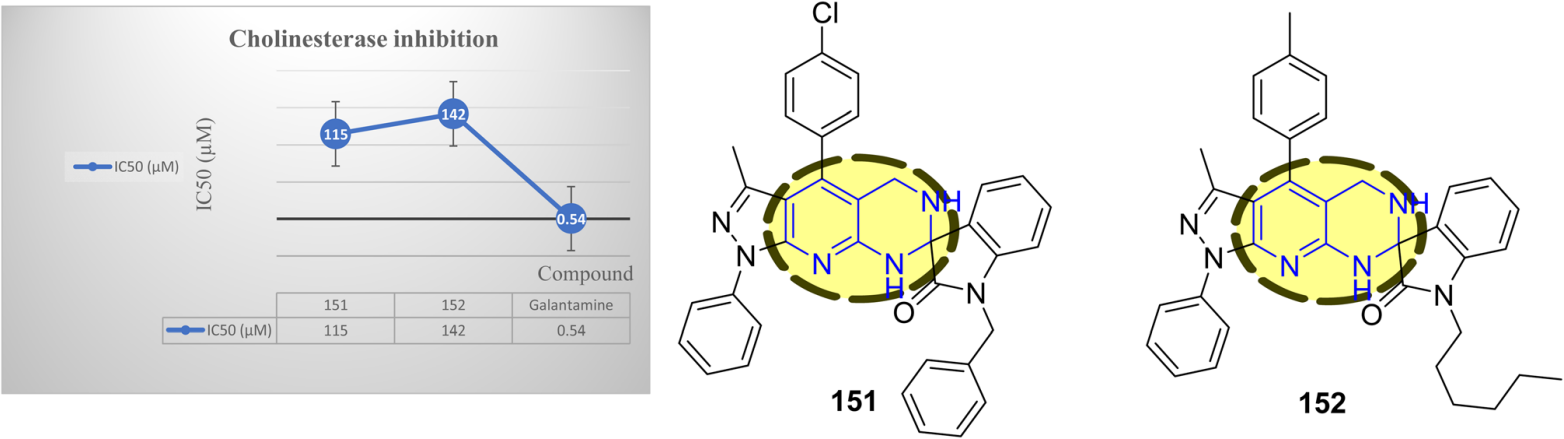
Hybrid pyrido[2,3-*d*]pyrimidines 151–152 with anticholinesterase properties.

The AChE for hybrid pyrido[2,3-*d*]pyrimidine compounds that Acosta and colleagues demonstrated is shown in Fig. 35. 151 and 152 were the series' most active substitutions.^151^ SAR indicated that more conjugated and planar substituents (151) enhance anticholinesterase activity than unconjugated aliphatic scaffolds (152).

#### 3.2.10 Antiplatelet activity

**Fig. 36:**
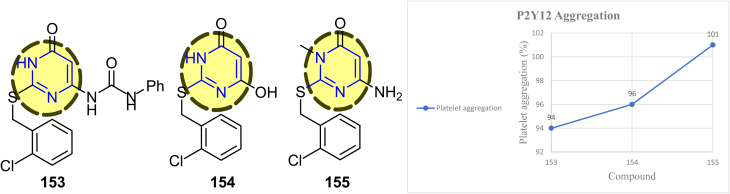
Pyrimidin-4-one hybrids 153–155 with P2Y12 properties.

Compounds 153 and 154 lost their activity compared to the analog 155, indicating that having a free amino group is essential for action (Fig. 36).^65^ The SAR validated 153 (phenylurea) > 154 (hydroxyl) > 155 (amino). Thus, balanced hydrophobic substituents with polarity are optimal for P2Y12 inhibition.

**Fig. 37:**
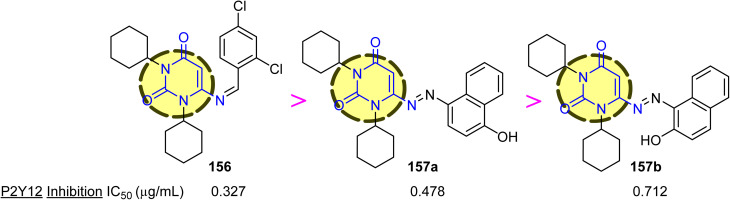
Pyrimidine hybrids 156 and 157a–b with antiplatelet properties.

Compound 156 leads the standard medication clopidogrel (0.327 ± 0.011 µg mL^−1^) on the basis of P2Y12 inhibition, with an IC_50_ of 0.271 ± 0.009 µg mL^−1^. Compounds 157a (0.478 ± 0.016 µg mL^−1^) and 157b (0.712 ± 0.024 µg mL^−1^). The fact that 157a is more potent than 157b indicates that the hydroxyl group increases the IC_50_ value (Fig. 37).^152^ Compound 156 features a dichlorinated phenyl ring, which is linked to a pyrimidine core with a cyclohexane ring, a more lipophilic profile. While compound 157a includes a ((4-hydroxynaphthalen-1-yl)diazenyl) scaffold attached to the same core, enhancing hydrophilicity, compound 157b substitutes position 4 –OH with position 2 –OH, shifting toward slight lipophilicity.

#### 3.2.11 *In vitro* antimalarial activity

**Fig. 38:**
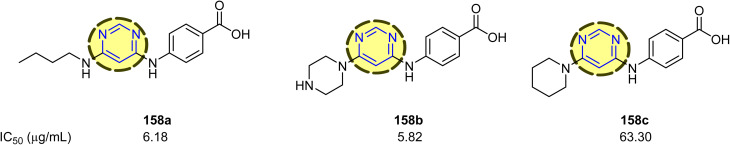
Pyrimidine hybrids 158a–c with antimalarial properties.

In addition to some important pyrimidine derivatives as antimalarial agents, which were presented in a review article in *Tetrahedron*179 (2025) 134626,^153^ we completed the improvement of the scientific depth and currency of the work. The SAR study had observed that the increase in chain length in the case of aliphatic primary amines is directly proportional to enhanced activity, for example, compound 158a with butylamine.^154^ However, secondary cyclic amines 158b showed better activity than primary aliphatic amines. Also, the incorporation of piperidine 158c reduces the activity drastically (Fig. 38).^155^

## 4 Conclusion

Heterocyclic compounds that include nitrogen are a versatile family of substances with useful biological characteristics. The current review focused on numerous methods for producing different substituted N-heterocycles using traditional, multi-component, and microwave-assisted reactions in different conditions along with green chemistry techniques. The most recent studies on the use of pyrimidine analogous in multicomponent Rx. to develop a range of unique substituted N-heterocyclic hybrids have been compiled in this review. In actuality, these MCRs are capable of producing a wide range of complex molecular systems, including a variety of N-heterocyclics, in a single one-pot operational process. It has been discovered that a remarkable synthetic evolution of derivatives of N-heterocycles reveals a variety of pharmacological characteristics. Numerous biological activities, including anticancer, *in vitro* α-amylase inhibitory, antimicrobial, antiviral, anti-inflammatory, antioxidant, antituberculosis, antidiabetic activity, and other uses like anticholinesterase and antiplatelet, have been thoroughly studied for N-heterocycle motifs. Furthermore, we highlighted the SAR of the several pyrimidine-based molecular templates that have been developed by researchers worldwide. Although this overview's examples illustrated the range of synthetic preparation methods utilized to produce N-heterocyclic-based pyrimidine scaffolds and their therapeutic qualities, for various reasons, the research has not been included in this review, for which the authors are apologetic. Continued investigation into the relationship between chemical structure and biological activity, combined with advances in synthetic chemistry and computational modelling, will accelerate the development of pyrimidine-based drugs with increased potency, selectivity, and minimal side effects for a wide range of diseases. Another facet in the discovery of novel and biologically active pyrimidine derivatives is exploring how pharmaceutical synergy may be a useful strategy for increasing the antimalarial potency of novel pyrimidines. Additionally, the current manuscript will assist scientists in discovering new, enhanced, targeted, and selective chemicals.

## Author contributions

Basant Farag, Fouad M. R., and Sobhi M. Gomha contributed to conceptualization, supervision, and writing – review & editing. Basant Farag performed formal analysis and data curation. Basant Farag, Sampath Chinnam, Aamer Saeed, and Sobhi M. Gomha were responsible for investigation, methodology, and validation. Basant Farag and Ghada G. El-Bana led the writing – original draft preparation. All authors read and approved the final version of the manuscript.

## Conflicts of interest

The authors declare that they have no conflicts of interest.

## Data Availability

No primary research results, software or code have been included and no new data were generated or analysed as part of this review.
